# Assembly and comparison of two closely related *Brassica napus* genomes

**DOI:** 10.1111/pbi.12742

**Published:** 2017-06-14

**Authors:** Philipp E. Bayer, Bhavna Hurgobin, Agnieszka A. Golicz, Chon‐Kit Kenneth Chan, Yuxuan Yuan, HueyTyng Lee, Michael Renton, Jinling Meng, Ruiyuan Li, Yan Long, Jun Zou, Ian Bancroft, Boulos Chalhoub, Graham J. King, Jacqueline Batley, David Edwards

**Affiliations:** ^1^ School of Biological Sciences The University of Western Australia Crawley WA Australia; ^2^ School of Agriculture and Food Sciences University of Queensland St. Lucia Qld Australia; ^3^ Plant Molecular Biology and Biotechnology Laboratory Faculty of Veterinary and Agricultural Sciences University of Melbourne, Parkville Melbourne Vic. Australia; ^4^ School of Agriculture and Environment The University of Western Australia Crawley WA Australia; ^5^ National Key Laboratory of Crop Genetic Improvement Key Laboratory of Rapeseed Genetic Improvement Ministry of Agriculture P. R. China Huazhong Agricultural University Wuhan China; ^6^ Department of Biology University of York York UK; ^7^ Organization and Evolution of Complex Genomes (OECG) Institut National de la Recherche agronomique (INRA) Université d'Evry Val d'Essonne (UEVE) Evry France; ^8^ Institute of System and Synthetic Biology, Genopole Centre National de la Recherche Scientifique Université d'Evry Val d'Essonne Université Paris‐Saclay Evry France; ^9^ Southern Cross Plant Science Southern Cross University Lismore NSW Australia

**Keywords:** genome assembly, whole genome comparison, genotyping by sequencing, genome assembly improvement, *Brassica napus*, Tapidor, contigPlacer

## Abstract

As an increasing number of plant genome sequences become available, it is clear that gene content varies between individuals, and the challenge arises to predict the gene content of a species. However, genome comparison is often confounded by variation in assembly and annotation. Differentiating between true gene absence and variation in assembly or annotation is essential for the accurate identification of conserved and variable genes in a species. Here, we present the *de novo* assembly of the *B. napus* cultivar Tapidor and comparison with an improved assembly of the *Brassica napus* cultivar Darmor*‐bzh*. Both cultivars were annotated using the same method to allow comparison of gene content. We identified genes unique to each cultivar and differentiate these from artefacts due to variation in the assembly and annotation. We demonstrate that using a common annotation pipeline can result in different gene predictions, even for closely related cultivars, and repeat regions which collapse during assembly impact whole genome comparison. After accounting for differences in assembly and annotation, we demonstrate that the genome of Darmor*‐bzh* contains a greater number of genes than the genome of Tapidor. Our results are the first step towards comparison of the true differences between *B. napus* genomes and highlight the potential sources of error in future production of a *B. napus* pangenome.

## Introduction

*Brassica* is a genus which contains economically important crop species, including condiments such as mustards; vegetable crops including broccoli, Brussels sprouts, cabbage, cauliflower; and commercially important oilseeds which are used as both a food and biofuel. The genomes of the *Brassica* family are related as described in the triangle of U (Nagaharu, 1935). There are three diploid *Brassica* species, *B. rapa* (AA, *n* = 10), *B. nigra* (BB, *n* = 8), and *B. oleracea* (CC, *n* = 9), and these genomes evolved from a common ancestor which underwent at least one whole genome triplication (Lysak *et al*., 2005). Combinations of these genomes result in three amphidiploid *Brassica* species: *B. juncea* (AABB, *n* = 18), *B. carinata* (BBCC, *n* = 17) and *B. napus* (AACC, *n* = 19). Several *Brassica* genome assemblies have now been published, including the A genome of the *B. rapa* cultivar Chiifu (Wang *et al*., 2011), the C genome of the *B. oleracea* cultivars O2‐12 (Liu *et al*., 2014) and TO1000 (Parkin *et al*., 2014), the AACC genome of the *B. napus* cultivar Darmor*‐bzh* (Chalhoub *et al*., 2014) and the BB and AABB genome of *B. nigra* and *B. juncea* (Yang *et al*., 2016).

Early indications of significant differences in genome content between individuals were apparent from data obtained from RFLP and SSR analysis in *Brassica* species, where a relatively large number of null alleles were detected with co‐dominant molecular assays (Udall *et al*., 2005). With the increased application of next generation DNA sequencing technology, these genomic differences are becoming more apparent and there is a growing understanding that a single genome does not reflect the gene content of a species. Following the production of reference genome assemblies, researchers are moving towards the characterization of pangenomes, representing the gene content of a species, including core genes which are found in all individuals and variable genes which are only present in some individuals (Golicz *et al*. 2016a). Variable genes can be split into two groups: copy number variations (CNVs), in which the number of copies of a gene differs between individuals, and the presence/absence variations (PAVs), an extreme form of CNV in which a gene is present in some individuals but absent in others (Golicz *et al*., 2016a; Saxena *et al*., 2014).

Gene presence/absence variation is important for crop improvement as some variable genes have been shown to be associated with agronomic traits. In rice, comparison of three divergent lines led to the identification of several variable genes including the submergence tolerance gene *Sub1A* which is absent in submergence intolerant lines (Schatz *et al*., 2014). A recent study investigated the pangenome for 1483 rice accessions and found 1913 high confidence dispensable genes, of which 1489 were expressed (Yao *et al*., 2015). A genome‐wide association study (GWAS) with SNPs for those rice accessions found that 23.5% of metabolic traits had higher association signals with SNPs located on dispensable genes than with SNPs located on the core reference genome. Similarly, comparison of three *Brassica rapa* morphological variations (turnip, rapid cycling and Chinese cabbage) revealed around 1224 unique genes in each of the three genomes (Lin *et al*., 2014), while another recent study produced low coverage sequence data for 199 *B. rapa* and 119 *B. oleracea* accessions to identify SNPs and trace parallel selection signals in the two subgenomes (Cheng *et al*., 2016). Regions undergoing positive selection could be identified (25 in *B. rapa* and 58 in *B. oleracea*), of which nine were shared between the genomes.

In chickpea, the re‐sequencing of 16 lines identified up to 32 genes absent in each line (Thudi *et al*., 2016), while a comparison of 503 inbred maize lines demonstrated that only 16.4% of 8,681 representative transcripts were expressed in all lines (Hirsch *et al*., 2014). Recently, Golicz *et al*. (2016a,b) identified 61 379 genes in a *Brassica oleracea* pangenome study, of which 18.7% demonstrated the presence/absence variation among individuals, and with the variable genes being enriched for annotations associated with important agronomic traits.

The repetitive content of plant genomes makes genome assembly and validation a challenge (Edwards *et al*., 2013). Assemblers often cannot accurately assign sequence reads from repetitive regions to their correct genomic location and repeat sequences in the genome frequently collapse into a single copy in the assembly. In addition, different annotation results may confound comparative analyses as some genes may not be predicted in some cultivars even if they share significant sequence identify with genes predicted in other cultivars. These factors impact the direct comparison of whole genome assemblies for complex crop genomes and make the assessment of species pangenomes a major challenge.

Here, we describe the *de novo* assembly and annotation of the amphidiploid *B. napus* cultivar Tapidor and comparison with an improved and reannotated assembly of the published *B. napus* cultivar Darmor*‐bzh* (Chalhoub *et al*., 2014). Comparison of the gene content between Darmor*‐bzh* and Tapidor identified genes that are conserved or unique to each cultivar. In addition, we identified genes which appear to be absent in one or other cultivar due to variation in assembly or annotation. Our results present a detailed assessment and comparison of assemblies for complex *B. napus* genomes, the first step towards establishing a *B. napus* pangenome.

## Results

### The *B. napus* Darmor*‐bzh* and Tapidor genome assemblies

The published Darmor*‐bzh* assembly (Chalhoub *et al*., 2014) consists of 19 pseudomolecules anchored to a high‐resolution genetic map, as well as 19 collections of contigs assigned to a chromosome but not placed, and three collections of contigs that are not assigned to any pseudomolecule. In total, the 19 pseudomolecules have a combined length of 850 Mbp which includes 204 Mbp remaining in 22 unplaced collections. K‐mer‐based predictions for both assembly size and genome size were calculated. Using all Darmor*‐bzh* reads and all Tapidor reads as input, k‐mer‐based genome assembly size prediction estimated an assembly size of 808 Mbp for Darmor*‐bzh* and 697 Mbp for Tapidor, while k‐mer‐based genome size prediction suggested a genome size of 1345 Mbp for Darmor*‐bzh* and 1,335 Mbp for Tapidor.

The SkimGBS pipeline (Bayer *et al*., 2015) was applied to improve the published Darmor*‐bzh* assembly. This predicted 1 006 985 SNPs and called 38 471 969 genotypes across 92 individuals of the Tapidor × Ningyou‐7 population, using the published Darmor*‐bzh* assembly as the reference. Using these data, contigPlacer added 8820 previously unplaced contigs into the Darmor*‐bzh* pseudomolecules, increasing their length by 153 Mbp (23.6%) (Table 1). It was not possible to place 10 981 contigs (51.3 Mbp), of which 10 486 carried no SNPs, and 495 had conflicting pseudomolecule locations. Unplaced contigs placed on two different pseudo‐molecules had an average size of 7244 bp ranging from 2004 bp to 98 348 bp. Of the contigs with a previously assigned pseudomolecule, 98% were placed within their predicted pseudomolecule (Table S1).

**Table 1:** Size, SNPs, predicted genes, absent genes in Darmor*‐bzh* and Tapidor

Name	Darmor*‐bzh* SNPs	Darmor*‐bzh* Length (Mbp)	Darmor*‐bzh* SNPs/Mbp	Tapidor SNPs	Tapidor Length (Mbp)	Tapidor SNPs/Mbp	Darmor*‐bzh* predicted filtered genes	Tapidor predicted filtered genes	Tapidor genes with no Darmor*‐bzh* reads	Darmor*‐bzh* genes with no Tapidor reads
chrA01	45 870	31.16	1472	37 875	23.9	1586	3687	3050	0	3
chrA02	45 229	31.34	1443	46 599	27.9	1672	3528	3315	1	0
chrA03	59 180	39.49	1499	55 319	32.1	1723	5429	4408	0	4
chrA04	57 270	23.31	2457	49 357	21	2351	2676	2638	0	0
chrA05	54 784	28.6	1916	36 548	20.1	1818	3506	2634	1	1
chrA06	72 163	31.9	2262	57 613	29.1	1977	3926	3713	0	0
chrA07	45 458	28.9	1573	34 101	22.7	1505	3555	2976	0	0
chrA08	24 807	21.74	1141	22 096	16.4	1346	2827	2147	0	0
chrA09	79 586	46.72	1704	53 008	30.8	1721	5286	3913	0	3
chrA10	27 723	19.96	1389	35 378	22.5	1575	2800	3175	0	1
Total A	512 070	303		427 894	247		37 220	31 969	2	12
chrC01	92 316	47.95	1925	63 462	31.9	1990	3739	3083	0	5
chrC02	49 665	58.66	847	47 872	40.5	1181	4182	3572	1	23
chrC03	64 224	71.85	894	72 229	55.2	1307	6448	5800	0	9
chrC04	95 329	61.04	1562	79 006	45.6	1733	4658	4356	0	2
chrC05	25 226	52.72	479	43 752	45.6	959	4452	4839	0	4
chrC06	41 036	44.61	920	41 252	34.1	1211	3543	3230	0	6
chrC07	34 089	52.5	649	41 001	37.3	1099	4165	3523	0	1
chrC08	43 857	46.29	947	48 079	40.2	1197	4140	4295	0	0
chrC09	46 660	60.21	775	57 479	51	1127	4607	4864	0	2
Total C	492 402	496		494 132	381		39 934	37 562	1	52
Unplaced contigs	2513	51.33	49	23 380	8.5	2752	3228	631	0	9
Total	1 006 985	850		945 406	636		80 382	70 162	3	73

The *B. napus* cultivar Tapidor was assembled *de novo* using paired end and mate paired Illumina sequencing data with a range of insert sizes. The sequence data were first cleaned prior to assembly. From 417 527 199 Tapidor read pairs, 54 030 058 (12.9%) were identified as clonal and removed. Quality control using sickle (Joshi and Fass, 2011) discarded a further 414 133 read pairs and 2 917 744 single reads, while computational normalization using khmer (Brown *et al*., 2012; Crusoe *et al*., 2015) removed another 134 597 316 read pairs bringing the final number of read pairs for assembly to 258 028 009. These were assembled using VelvetOptimiser (Gladman and Seemann, 2012) in conjunction with Velvet (Zerbino and Birney, 2008) and a k‐mer value of 71. After removal of contigs smaller than 1000 bp, the assembly contained 21 280 contigs with an N50 of 197 031 bp and a total size of 634.19 Mbp.

To produce pseudomolecules for Tapidor, the contigs were sorted by comparison with the improved Darmor*‐bzh* pseudomolecules, placing 18 087 contigs into 19 pseudomolecules with a total length of 616.7 Mbp, with 3193 (19.5 Mbp) unplaced contigs. Using this Tapidor assembly, the SkimGBS pipeline predicted 945 406 SNPs and called 35 829 337 genotypes for the Tapidor × Ningyou‐7 population. ContigPlacer then placed an additional 452 Tapidor contigs into pseudomolecules, bringing the total size of the Tapidor pseudomolecules to 625.9 Mbp, with 2741 contigs (8.2 Mbp) remaining unplaced, none of which contained SNPs (Table 1, Table S1). A comparison of the Darmor*‐bzh* and Tapidor assemblies showed a near perfect overlap, with secondary overlaps due to sequence identity between homeologous chromosomes (Figure S1). A high‐resolution genetic map using the Tapidor × Ningyou‐7 DH population and MSTMap (Wu *et al*., 2008) placed 318.9 Mbp (50.1%) of the Tapidor assembly into 19 linkage groups, and the order of these contigs agreed with the placement of contigs using the Darmor*‐bzh* reference (Figure S2).

To assess and compare the completeness of the two genome assemblies, CEGMA (Parra *et al*., 2007) was used to identify core eukaryotic genes (CEGs), and BUSCO (Simão *et al*., 2015) was used with the plants profile to identify Single‐Copy Orthologs (SCOs). All 248 CEGs aligned completely with the Darmor*‐bzh* assembly, while only 246 CEGs aligned completely with the Tapidor assembly, with the remaining two partially identified in the assembly. Of the 956 BUSCO groups, 925 and 904 appeared at least once in Darmor*‐bzh* and Tapidor, respectively. In Darmor*‐bzh*, 862 BUSCOs appeared more than once with an average copy number of all BUSCOs of 2.2, while for Tapidor, 634 BUSCOs appeared more than once with an average copy number of 1.8. In Darmor*‐bzh*, 26 BUSCOs were missing and five were fragmented, while in Tapidor, 31 BUSCOs were missing with an additional 21 fragmented. RepeatModeler identified 310 192 450 bp (36.48%) repeats in Darmor*‐bzh* and 223 636 559 bp (35.15%) repeats in Tapidor. LTR/Gypsy was the most common repetitive element in both assemblies, with 97 597 copies in Darmor*‐bzh* and 92 747 copies in Tapidor (Table S2).

### Gene‐level comparison between Darmor*‐bzh* and Tapidor

Both genome assemblies were annotated using identical publicly available gene, EST, RNA‐Seq and protein data. Genes shorter than 100 bp, with an AED score of 1 (no evidence support for the annotation), or carrying transposase‐related PFAM domains were removed.

The resulting AUGUSTUS high confidence gene sets contained 80 382 predicted genes for Darmor*‐bzh* (37 220 on the A and 39 334 on the C pseudomolecules, 3228 on unplaced contigs), and 70 162 predicted genes for Tapidor (31 969 on the A and 37 562 on C pseudomolecules, 631 on unplaced contigs) (Table 1).

We compared the gene content between assemblies across each pair of homologous chromosomes. In Darmor*‐bzh*, 76 968 of 77 154 genes (99.7%) located on pseudomolecules had sequence identity with Tapidor genes. Of these, 65 280 were located in collinear blocks of at least five genes in the respective Tapidor pseudomolecule. Of the 11 688 genes with no partners in collinear blocks 2458 matched sequence on the expected chromosome, of which 701 matched the expected chromosomal region. In Tapidor, 69 372 of 69 531 genes (99.7%) showed sequence identity with Darmor*‐bzh* genes, with 59 099 genes in collinear blocks of at least five genes. Of the 10 903 genes with no partners in collinear blocks, 2103 matched with the expected chromosome, of which 524 matched within the expected range.

To further assess the differences in predicted gene content, we aligned sequence reads from both cultivars to each of the assemblies. Based on genomic read mapping, three Tapidor genes were predicted to be absent in Darmor*‐bzh*, while 73 Darmor*‐bzh* genes were predicted to be absent in Tapidor (Table 1). The genes predicted to be absent were compared with Darmor*‐bzh*, Tapidor, Ningyou‐7 and Tapidor x Ningyou‐7 RNA‐Seq data. Of the 73 genes, 21 (29%) showed no expression in Darmor‐*bzh* or Tapidor, 19 (26%) were expressed in Darmor‐*bzh* but neither Tapidor nor Ningyou‐7, 27 (37%) were expressed in Darmor‐*bzh* and Ningyou‐7 but not in Tapidor, and expression segregated in the Tapidor × Ningyou‐7 DH population, 2 (2.7%) were expressed in Tapidor and Ningyou‐7 but not in Darmor‐*bzh*, while 4 (5.5%) were expressed in all tissues in cases (Table S3). The three genes predicted to be absent in Darmor‐*bzh* but not in Tapidor were not expressed in Darmor‐*bzh*, Ningyou‐7 or Tapidor (Table S4).

Proteins encoded by genes predicted to be absent in one of the two cultivars were compared with Swiss‐Prot and checked for enriched GO terms. In genes present in Darmor*‐bzh* but not in Tapidor, terms such as ‘RNA splicing’ and ‘floral meristem growth’ were enriched (*P* < 0.05, Table S5). The three genes predicted to be present in Tapidor but not in Darmor*‐bzh* had no Swiss‐Prot hits, and therefore, transfer of GO annotation was not possible.

### Assessment of repetitive and collapsed assembly regions

**Figure 1:**
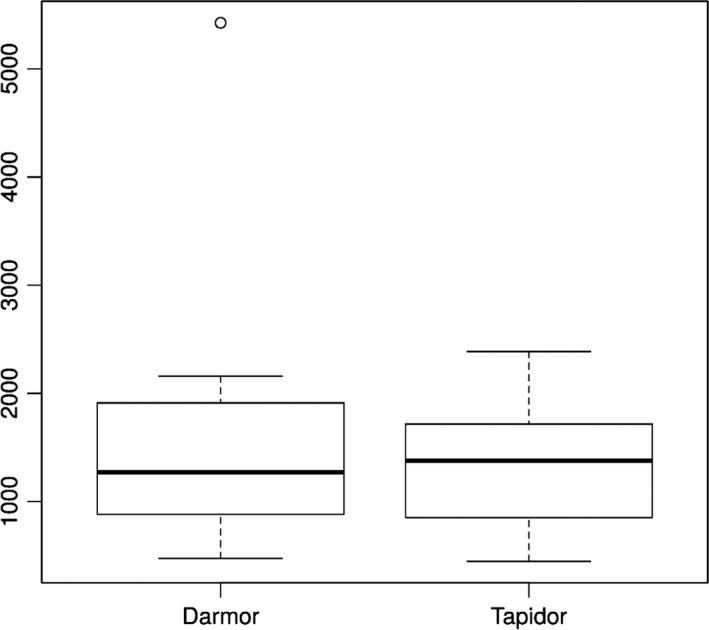
Comparison of the count of collapsed regions by chromosome in Darmor*‐bzh* and Tapidor.

**Figure 2:**
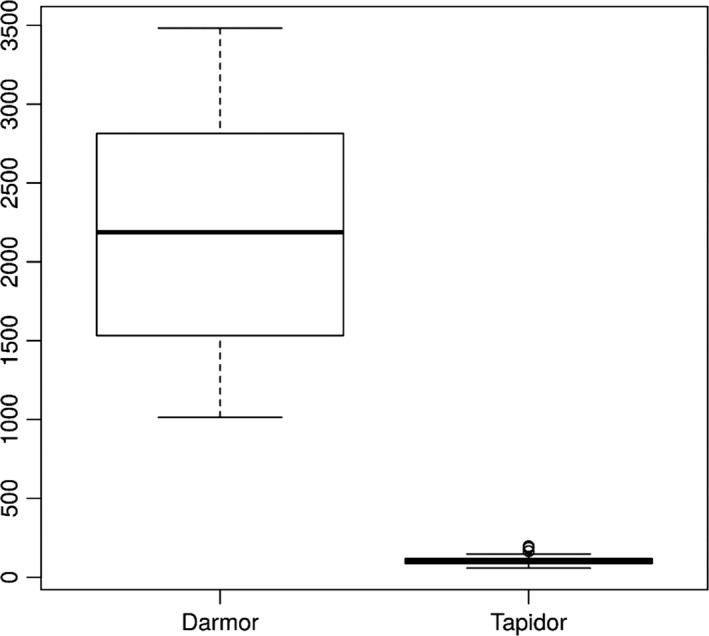
Comparison of the count of repetitive regions by chromosome in Darmor*‐bzh* and Tapidor.

Using the differential comparative read mapping pipeline CoReFinder, we identified 30 200 and 26 812 collapsed regions longer than 50 bp, with a total length of 12 495 844 bp and 10 522 089 bp and average sizes of 413 bp and 392 bp in the Darmor*‐bzh* and Tapidor assemblies, respectively (Figure 1, Table S6). We also identified 43 775 and 2191 repetitive regions, where the sequence is represented more than once in the assembly, totalling 31 807 543 bp and 972 605 bp, with an average of 720 bp and 437 bp in Darmor*‐bzh* and Tapidor, respectively (Figure 2, Table S7).

Genes were identified in both the collapsed and repetitive regions of the Darmor*‐bzh* and Tapidor assemblies. In Darmor*‐bzh*, 2455 genes (3.1%) were located in collapsed regions and 5703 (7.1%) were located in repetitive regions, while in Tapidor, 2651 genes (3.8%) were located in collapsed regions and 246 (0.4%) were located in repetitive regions (Table S8). In Tapidor, chromosomes A2 and C2 carried the largest number of collapsed genes (410, 15% and 245, 9.2%) while in Darmor*‐bzh*, chromosomes A3 and C3 carried the largest number of collapsed genes (212, 8.6% and 222, 9.0%). In Darmor*‐bzh*, the chromosomes A3 and C3 carried the largest number of repetitive genes (588, 10.3% and 470, 8.2%) while chromosomes A1 and C1 carried the most repetitive genes in Tapidor (26, 10.5% and 28, 11.4%).

**Figure 3:**
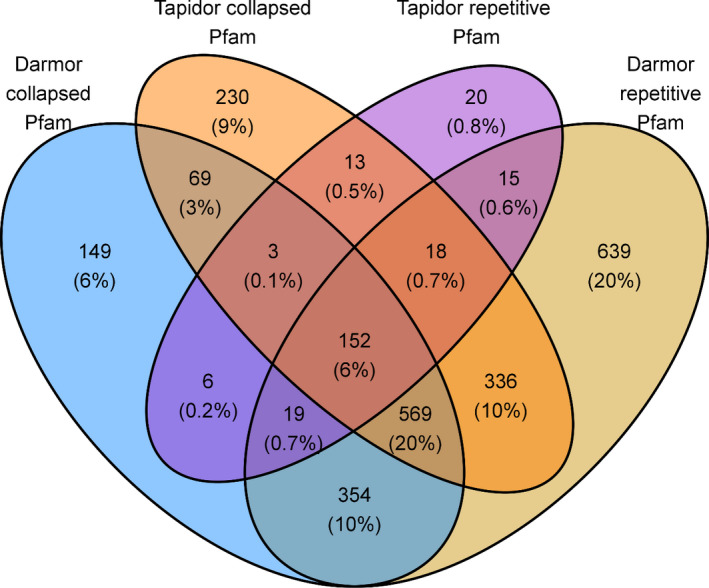
Number of shared Pfam domains between genes located in collapsed and repetitive regions between Darmor*‐bzh* and Tapidor.

Pfam protein domains and UniProtKB/Swiss‐Prot hits were identified for genes located in repetitive and collapsed regions in both assemblies. Both sets of results were compared between Darmor*‐bzh* and Tapidor in order to identify any common patterns that may lead to problems in genome assemblies. The largest number of shared Pfam domains and shared Swiss‐Prot hits was between genes located in repetitive regions in Darmor*‐bzh* and genes located in collapsed regions in Tapidor. In repetitive genes in Darmor*‐bzh*, 10% of Pfam domains overlapped with collapsed genes in Tapidor and Pfam domains collapsed in Darmor*‐bzh*, while only 0.5% of genes located in collapsed and repetitive regions in Tapidor shared Pfam domains (Figure 3).

The repetitive genes in Darmor*‐bzh* and collapsed genes in Tapidor contained repetitive domains such as ‘Myb‐like DNA‐binding domain’ or ‘pentatricopeptide repeat’ (PPRs) (Table S9). A similar pattern was exhibited in shared Swiss‐Prot hits where the greatest overlap in terms of Swiss‐Prot hits was between the set of proteins encoded by Darmor*‐bzh* genes located in collapsed regions and Tapidor genes located in repetitive regions (Figure S3). As with the Pfam domain overlaps, 10% of Swiss‐Prot hits were shared between repetitive genes in Darmor*‐bzh* and collapsed genes in Tapidor. The Swiss‐Prot hits contained proteins such as ‘Myosin‐6’ or ‘Proline‐rich receptor‐like protein kinase’ (Table S10).

## Discussion

*Brassica napus* canola is an important global crop and a major source of vegetable oil for human consumption. While genetic markers have been used for crop improvement for several years (Hayward *et al*., 2012), the application of genomics is only now leading to improved traits in the field. A major limitation of genomics based crop improvement is the lack of available reference genome assemblies. The first public *Brassica napus de novo* whole genome assembly was published in 2014, supported by a large multinational endeavour (Chalhoub *et al*., 2014). Since this achievement, DNA sequencing and bioinformatics technologies have advanced, and it has become increasingly accepted from studies in a wide range of crop species that a single reference is insufficient to describe the gene content of a species. To start to address this issue, we have produced an improved genome assembly for Darmor*‐bzh* and produced a second *de novo* whole genome assembly for a closely related cultivar, *B. napus* Tapidor. Tapidor and Darmor‐*bzh* both share Jet Neuf as an ancestor, but Darmor‐*bzh* additionally has the cultivar Bronowski in its direct ancestry (Foisset *et al*., 1995), while Tapidor contains Bienvenu in its ancestry (Sharpe and Lydiate, 2003).

To permit a direct comparison between the Tapidor and Darmor‐*bzh* genome assemblies, we improved the published Darmor*‐bzh* genome using high‐resolution skim genotyping by sequencing (Bayer *et al*., 2015) to position previously unplaced contigs within pseudomolecules. Both assemblies were then annotated using the same data and methods to prevent confounding effects of different annotation pipelines.

The assembly of the cultivar Tapidor was smaller (635 Mbp) compared with an assembly size of 850 Mbp for Darmor*‐bzh* (Table 1). Using k‐mers to predict both the assembly size and the genome size, we found that the estimated genome size was similar for both Darmor*‐bzh* (1345 Mbp) and Tapidor (1335 Mbp). In contrast, the predicted assembly size was similar to, although smaller than the actual assembly size with an estimated 808 Mbp for Darmor*‐bzh* and 697 Mbp for Tapidor. The predicted genome size estimate accounts for coverage, sequencing depth and unique k‐mers, while the genome assembly estimates account only for unique k‐mers. The Tapidor sequence data contains fewer unique k‐mers than the Darmor*‐bzh* sequencing data, which suggests that the Tapidor genome is actually smaller than the Darmor*‐bzh* genome. Both estimated genome sizes here are larger than the flow‐cytometry based estimations for different *B. napus* (AACC) cultivars of 1129–1235 Mbp (Arumuganathan and Earle, 1991). While the predicted genome size difference probably reflects a real difference in genome size, the discrepancy between the assembly size of Tapidor and Darmor*‐bzh* is probably due to the different sequencing technologies that were used for assembly. Tapidor was assembled with short paired end sequences, while Darmor*‐bzh* was assembled using a combination of paired end, 454 and Sanger sequencing (Chalhoub *et al*., 2014). The consistent differences between the assembly sizes and predicted genome sizes are common for genome assemblies and reflect the challenges of assembling the most repetitive regions of the genome.

While there appears to be a real difference in the genome size of these two homozygous individuals, we hypothesize that the different assembly methods cause the majority of observed differences between the assemblies. The CEGMA results suggest that both assemblies contain the majority of the gene content and that both assemblies are of high quality. Both assemblies contain all core eukaryotic genes (CEGs), although two CEGs were only partially present in Tapidor, which suggests that this assembly may be more fragmented. Similarly, the BUSCO results show that both assemblies carry the majority of gene content, with the Darmor*‐bzh* assembly having more gene copies than Tapidor. This suggests that some gene copies have collapsed in the Tapidor assembly which have not collapsed in the Darmor*‐bzh* assembly. The increased collapsing of regions in the Tapidor assembly compared to the Darmor*‐bzh* assembly is probably due to the use of long read 454 and Sanger sequence data in the production of the Darmor*‐bzh* assembly.

Both the genomes were annotated using the same methods and supporting data. Gene prediction is dependent on a wide range of factors and the number of predicted genes is influenced by the stringency of gene calling. To enable the most robust comparison of predicted genes between these assemblies, we applied a relatively strict gene calling approach. The number of predicted genes for Darmor*‐bzh* is lower in our study than the 101 040 gene models originally predicted by Chalhoub *et al*. (2014), and this difference is likely due to the more stringent methods of repeat masking employed, including the removal of transposon‐related and short genes.

As the Tapidor contigs were scaffolded based on the Darmor*‐bzh* assembly, and both Tapidor and Darmor‐*bzh* have shared ancestry, we expected to see extensive collinearity between the genome assemblies. We assessed this by comparison of genes present in collinear blocks along the pseudomolecules. We also examined whether genes which were predicted to be missing from collinear blocks were in fact missing or just remained unannotated. In Darmor*‐bzh*, 11 688 genes (14.5%) and in Tapidor 10 903 genes (15.5%) were not in collinear blocks and were not identified in their expected regions, suggesting that they are either misplaced in the assembly or are nonsyntenic in the genome. Wicker *et al*. (2011) estimated that 17% of Triticeae genes are nonsyntenic with model grasses, which is similar to our findings in *B. napus*. Extensive homeologous recombination has been observed in *B. napus* (Cai *et al*., 2014; Chalhoub *et al*., 2014; Nicolas *et al*., 2007) which may also explain some of the nonsyntenic regions observed here.

As differences in genome assembly and annotation may suggest differences in gene content which are not reflected in the actual genome, we investigated the number of genes predicted to be absent in each cultivar based on raw data and independent of gene assembly or annotation (Table 1). Mapping genomic sequence reads between references suggests that 73 genes are unique to Darmor*‐bzh* and absent in Tapidor, while three genes are unique to Tapidor and absent in Darmor*‐bzh*. Of the 73 genes predicted to be absent, 27 (37%) were expressed in Darmor‐*bzh* and Ningyou‐7 but not in Tapidor, with clear segregation in the Tapidor × Ningyou‐7 DH population, suggesting that these genes have been truly lost in Tapidor but are present in Ningyou‐7 (Table S3). The 19 (26%) genes expressed in Darmor‐*bzh* but not in Tapidor or Ningyou‐7 may also be absent in Ningyou‐7, or their expression may not be captured in the Darmor‐*bzh* RNA‐Seq libraries. Some error remains, as two genes (2.7%) predicted to be absent in Tapidor and present in Darmor‐*bzh* appear to be expressed in Tapidor but not in Darmor‐*bzh,* and four genes (5.5%) appear to be expressed in all cultivars. These results may be due to the more relaxed mapping parameters for the single RNASeq reads compared to the paired genomic sequence reads, leading to erroneous read mapping and false calling of gene presence in the RNASeq analysis. Genes which showed no expression in any of the cultivars assessed may be pseudogenes, misannotated or only expressed in certain conditions or tissues which were not sampled during RNA‐Seq library preparation (Table S4).

The relatively small difference in the presence of unique genes compares with 10 220 annotated Darmor*‐bzh* genes which are not found in the Tapidor assembly. To test whether this difference is due to duplicate gene copies that have collapsed into single copies in the Tapidor assembly, we investigated the number and size of collapsed and repetitive regions in both assemblies (Table S6, Table S7, Figures 1 and 2). The number and the size of repetitive regions were greater in the Darmor*‐bzh* assembly by a factor of 19 and 32, respectively. These numbers are in line with the above gene number differences as well as the differences of assembly size and estimated assembly size, and suggests that the longer 454 and Sanger reads used in the Darmor*‐bzh* assembly lead to more repetitive regions being assembled and less collapsing of genomic regions in Darmor*‐bzh*.

Homeologous exchanges (HEs) are frequently observed in polyploid species, and the number of genes located in repetitive and collapsed regions can be used for the detection of HE between the A and the C genome (Chalhoub *et al*., 2014). Here, the largest and second largest number of collapsed and repetitive genes in both assemblies is always between a chromosome and its homeologous partner chromosome in the same cultivar, such as A2 and C2 for the number of collapsed genes in Darmor‐*bzh*. The pseudomolecule with the greatest size of collapsed region is C2 in Darmor‐*bzh* which is also consistent with prior reports (Chalhoub *et al*., 2014). When comparing both cultivars, chromosome C2 shows the greatest difference, with 23 (31%) of genes not present in Tapidor in the first 10 Mbp, and 245 (9.2%) of collapsed genes in Tapidor being located on C2 in the first 10 Mbp. The number of collapsed genes suggests homeologous exchange of the first 10 Mbp between A2 and C2 with subsequent loss of the original C2 arm in Tapidor (a homeologous nonreciprocal translocation, HNRT), and that this genome structure is not present in Darmor‐*bzh*. The genes in this region are enriched for the GO‐term ‘floral meristem growth’ (*P* < 0.05), and chromosome C2 contains several breeding related QTLs such as seed yield, seed weight and heterosis (Zhao *et al*., 2016). This suggests that selection for agronomic traits may be responsible for the observed differences between Darmor*‐bzh* and Tapidor on this chromosome.

The inability to assemble repetitive sequences can lead to the underestimation of gene content and the incorrect calling of presence/absence variation between assemblies. The annotation of genes located in repetitive and collapsed regions helps to identify which protein domains may be associated with poor assembly and subject to incorrectly called the presence/absence variation. We observed an overlap in shared domains and Swiss‐Prot hits between genes located in repetitive regions in Darmor*‐bzh* and collapsed regions in Tapidor (Figure 3, Figure S3). Shared domains include known repetitive domains such as WD repeats or pentatricopeptide repeats (PPRs), while shared Swiss‐Prot hits included leucine‐rich repeat receptor kinases, as well as somatic embryogenesis receptor kinases which contain leucine repeats (Hecht *et al*., 2001). This suggests that genes which have repetitive domains under‐assemble using only short‐read technology but at least some of these may be correctly assembled when longer sequence reads are included in the assembly process. However, the inclusion of 454 reads does not correctly assemble all repetitive genes, as many collapse‐related Pfam and Swiss‐Prot domains are located in collapsed regions in Darmor*‐bzh*, indicating that there is still missing gene content in the high quality Darmor*‐bzh* assembly (Figure 3). The potential collapsing of genomic regions containing important agronomic genes is important for both future genome assembly projects and future pangenome analysis as the capturing of all gene content which may play a role in agronomic performance is important for the application of genomics assisted breeding.

The differential assembly of genes with repetitive domains along with the presence of homeologous exchanges is likely to confound the assessment of gene presence variation between individuals and the translation of this information for the agronomic improvement of crop species. For example, there are several reports of variation in NBS LRR gene content between individuals and that this may play an important role in the resistance or susceptibility of crops to disease (Tollenaere *et al*., 2012; Wu *et al*., 2014). While the evidence suggests that there is likely to be some true variation in the presence of these genes, it is important to differentiate between true variation and assembly or annotation issues when interpreting the biological function of these genes as assembly and annotation variation can confound gene trait associations.

## Conclusions

We have *de novo* assembled and annotated a second genome for the important oilseed crop species, *B. napus* canola, and compared this with an improved and reannotated assembly of the published cultivar Darmor*‐bzh* (Chalhoub *et al*., 2014). We demonstrate that comparison of gene content between individuals needs to account for differences in assembly and annotation to avoid misinterpretation. Between the two assemblies, only three and 73 predicted genes represent real gene loss between these closely related cultivars, 524 and 701 (0.7%–0.8%) are due to misannotation (i.e. the gene annotation process failed to identify a gene in one assembly but predicted its presence in the other assembly), while 10 903 and 11 688 (14%–15%) of the differences were due to either misplaced contigs or real gene movement. Our results suggest that researchers comparing different genome assemblies should not rely solely on the results of annotation pipelines but should also compare the assemblies and the unassembled read data to differentiate between real differences and artefacts. In particular, genes with repetitive domains may collapse leading to an underestimation of gene copy number. By accounting for these potential errors, it is possible for future studies to establish an accurate pangenome for this important oilseed crop.

## Methods

### Darmor*‐bzh* and Tapidor genome assemblies

All Tapidor reads were cleaned using sickle (Joshi and Fass, 2011) and normalized using khmer v1.0 normalize_by_median.py (Brown *et al*., 2012; Crusoe *et al*., 2015). VelvetOptimiser (Gladman and Seemann, 2012) was used with Velvet (Zerbino and Birney, 2008) to assemble contigs. Contigs greater than 1 Kbp were sorted by comparison with the Darmor*‐bzh* assembly using blastn (Altschul *et al*., 1990) with an e‐value of 1e‐6. Contigs with two highest‐scoring alignments within 1% were unplaced. Contigs were then sorted using LASTZ (Harris, 2007) and a custom script (LASTZSorter.py, available at http://appliedbioinformatics.com.au/index.php/Darmor_Tapidor). MSTMap (Wu *et al*., 2008) was used to calculate the genetic map using the unimputed SNPs of the DH population (distance_function: kosambi, cut_off_p_value: 0.0000001, no_map_size 2, missing_threshold 0.1).

SGSautoSNP (Lorenc *et al*., 2012) was used with the SkimGBS pipeline (Bayer *et al*., 2015) to call SNPs and genotypes using either the Tapidor or Darmor*‐bzh* genome as reference (Chalhoub *et al*., 2014). ContigPlacer was used to place unplaced contigs in the Darmor*‐bzh* and Tapidor assemblies and is available at https://github.com/philippbayer/contigPlacer.

### Genome annotation

RepeatModeler v1.0.8 and RepeatMasker v4.0.6 (http://repeatmasker.org) together with Repbase v were used to mask repeats. Gene models were produced using Tophat v2.1.0 and cufflinks v2.2.0 (Kim *et al*., 2013), and annotation was performed using MAKER v2.31 (Cantarel *et al*., 2008). RNA‐Seq data for the cultivars Tapidor, Ningyou and TN DH (Higgins *et al*., 2012) were used together with Darmor*‐bzh* RNA‐Seq reads (Chalhoub *et al*., 2014).

CEGMA v2.5 (Parra *et al*., 2007) was used to annotate the *Brassica oleracea* C genome (Parkin *et al*., 2014) with core gene models (COGs). The annotated core genes were used as input for AUGUSTUS v3.0.2 (Keller *et al*., 2011). A set of ESTs from the 95k microarray (Trick *et al*., 2009), Brassicaceae proteins from RefSeq, and *B. rapa*,* B. oleracea* and *B. napus* unigenes from UniGene (NCBI Resource Coordinators 2013) were used as evidence. Predicted genes with an AED‐score of 1, shorter than 100 bp, or carrying transposase‐related domains were removed from subsequent analysis. Both assemblies were assessed using CEGMA v2.5 (Parra *et al*., 2007) and BUSCO v1.1.b1 (Simão *et al*., 2015) with the early release plants dataset. Predicted transcripts and proteins were renamed according to the standardized *Brassica* nomenclature (Østergaard *et al*., 2008).

### Gene‐level comparison between Darmor*‐bzh* and Tapidor

MCScanX (Wang *et al*., 2012) was used to analyse collinearity between the Darmor*‐bzh* and Tapidor genes and to assign predicted genes to blocks of at least five collinear genes. Custom scripts were used to parse the MCScanX output. Genes were compared with the Tapidor pseudomolecules using blastp with an e‐value cut‐off of 1e‐10. The scripts are available at http://appliedbioinformatics.com.au/index.php/Darmor_Tapidor under ‘Colinearity analysis’.

Assessment of gene loss used the SGSGeneLoss pipeline v0.1 (Golicz *et al*., 2014). Public Darmor*‐bzh* (BioProject ID: ERP005275, ERP005532) reads were aligned using bowtie2 (settings: –end‐to‐end, –sensitive) (Langmead and Salzberg, 2012) and extracted using samtools (Li *et al*., 2009). Gene expression levels of the genes predicted to be absent were measured using bowtie v1.1.2 (Langmead *et al*., 2009) and eXpress v1.5.1 (Roberts and Pachter, 2012) and the same RNA‐Seq data as used for the annotation. Predicted genes showing less than five unique fragments mapping in a library were assumed to be not expressed in that library to account for possible mismappings from expressed homeologous genes.

Genes were functionally annotated using blastp and UniProtKB Swiss‐Prot. GO terms were determined from Swiss‐Prot results and topGO as used to predict enriched GO‐terms (Alexa and Rahnenfuhrer, 2010).

To identify collapsed and repeated regions in the Tapidor and Darmor*‐bzh* assemblies, reads from each cultivar were mapped to their respective genome reference assemblies using SOAPaligner v2.21 with parameters ‐r 0, ‐r 1 and ‐r 2 to generate three sets of alignments. Base coverage was calculated for each BAM file using BEDTools genomecov v2.21.0 (Quinlan and Hall, 2010). The CoReFinder pipeline (http://appliedbioinformatics.com.au/index.php/CoReFinder) identified collapsed and repetitive regions with a minimum block size of 50 bp. R v3.2 (R Development Core Team 2011) was used to perform the Mann–Whitney U‐test using the wilcox.test() function to compare the number and lengths of repetitive and collapsed regions between Darmor*‐bzh* and Tapidor. Conserved domains were identified using the command‐line version of InterProScan version 5.14‐53.0 (Zdobnov and Apweiler, 2001) (settings: ‐appl Pfam).

### Data access

All raw sequence reads for the cultivar Tapidor used for assembly have been submitted to NCBI BioProject (https://www.ncbi.nlm.nih.gov/bioproject/342383). The Tapidor × Ningyou‐7 DH data are available at http://www.ncbi.nlm.nih.gov/bioproject/PRJNA274890

The assemblies and annotations along with scripts and other information described in this manuscript are available at http://appliedbioinformatics.com.au/index.php/Darmor_Tapidor.

## Disclosure declaration

The authors declare no competing interests.

## Supporting information


**Figure S1** Dotplot comparison of the Darmor‐*bzh* assembly (*y*‐axis) and the Tapidor assembly (*x*‐axis) pseudomolecules.

**Figure S2** Comparison of ranks between 19 linkage maps from MSTMap and physical placement using the Darmor‐*bzh* genome assembly as reference.

**Figure S3** Number of shared Swiss‐Prot hits between genes located in collapsed and repetitive regions between Darmor‐*bzh* and Tapidor.

**Table S1** Overlap between collections of previously unplaced contigs and contigs placed in the improved Darmor assembly.
**Table S2** Repeats in Darmor and Tapidor.
**Table S3** Gene expression values for 73 genes present in Darmor and predicted to be absent in Tapidor using RNA‐Seq data from Darmor, Tapidor, Ningyou‐7 and the Tapidor x Ningyou‐7 population.
**Table S4** Gene expression values for 3 genes present in Tapidor and predicted to be absent in Darmor using RNA‐Seq data from Darmor, Tapidor, Ningyou‐7 and the Tapidor x Ningyou‐7 population.
**Table S5** TopGO enriched terms for genes absent in Tapidor but present in Darmor.
**Table S6** Number and total sizes of collapsed regions in Darmor and Tapidor.
**Table S7** Number and total sizes of repetitive regions in Darmor and Tapidor.
**Table S8** Number of genes in predicted repetitive and collapsed regions in Darmor and Tapidor.
**Table S9** Shared domains between Darmor genes located in repetitive regions and Tapidor genes located in collapsed regions (only ten most common domains).
**Table S10** Ten most common Swiss‐Prot hits for repetitive genes in Darmor and collapsed genes in Tapidor with an e‐value cutoff of 1e‐5.
