# Supplementary figures and images for: Assembly and comparison of two closely related Brassica napus genomes
Source: Plant Biotechnol J. 2017 Jun 14;15(12):1602–10. doi: 10.1111/pbi.12742 (PMC5698052; doi:10.1111/pbi.12742)

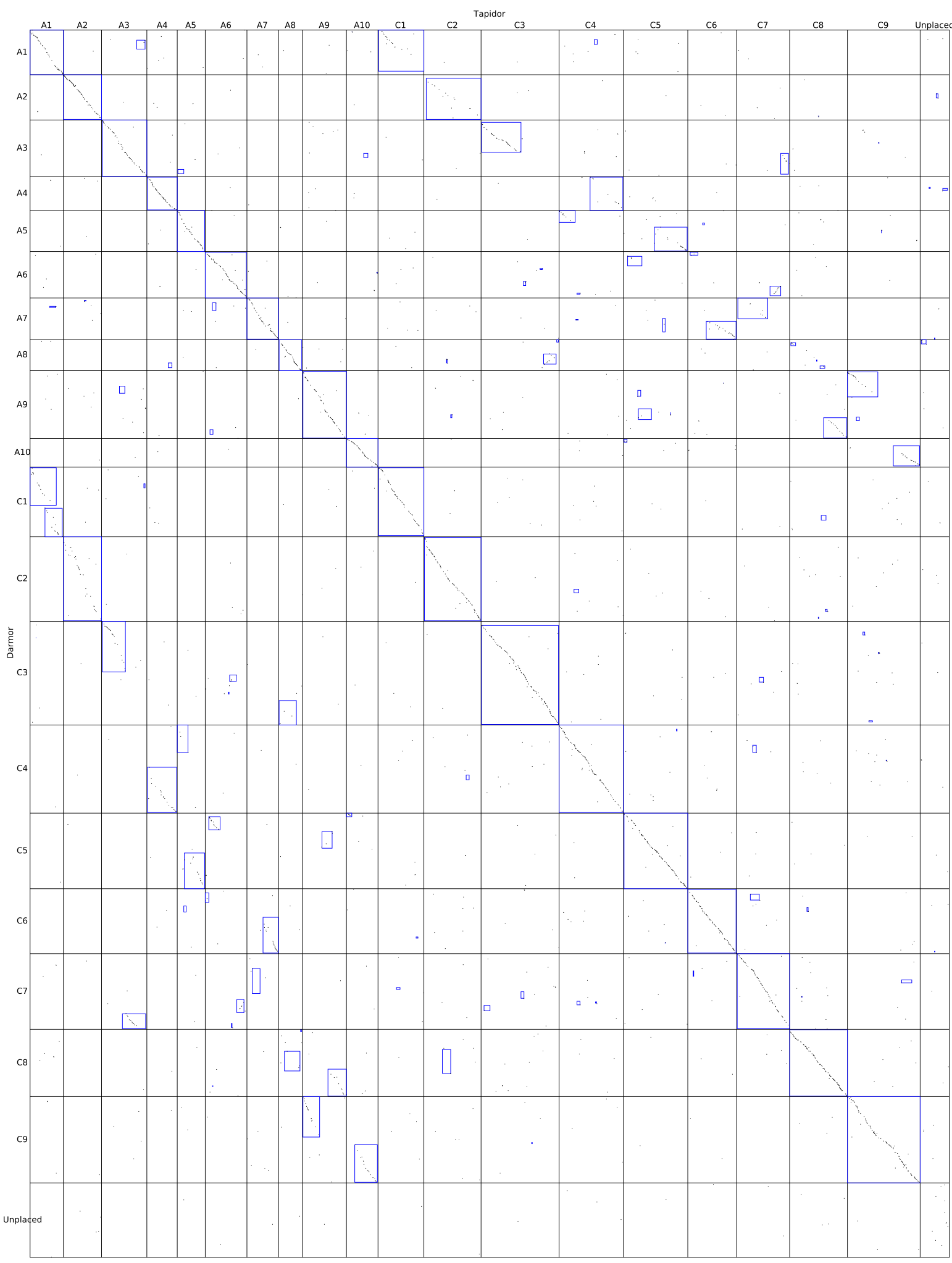

Supplement: Supplementary file 1 — Figure S1 Dotplot comparison of the Darmor‐bzh assembly (y‐axis) and the Tapidor assembly (x‐axis) pseudomolecules. [file PBI-15-1602-s004.docx]

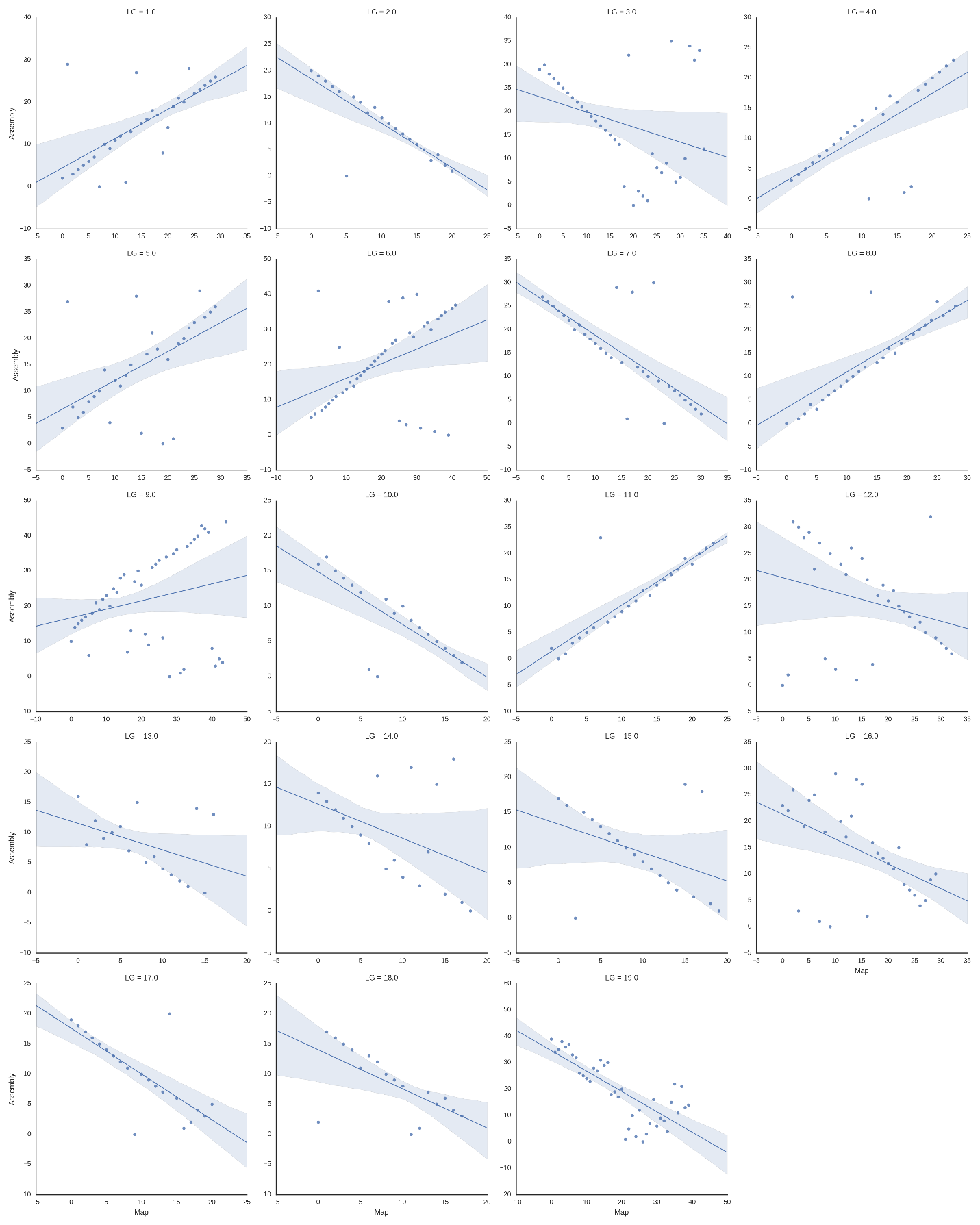

Supplement: Supplementary file 2 — Figure S2 Comparison of ranks between 19 linkage maps from MSTMap and physical placement using the Darmor‐bzh genome assembly as reference. [file PBI-15-1602-s003.docx]

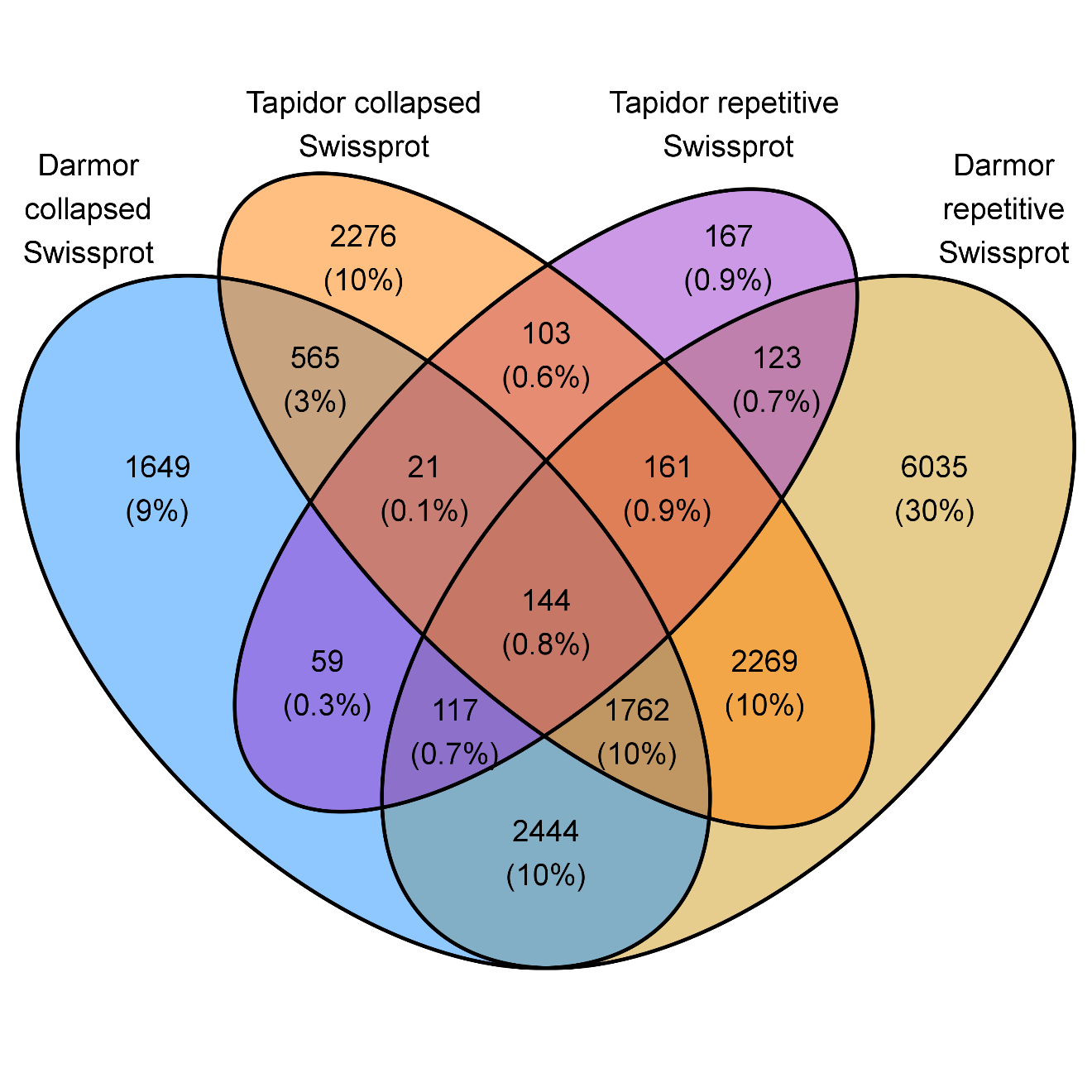

Supplement: Supplementary file 3 — Figure S3 Number of shared Swiss‐Prot hits between genes located in collapsed and repetitive regions between Darmor‐bzh and Tapidor. [file PBI-15-1602-s002.docx]
