# Supplementary material for: Assembly and comparison of two closely related Brassica napus genomes
Source: Plant Biotechnol J. 2017 Jun 14;15(12):1602–10. doi: 10.1111/pbi.12742 (PMC5698052; doi:10.1111/pbi.12742)
Supplement: Supplementary file 4 — Table S1 Overlap between collections of previously unplaced contigs and contigs placed in the improved Darmor assembly. Table S2 Repeats in Darmor and Tapidor. Table S3 Gene expression values for 73 genes present in Darmor and predicted to be absent in Tapidor using RNA‐Seq data from Darmor, Tapidor, Ningyou‐7 and the Tapidor x Ningyou‐7 population. Table S4 Gene expression values for 3 genes present in Tapidor and predicted to be absent in Darmor using RNA‐Seq data from Darmor, Tapidor, Ningyou‐7 and the Tapidor x Ningyou‐7 population. Table S5 TopGO enriched terms for genes absent in Tapidor but present in Darmor. Table S6 Number and total sizes of collapsed regions in Darmor and Tapidor. Table S7 Number and total sizes of repetitive regions in Darmor and Tapidor. Table S8 Number of genes in predicted repetitive and collapsed regions in Darmor and Tapidor. Table S9 Shared domains between Darmor genes located in repetitive regions and Tapidor genes located in collapsed regions (only ten most common domains). Table S10 Ten most common Swiss‐Prot hits for repetitive genes in Darmor and collapsed genes in Tapidor with an e‐value cutoff of 1e‐5. [file PBI-15-1602-s001.pdf]

Supplementary Table 1: Overlap between collections of previously unplaced contigs and contigs placed in the improved Darmor assembly

Collection of unplaced contigs	Contigs placed on same chromosome by contigPlacer	Previously unplaced contigs	Percentage overlap
chrA01	101	101	1
chrA02	57	58	0.983
chrA03	116	117	0.992
chrA04	75	77	0.974
chrA05	92	99	0.929
chrA06	110	111	0.991
chrA07	88	90	0.978
chrA08	49	51	0.961
chrA09	126	128	0.984
chrA10	74	75	0.987
chrC01	155	159	0.975
chrC02	149	153	0.974
chrC03	146	151	0.967
chrC04	149	150	0.993
chrC05	95	97	0.979
chrC06	105	105	1
chrC07	71	72	0.986
chrC08	127	129	0.984
chrC09	139	140	0.993

Supplementary Table 2: Repeats in Darmor and Tapidor

	Darmor			Tapidor		
	Number of elements	Length (bp)	Percentage	Number of elements	Length (bp)	Percentage
SINEs:	13,830	2,268,190	0.27%	13,747	2,250,366	0.35%
LINEs:	36,548	27,166,176	3.19%	39,123	24,143,511	3.79%
LINE1	35,766	26,761,196	3.15%	36,227	23,159,907	3.64%
LTR elements:	124,318	115,347,362	13.57%	115,389	69,213,440	10.88%
ERV_classII	265	155,291	0.02%	92	46,791	0.01%
DNA elements:	165,572	65,516,456	7.71%	148,658	41,426,419	6.51%
hAT-Charlie	957	199,738	0.02%	2,535	815,769	0.13%
Unclassified:	267,271	88,073,813	10.36%	283,158	77,334,410	12.15%
Total interspersed repeats		298,371,997	35.09%		214,368,146	33.69%
Small RNA:	10,873	2,670,218	0.31%	11,619	2,010,379	0.32%
Satellites:	1,911	818,211	0.1%	3,289	758,232	0.12%
Simple repeats:	171,999	8,512,400	1%	150,456	6,905,421	1.09%
Low complexity:	38,370	1,946,233	0.23%	33,853	1,693,601	0.27%

Supplementary Table 3: Gene expression values for 73 genes present in Darmor and predicted to be absent in Tapidor using RNA-Seq data from Darmor, T

Gene	Library	Unique Counts	FPKM	FPKM Confidence low
BnaA10g04830.1D2	Darmor_AUP_AOSW_2_D09BTACXX.I	9	0.271828	0.271828
	Darmor_AUP_BOSW_3_D09BTACXX.I	15	0.6124845	0.6124845
	Darmor_AUP_DOSW_2_D09BTACXX.I	0	0	0
	Darmor_AUP_FOSW_4_D09BTACXX.II	0	0	0
	Darmor_AUP_HOSW_5_D09BTACXX.I	7	0.2871137	0.2871137
	Darmor_AUP_IAOSW_1_1_C0UW4AC	29	0.3935434	0.3935434
	Darmor_AUP_KAOSW_2_1_C0UW4A	61	0.8264428	0.8264428
	Darmor_AUP_LAOSW_3_1_C0UW4AC	0	0	0
	Darmor_AUP_MAOSW_4_1_C0UW4A	0	0	0
	Darmor_AUP_NAOSW_5_1_C0UW4A	0	0	0
	Darmor_AUP_PAOSW_6_1_C0UW4AC	185	2.561089	2.561089
	Ningyou_ERR037335	0	7.38E-37	0
	TN04_ERR037297	0	0	0
	TN05_ERR037298	0	0	0
	TN08_ERR037301	0	0	0
	TN103_ERR037317	0	0	0
	TN108_ERR037318	0	0	0
	TN109_ERR037319	0	0	0
	TN10_ERR037294	0	0	0
	TN121_ERR037320	0	0	0
	TN124_ERR037321	0	0	0
	TN126_ERR037322	0	0	0
	TN128_ERR037323	0	0	0
	TN129_ERR037324	0	0	0
	TN12_ERR037302	0	0	0
	TN138_ERR037325	0	0	0
	TN149_ERR037326	0	0	0
	TN161_ERR037327	0	0	0
	TN163_ERR037328	0	0	0
	TN170_ERR037329	0	0	0
	TN171_ERR037332	0	0	0

	TN172_ERR037333	0	0	0
	TN176_ERR037330	0	0	0
	TN177_ERR037331	0	0	0
	TN24_ERR037299	0	0	0
	TN28_ERR037295	0	0	0
	TN30_ERR037305	0	0	0
	TN31_ERR037303	0	0	0
	TN38_ERR037304	0	0	0
	TN44_ERR037306	0	0	0
	TN48_ERR037307	0	0	0
	TN51_ERR037308	0	0	0
	TN57_ERR037309	0	0	0
	TN72_ERR037312	0	0	0
	TN76_ERR037311	0	0	0
	TN77_ERR037310	0	0	0
	TN85_ERR037315	0	0	0
	TN88_ERR037313	0	0	0
	TN90_ERR037316	0	0	0
	Tapidor_ERR037334	0	0	0
BnaC02g10670.1D2	Darmor_AUP_AOSW_2_D09BTACXX.I	168	4.067222	3.500619
	Darmor_AUP_BOSW_3_D09BTACXX.I	279	8.732537	8.056763
	Darmor_AUP_DOSW_2_D09BTACXX.I	205	5.00943	4.494313
	Darmor_AUP_FOSW_4_D09BTACXX.II	140	4.380874	3.900246
	Darmor_AUP_HOSW_5_D09BTACXX.I	106	3.844972	3.777374
	Darmor_AUP_IAOSW_1_1_C0UW4AC	454	4.973223	4.487683
	Darmor_AUP_KAOSW_2_1_C0UW4A	438	4.971961	4.70785
	Darmor_AUP_LAOSW_3_1_C0UW4AC	488	5.370184	5.05407
	Darmor_AUP_MAOSW_4_1_C0UW4A	496	5.652043	5.336384
	Darmor_AUP_NAOSW_5_1_C0UW4A	558	6.392542	5.997986
	Darmor_AUP_PAOSW_6_1_C0UW4AC	506	5.429244	5.354958
	Ningyou_ERR037335	1	0.04036543	0.04029376
	TN04_ERR037297	0	2.98E-27	0
	TN05_ERR037298	2	0.2041114	0.2040762

	TN08_ERR037301	0	6.34E-29	0
	TN103_ERR037317	2	0.09506797	0.09506794
	TN108_ERR037318	0	4.30E-28	0
	TN109_ERR037319	1	0.03735975	0.03596505
	TN10_ERR037294	0	2.14E-17	0
	TN121_ERR037320	0	4.43E-23	0
	TN124_ERR037321	1	0.03077969	0.02861889
	TN126_ERR037322	1	0.07195103	0.05611165
	TN128_ERR037323	0	1.06E-28	0
	TN129_ERR037324	0	1.53E-18	0
	TN12_ERR037302	1	0.03682674	0.03533539
	TN138_ERR037325	0	1.61E-28	0
	TN149_ERR037326	1	0.09795869	0
	TN161_ERR037327	1	0.05085907	0
	TN163_ERR037328	2	0.07432646	0.02804435
	TN170_ERR037329	2	0.06230237	0.06005022
	TN171_ERR037332	0	2.20E-18	0
	TN172_ERR037333	2	0.06236717	0.06199623
	TN176_ERR037330	0	3.79E-33	0
	TN177_ERR037331	0	2.58E-29	0
	TN24_ERR037299	1	0.04373916	0.03403113
	TN28_ERR037295	0	4.86E-26	0
	TN30_ERR037305	0	1.20E-13	0
	TN31_ERR037303	0	1.27E-25	0
	TN38_ERR037304	1	0.03639125	0.01520706
	TN44_ERR037306	0	5.20E-25	0
	TN48_ERR037307	1	0.03856766	0.03856685
	TN51_ERR037308	1	0.04422641	0.0332304
	TN57_ERR037309	1	0.03225864	0.018385
	TN72_ERR037312	0	2.04E-16	0
	TN76_ERR037311	1	0.03454714	0.03115492
	TN77_ERR037310	0	1.24E-28	0
	TN85_ERR037315	0	2.32E-26	0

	TN88_ERR037313	0	1.07E-28	0
	TN90_ERR037316	2	0.06843885	0.05689483
	Tapidor_ERR037334	0	5.68E-26	0
BnaC02g07340.1D2	Darmor_AUP_AOSW_2_D09BTACXX.I	0	0	0
	Darmor_AUP_BOSW_3_D09BTACXX.I	0	0	0
	Darmor_AUP_DOSW_2_D09BTACXX.I	0	0	0
	Darmor_AUP_FOSW_4_D09BTACXX.I	0	0	0
	Darmor_AUP_HOSW_5_D09BTACXX.I	0	0	0
	Darmor_AUP_IAOSW_1_1_C0UW4A	0	0	0
	Darmor_AUP_KAOSW_2_1_C0UW4A	0	6.43E-44	0
	Darmor_AUP_LAOSW_3_1_C0UW4A	0	0	0
	Darmor_AUP_MAOSW_4_1_C0UW4	0	0	0
	Darmor_AUP_NAOSW_5_1_C0UW4A	0	0	0
	Darmor_AUP_PAOSW_6_1_C0UW4A	0	0	0
	Ningyou_ERR037335	0	8.50E-29	0
	TN04_ERR037297	0	0	0
	TN05_ERR037298	0	0	0
	TN08_ERR037301	0	0	0
	TN103_ERR037317	0	0	0
	TN108_ERR037318	0	0	0
	TN109_ERR037319	0	0	0
	TN10_ERR037294	0	0	0
	TN121_ERR037320	0	0	0
	TN124_ERR037321	0	0	0
	TN126_ERR037322	0	0	0
	TN128_ERR037323	0	0	0
	TN129_ERR037324	0	8.34E-33	0
	TN12_ERR037302	0	0	0
	TN138_ERR037325	0	0	0
	TN149_ERR037326	0	0	0
	TN161_ERR037327	0	0	0
	TN163_ERR037328	0	0	0
	TN170_ERR037329	0	0	0

	TN171_ERR037332	0	0	0
	TN172_ERR037333	0	0	0
	TN176_ERR037330	0	0	0
	TN177_ERR037331	0	0	0
	TN24_ERR037299	0	0	0
	TN28_ERR037295	0	0	0
	TN30_ERR037305	0	0	0
	TN31_ERR037303	0	0	0
	TN38_ERR037304	0	0	0
	TN44_ERR037306	0	0	0
	TN48_ERR037307	0	0	0
	TN51_ERR037308	0	0	0
	TN57_ERR037309	0	0	0
	TN72_ERR037312	0	0	0
	TN76_ERR037311	0	0	0
	TN77_ERR037310	0	0	0
	TN85_ERR037315	0	0	0
	TN88_ERR037313	0	4.40E-39	0
	TN90_ERR037316	0	0	0
	Tapidor_ERR037334	0	6.28E-41	0
BnaU01g26060.1D2	Darmor_AUP_AOSW_2_D09BTACXX.I	0	0.06225868	0
	Darmor_AUP_BOSW_3_D09BTACXX.I	0	2.29E-34	0
	Darmor_AUP_DOSW_2_D09BTACXX.I	0	7.91E-38	0
	Darmor_AUP_FOSW_4_D09BTACXX.II	0	0	0
	Darmor_AUP_HOSW_5_D09BTACXX.I	0	0	0
	Darmor_AUP_IAOSW_1_1_C0UW4AC	0	0	0
	Darmor_AUP_KAOSW_2_1_C0UW4A	0	0	0
	Darmor_AUP_LAOSW_3_1_C0UW4A(	0	0	0
	Darmor_AUP_MAOSW_4_1_C0UW4/	0	0	0
	Darmor_AUP_NAOSW_5_1_C0UW4A	0	0	0
	Darmor_AUP_PAOSW_6_1_C0UW4A(	0	0.05810465	0
	Ningyou_ERR037335	0	0	0
	TN04_ERR037297	0	0	0

	TN05_ERR037298	0	0	0
	TN08_ERR037301	0	0	0
	TN103_ERR037317	0	0	0
	TN108_ERR037318	0	0	0
	TN109_ERR037319	0	0	0
	TN10_ERR037294	0	0	0
	TN121_ERR037320	0	0	0
	TN124_ERR037321	0	0	0
	TN126_ERR037322	0	0	0
	TN128_ERR037323	0	0	0
	TN129_ERR037324	0	0	0
	TN12_ERR037302	0	0	0
	TN138_ERR037325	0	0	0
	TN149_ERR037326	0	0	0
	TN161_ERR037327	0	0	0
	TN163_ERR037328	0	0	0
	TN170_ERR037329	0	0	0
	TN171_ERR037332	0	0	0
	TN172_ERR037333	0	0	0
	TN176_ERR037330	0	0	0
	TN177_ERR037331	0	1.23E-35	0
	TN24_ERR037299	0	3.20E-31	0
	TN28_ERR037295	0	4.03E-37	0
	TN30_ERR037305	0	3.93E-64	0
	TN31_ERR037303	0	0	0
	TN38_ERR037304	0	0	0
	TN44_ERR037306	0	0	0
	TN48_ERR037307	0	0	0
	TN51_ERR037308	0	0	0
	TN57_ERR037309	0	0	0
	TN72_ERR037312	0	1.89E-41	0
	TN76_ERR037311	0	0	0
	TN77_ERR037310	0	0	0

	TN85_ERR037315	0	2.67E-29	0
	TN88_ERR037313	0	0	0
	TN90_ERR037316	0	2.05E-41	0
	Tapidor_ERR037334	0	0	0
BnaC02g07350.1D2	Darmor_AUP_AOSW_2_D09BTACXX.I	4	0.1200806	0.1191515
	Darmor_AUP_BOSW_3_D09BTACXX.I	2	0.08221762	0.08220911
	Darmor_AUP_DOSW_2_D09BTACXX.I	0	4.13E-37	0
	Darmor_AUP_FOSW_4_D09BTACXX.II	0	0	0
	Darmor_AUP_HOSW_5_D09BTACXX.I	1	0.04206064	0.04206064
	Darmor_AUP_IAOSW_1_1_C0UW4AC	2	0.02813636	0.02813636
	Darmor_AUP_KAOSW_2_1_C0UW4A	3	0.0421691	0.0421691
	Darmor_AUP_LAOSW_3_1_C0UW4AC	0	0	0
	Darmor_AUP_MAOSW_4_1_C0UW4A	0	0	0
	Darmor_AUP_NAOSW_5_1_C0UW4A	0	0	0
	Darmor_AUP_PAOSW_6_1_C0UW4AC	3	0.03984651	0.03984651
	Ningyou_ERR037335	0	2.44E-45	0
	TN04_ERR037297	0	0	0
	TN05_ERR037298	0	0	0
	TN08_ERR037301	0	0	0
	TN103_ERR037317	0	0	0
	TN108_ERR037318	0	0	0
	TN109_ERR037319	0	8.77E-33	0
	TN10_ERR037294	0	0	0
	TN121_ERR037320	0	0	0
	TN124_ERR037321	0	0	0
	TN126_ERR037322	0	0	0
	TN128_ERR037323	0	0	0
	TN129_ERR037324	0	2.05E-44	0
	TN12_ERR037302	0	0	0
	TN138_ERR037325	0	0	0
	TN149_ERR037326	0	0	0
	TN161_ERR037327	0	0	0
	TN163_ERR037328	0	0	0

	TN170_ERR037329	0	0	0
	TN171_ERR037332	0	0	0
	TN172_ERR037333	0	0	0
	TN176_ERR037330	0	6.66E-27	0
	TN177_ERR037331	0	0	0
	TN24_ERR037299	0	0	0
	TN28_ERR037295	0	0	0
	TN30_ERR037305	0	0	0
	TN31_ERR037303	0	0	0
	TN38_ERR037304	0	0	0
	TN44_ERR037306	0	0	0
	TN48_ERR037307	0	0	0
	TN51_ERR037308	0	0	0
	TN57_ERR037309	0	0	0
	TN72_ERR037312	0	0	0
	TN76_ERR037311	0	0	0
	TN77_ERR037310	0	0	0
	TN85_ERR037315	0	0	0
	TN88_ERR037313	0	0	0
	TN90_ERR037316	0	0	0
	Tapidor_ERR037334	0	0	0
BnaA05g15540.1D2	Darmor_AUP_AOSW_2_D09BTACXX.I	0	0	0
	Darmor_AUP_BOSW_3_D09BTACXX.I	0	0	0
	Darmor_AUP_DOSW_2_D09BTACXX.I	0	0	0
	Darmor_AUP_FOSW_4_D09BTACXX.II	0	0	0
	Darmor_AUP_HOSW_5_D09BTACXX.I	0	0	0
	Darmor_AUP_IAOSW_1_1_C0UW4AC	0	0	0
	Darmor_AUP_KAOSW_2_1_C0UW4A	0	0	0
	Darmor_AUP_LAOSW_3_1_C0UW4A(	0	0	0
	Darmor_AUP_MAOSW_4_1_C0UW4A	0	0	0
	Darmor_AUP_NAOSW_5_1_C0UW4A	0	0	0
	Darmor_AUP_PAOSW_6_1_C0UW4A(	0	0	0
	Ningyou_ERR037335	0	0	0

	TN04_ERR037297	0	0	0
	TN05_ERR037298	0	0	0
	TN08_ERR037301	0	0	0
	TN103_ERR037317	0	0	0
	TN108_ERR037318	0	0	0
	TN109_ERR037319	0	0	0
	TN10_ERR037294	0	0	0
	TN121_ERR037320	0	0	0
	TN124_ERR037321	0	0	0
	TN126_ERR037322	0	0	0
	TN128_ERR037323	0	0	0
	TN129_ERR037324	0	0	0
	TN12_ERR037302	0	0	0
	TN138_ERR037325	0	0	0
	TN149_ERR037326	0	0	0
	TN161_ERR037327	0	0	0
	TN163_ERR037328	0	0	0
	TN170_ERR037329	0	0	0
	TN171_ERR037332	0	0	0
	TN172_ERR037333	0	0	0
	TN176_ERR037330	0	0	0
	TN177_ERR037331	0	0	0
	TN24_ERR037299	0	0	0
	TN28_ERR037295	0	0	0
	TN30_ERR037305	0	0	0
	TN31_ERR037303	0	0	0
	TN38_ERR037304	0	0	0
	TN44_ERR037306	0	0	0
	TN48_ERR037307	0	0	0
	TN51_ERR037308	0	0	0
	TN57_ERR037309	0	0	0
	TN72_ERR037312	0	0	0
	TN76_ERR037311	0	0	0

	TN77_ERR037310	0	0	0
	TN85_ERR037315	0	0	0
	TN88_ERR037313	0	0	0
	TN90_ERR037316	0	0	0
	Tapidor_ERR037334	0	0	0
BnaC02g07770.1D2	Darmor_AUP_AOSW_2_D09BTACXX.I	9	3.278829	0
	Darmor_AUP_BOSW_3_D09BTACXX.I	5	2.920688	2.096864
	Darmor_AUP_DOSW_2_D09BTACXX.I	9	7.346969	3.111699
	Darmor_AUP_FOSW_4_D09BTACXX.II	75	51.45008	39.90045
	Darmor_AUP_HOSW_5_D09BTACXX.I	8	6.731986	1.85505
	Darmor_AUP_IAOSW_1_1_C0UW4A(	17	0.7292452	0.6642075
	Darmor_AUP_KAOSW_2_1_C0UW4A	25	1.717391	0.9438207
	Darmor_AUP_LAOSW_3_1_C0UW4A(	486	41.04238	36.27229
	Darmor_AUP_MAOSW_4_1_C0UW4/	546	48.71424	43.40487
	Darmor_AUP_NAOSW_5_1_C0UW4A	304	30.95781	26.53315
	Darmor_AUP_PAOSW_6_1_C0UW4A(	4	0.449109	0
	Ningyou_ERR037335	1	0.182466	0.1823432
	TN04_ERR037297	0	4.14E-25	0
	TN05_ERR037298	0	1.21E-17	0
	TN08_ERR037301	0	4.21E-26	0
	TN103_ERR037317	0	4.07E-22	0
	TN108_ERR037318	0	2.46E-25	0
	TN109_ERR037319	0	1.69E-26	0
	TN10_ERR037294	1	0.1529709	0
	TN121_ERR037320	0	1.86E-26	0
	TN124_ERR037321	0	3.94E-24	0
	TN126_ERR037322	0	1.97E-23	0
	TN128_ERR037323	0	2.22E-19	0
	TN129_ERR037324	0	4.85E-22	0
	TN12_ERR037302	0	7.65E-19	0
	TN138_ERR037325	0	1.74E-25	0
	TN149_ERR037326	0	2.69E-25	0
	TN161_ERR037327	0	6.35E-26	0

	TN163_ERR037328	0	1.77E-18	0
	TN170_ERR037329	0	4.99E-24	0
	TN171_ERR037332	0	1.09E-20	0
	TN172_ERR037333	1	0.138145	0.1342519
	TN176_ERR037330	1	1.49636	1.473566
	TN177_ERR037331	0	1.65E-11	0
	TN24_ERR037299	0	1.38E-22	0
	TN28_ERR037295	0	3.16E-11	0
	TN30_ERR037305	0	1.40E-25	0
	TN31_ERR037303	2	0.3266913	0.2750329
	TN38_ERR037304	0	3.12E-20	0
	TN44_ERR037306	0	3.46E-18	0
	TN48_ERR037307	0	9.75E-24	0
	TN51_ERR037308	0	9.61E-18	0
	TN57_ERR037309	0	2.59E-22	0
	TN72_ERR037312	0	2.72E-23	0
	TN76_ERR037311	0	7.20E-20	0
	TN77_ERR037310	0	4.45E-25	0
	TN85_ERR037315	0	3.53E-25	0
	TN88_ERR037313	0	9.83E-23	0
	TN90_ERR037316	0	1.07E-22	0
	Tapidor_ERR037334	1	0.1582819	0.1209907
BnaA09g14660.1D2	Darmor_AUP_AOSW_2_D09BTACXX.I	0	0	0
	Darmor_AUP_BOSW_3_D09BTACXX.I	0	0	0
	Darmor_AUP_DOSW_2_D09BTACXX.I	0	0	0
	Darmor_AUP_FOSW_4_D09BTACXX.II	0	0	0
	Darmor_AUP_HOSW_5_D09BTACXX.I	0	0	0
	Darmor_AUP_IAOSW_1_1_C0UW4AC	0	0	0
	Darmor_AUP_KAOSW_2_1_C0UW4A	0	0	0
	Darmor_AUP_LAOSW_3_1_C0UW4AC	0	0	0
	Darmor_AUP_MAOSW_4_1_C0UW4A	0	0	0
	Darmor_AUP_NAOSW_5_1_C0UW4A	0	0	0
	Darmor_AUP_PAOSW_6_1_C0UW4AC	0	0	0

	Ningyou_ERR037335	0	0	0
	TN04_ERR037297	0	0	0
	TN05_ERR037298	0	0	0
	TN08_ERR037301	0	0	0
	TN103_ERR037317	0	0	0
	TN108_ERR037318	0	0	0
	TN109_ERR037319	0	0	0
	TN10_ERR037294	0	0	0
	TN121_ERR037320	0	0	0
	TN124_ERR037321	0	0	0
	TN126_ERR037322	0	0	0
	TN128_ERR037323	0	0	0
	TN129_ERR037324	0	0	0
	TN12_ERR037302	0	0	0
	TN138_ERR037325	0	0	0
	TN149_ERR037326	0	0	0
	TN161_ERR037327	0	0	0
	TN163_ERR037328	0	0	0
	TN170_ERR037329	0	0	0
	TN171_ERR037332	0	0	0
	TN172_ERR037333	0	0	0
	TN176_ERR037330	0	0	0
	TN177_ERR037331	0	0	0
	TN24_ERR037299	0	0	0
	TN28_ERR037295	0	0	0
	TN30_ERR037305	0	0	0
	TN31_ERR037303	0	0	0
	TN38_ERR037304	0	0	0
	TN44_ERR037306	0	0	0
	TN48_ERR037307	0	0	0
	TN51_ERR037308	0	0	0
	TN57_ERR037309	0	0	0
	TN72_ERR037312	0	0	0

	TN76_ERR037311	0	0	0
	TN77_ERR037310	0	0	0
	TN85_ERR037315	0	0	0
	TN88_ERR037313	0	0	0
	TN90_ERR037316	0	0	0
	Tapidor_ERR037334	0	0	0
BnaA01g31810.1D2	Darmor_AUP_AOSW_2_D09BTACXX.I	0	3.08E-39	0
	Darmor_AUP_BOSW_3_D09BTACXX.I	0	2.37E-32	0
	Darmor_AUP_DOSW_2_D09BTACXX.I	0	3.78E-38	0
	Darmor_AUP_FOSW_4_D09BTACXX.I	0	0.006880779	0
	Darmor_AUP_HOSW_5_D09BTACXX.I	0	0.2687108	0
	Darmor_AUP_IAOSW_1_1_C0UW4AC	0	0	0
	Darmor_AUP_KAOSW_2_1_C0UW4A	0	0	0
	Darmor_AUP_LAOSW_3_1_C0UW4AC	0	0	0
	Darmor_AUP_MAOSW_4_1_C0UW4A	0	0.002769109	0
	Darmor_AUP_NAOSW_5_1_C0UW4A	0	0	0
	Darmor_AUP_PAOSW_6_1_C0UW4AC	0	1.18E-48	0
	Ningyou_ERR037335	0	1.88E-06	0
	TN04_ERR037297	0	3.18E-43	0
	TN05_ERR037298	0	3.00E-35	0
	TN08_ERR037301	0	0	0
	TN103_ERR037317	0	2.64E-32	0
	TN108_ERR037318	0	3.07E-28	0
	TN109_ERR037319	0	0	0
	TN10_ERR037294	0	1.32E-37	0
	TN121_ERR037320	0	5.98E-33	0
	TN124_ERR037321	0	3.05E-09	0
	TN126_ERR037322	0	8.86E-34	0
	TN128_ERR037323	0	0	0
	TN129_ERR037324	0	2.47E-25	0
	TN12_ERR037302	0	1.90E-31	0
	TN138_ERR037325	0	6.41E-32	0
	TN149_ERR037326	0	4.88E-27	0

	TN161_ERR037327	0	2.02E-31	0
	TN163_ERR037328	0	2.35E-27	0
	TN170_ERR037329	0	1.25E-09	0
	TN171_ERR037332	0	4.61E-35	0
	TN172_ERR037333	0	6.79E-40	0
	TN176_ERR037330	0	1.63E-45	0
	TN177_ERR037331	0	6.50E-06	0
	TN24_ERR037299	0	1.84E-25	0
	TN28_ERR037295	0	2.82E-33	0
	TN30_ERR037305	0	3.71E-31	0
	TN31_ERR037303	0	4.06E-31	0
	TN38_ERR037304	0	2.80E-35	0
	TN44_ERR037306	0	0.001789722	0
	TN48_ERR037307	0	1.80E-29	0
	TN51_ERR037308	0	1.28E-27	0
	TN57_ERR037309	0	1.29E-32	0
	TN72_ERR037312	0	5.76E-32	0
	TN76_ERR037311	0	7.32E-33	0
	TN77_ERR037310	0	1.47E-41	0
	TN85_ERR037315	0	0	0
	TN88_ERR037313	0	8.18E-33	0
	TN90_ERR037316	0	0	0
	Tapidor_ERR037334	0	0.00150621	0
BnaC02g10520.1D2	Darmor_AUP_AOSW_2_D09BTACXX.I	255	6.198481	6.175581
	Darmor_AUP_BOSW_3_D09BTACXX.I	579	17.3758	17.06444
	Darmor_AUP_DOSW_2_D09BTACXX.I	273	6.061403	5.57572
	Darmor_AUP_FOSW_4_D09BTACXX.II	187	5.191876	5.190859
	Darmor_AUP_HOSW_5_D09BTACXX.I	80	2.524683	2.523177
	Darmor_AUP_IAOSW_1_1_C0UW4AC	467	4.732678	4.680868
	Darmor_AUP_KAOSW_2_1_C0UW4A	567	5.7592	5.759191
	Darmor_AUP_LAOSW_3_1_C0UW4A(	245	2.365455	2.365455
	Darmor_AUP_MAOSW_4_1_C0UW4A	274	2.632846	2.632846
	Darmor_AUP_NAOSW_5_1_C0UW4A	283	2.740802	2.740802

	Darmor_AUP_PAOSW_6_1_C0UW4A(	465	4.539664	4.539663
	Ningyou_ERR037335	0	3.44E-31	0
	TN04_ERR037297	0	3.22E-32	0
	TN05_ERR037298	0	5.59E-28	0
	TN08_ERR037301	0	0	0
	TN103_ERR037317	0	1.26E-25	0
	TN108_ERR037318	0	2.46E-28	0
	TN109_ERR037319	0	2.39E-32	0
	TN10_ERR037294	1	0.1400114	0.1400114
	TN121_ERR037320	0	4.39E-31	0
	TN124_ERR037321	0	1.73E-32	0
	TN126_ERR037322	0	1.57E-27	0
	TN128_ERR037323	0	4.87E-33	0
	TN129_ERR037324	0	2.97E-28	0
	TN12_ERR037302	0	1.52E-32	0
	TN138_ERR037325	0	1.65E-31	0
	TN149_ERR037326	0	1.71E-25	0
	TN161_ERR037327	0	2.19E-32	0
	TN163_ERR037328	0	6.75E-26	0
	TN170_ERR037329	0	4.83E-33	0
	TN171_ERR037332	0	1.13E-27	0
	TN172_ERR037333	0	7.98E-27	0
	TN176_ERR037330	0	3.40E-30	0
	TN177_ERR037331	0	1.61E-32	0
	TN24_ERR037299	0	8.61E-23	0
	TN28_ERR037295	0	6.34E-32	0
	TN30_ERR037305	0	1.48E-27	0
	TN31_ERR037303	0	9.09E-33	0
	TN38_ERR037304	0	2.47E-27	0
	TN44_ERR037306	0	2.26E-30	0
	TN48_ERR037307	0	0	0
	TN51_ERR037308	0	9.08E-31	0
	TN57_ERR037309	0	2.36E-33	0

	TN72_ERR037312	0	1.45E-26	0
	TN76_ERR037311	0	4.01E-27	0
	TN77_ERR037310	0	0	0
	TN85_ERR037315	0	2.86E-32	0
	TN88_ERR037313	0	3.49E-30	0
	TN90_ERR037316	0	1.43E-27	0
	Tapidor_ERR037334	0	2.45E-28	0
BnaC02g03370.1D2	Darmor_AUP_AOSW_2_D09BTACXX.I	37	3.385918	1.780796
	Darmor_AUP_BOSW_3_D09BTACXX.I	33	3.290473	1.528751
	Darmor_AUP_DOSW_2_D09BTACXX.I	54	4.215122	2.400275
	Darmor_AUP_FOSW_4_D09BTACXX.I	53	5.756592	4.45033
	Darmor_AUP_HOSW_5_D09BTACXX.I	47	6.57417	4.270597
	Darmor_AUP_IAOSW_1_1_C0UW4AC	120	2.240835	1.926212
	Darmor_AUP_KAOSW_2_1_C0UW4A	107	2.566721	2.026766
	Darmor_AUP_LAOSW_3_1_C0UW4AC	100	3.043608	2.317623
	Darmor_AUP_MAOSW_4_1_C0UW4A	124	3.488815	3.097148
	Darmor_AUP_NAOSW_5_1_C0UW4A	89	2.230323	1.507074
	Darmor_AUP_PAOSW_6_1_C0UW4AC	135	3.289329	2.186153
	Ningyou_ERR037335	15	3.677173	1.419031
	TN04_ERR037297	9	0.5288692	0.03294517
	TN05_ERR037298	9	1.33778	0.1528431
	TN08_ERR037301	10	1.587948	1.024508
	TN103_ERR037317	12	2.084146	0.07112644
	TN108_ERR037318	9	2.772641	1.680867
	TN109_ERR037319	9	1.703404	0.926589
	TN10_ERR037294	8	0.3920883	0
	TN121_ERR037320	10	0.9307568	0.1010623
	TN124_ERR037321	11	0.9294572	0.3312731
	TN126_ERR037322	9	0.8892498	0.1965928
	TN128_ERR037323	0	1.09E-15	0
	TN129_ERR037324	13	1.940466	0.1406476
	TN12_ERR037302	11	0.7316539	0.6555316
	TN138_ERR037325	10	1.098413	0.1451567

	TN149_ERR037326	1	1.115998	0
	TN161_ERR037327	4	1.0828	0
	TN163_ERR037328	6	0.652399	0
	TN170_ERR037329	18	2.99238	1.243176
	TN171_ERR037332	0	8.24E-18	0
	TN172_ERR037333	26	3.351587	1.693299
	TN176_ERR037330	13	1.573632	0.6188133
	TN177_ERR037331	3	0.5700647	0.3396167
	TN24_ERR037299	0	4.25E-24	0
	TN28_ERR037295	19	2.617638	0.8515422
	TN30_ERR037305	8	0.7677376	0.09433958
	TN31_ERR037303	14	2.460712	0.7616881
	TN38_ERR037304	15	1.632939	1.118899
	TN44_ERR037306	6	3.033562	0
	TN48_ERR037307	11	1.579774	1.048517
	TN51_ERR037308	15	0.9962269	0.6361275
	TN57_ERR037309	6	0.6039972	0.3778473
	TN72_ERR037312	5	0.4239768	0
	TN76_ERR037311	7	0.6151907	0.1149255
	TN77_ERR037310	0	3.90E-19	0
	TN85_ERR037315	7	0.4342507	0
	TN88_ERR037313	0	7.05E-14	0
	TN90_ERR037316	4	0.4147017	0
	Tapidor_ERR037334	2	0.1286351	0.01080194
BnaU01g31710.1D2	Darmor_AUP_AOSW_2_D09BTACXX.I	0	7.98E-27	0
	Darmor_AUP_BOSW_3_D09BTACXX.I	0	3.08E-20	0
	Darmor_AUP_DOSW_2_D09BTACXX.I	0	3.05E-32	0
	Darmor_AUP_FOSW_4_D09BTACXX.II	0	1.29E-24	0
	Darmor_AUP_HOSW_5_D09BTACXX.I	0	3.57E-26	0
	Darmor_AUP_IAOSW_1_1_C0UW4A(	0	0	0
	Darmor_AUP_KAOSW_2_1_C0UW4A	0	0	0
	Darmor_AUP_LAOSW_3_1_C0UW4A(	0	1.61E-41	0
	Darmor_AUP_MAOSW_4_1_C0UW4/	0	1.84E-32	0

	Darmor_AUP_NAOSW_5_1_C0UW4A	0	1.39E-41	0
	Darmor_AUP_PAOSW_6_1_C0UW4A(	0	1.01E-31	0
	Ningyou_ERR037335	0	0	0
	TN04_ERR037297	0	1.02E-20	0
	TN05_ERR037298	0	0	0
	TN08_ERR037301	0	0	0
	TN103_ERR037317	0	0	0
	TN108_ERR037318	0	0	0
	TN109_ERR037319	0	0.00810339	0
	TN10_ERR037294	0	7.68E-07	0
	TN121_ERR037320	0	0.4372623	0.03960255
	TN124_ERR037321	0	0	0
	TN126_ERR037322	0	1.84E-19	0
	TN128_ERR037323	0	1.86E-26	0
	TN129_ERR037324	0	0	0
	TN12_ERR037302	0	8.13E-06	0
	TN138_ERR037325	0	0	0
	TN149_ERR037326	0	0	0
	TN161_ERR037327	0	0	0
	TN163_ERR037328	0	0	0
	TN170_ERR037329	0	0	0
	TN171_ERR037332	0	2.48E-19	0
	TN172_ERR037333	0	0	0
	TN176_ERR037330	0	0	0
	TN177_ERR037331	0	8.80E-26	0
	TN24_ERR037299	0	3.90E-25	0
	TN28_ERR037295	0	3.83E-06	0
	TN30_ERR037305	0	0	0
	TN31_ERR037303	0	2.67E-56	0
	TN38_ERR037304	0	0.1250446	0
	TN44_ERR037306	0	8.64E-21	0
	TN48_ERR037307	0	2.23E-22	0
	TN51_ERR037308	0	0.001666115	0

	TN57_ERR037309	0	2.94E-24	0
	TN72_ERR037312	0	2.11E-24	0
	TN76_ERR037311	0	8.92E-28	0
	TN77_ERR037310	0	6.09E-26	0
	TN85_ERR037315	0	1.67E-12	0
	TN88_ERR037313	0	0	0
	TN90_ERR037316	0	8.30E-22	0
	Tapidor_ERR037334	0	3.53E-28	0
BnaC05g10050.1D2	Darmor_AUP_AOSW_2_D09BTACXX.I	0	1.31E-13	0
	Darmor_AUP_BOSW_3_D09BTACXX.I	0	1.08E-20	0
	Darmor_AUP_DOSW_2_D09BTACXX.I	0	2.60E-08	0
	Darmor_AUP_FOSW_4_D09BTACXX.II	0	2.06E-10	0
	Darmor_AUP_HOSW_5_D09BTACXX.I	0	2.84E-16	0
	Darmor_AUP_IAOSW_1_1_C0UW4AC	0	1.75E-26	0
	Darmor_AUP_KAOSW_2_1_C0UW4A	0	4.88E-25	0
	Darmor_AUP_LAOSW_3_1_C0UW4AC	0	8.48E-12	0
	Darmor_AUP_MAOSW_4_1_C0UW4A	0	1.886234	0
	Darmor_AUP_NAOSW_5_1_C0UW4A	0	8.10E-17	0
	Darmor_AUP_PAOSW_6_1_C0UW4AC	0	0.005270218	0
	Ningyou_ERR037335	0	1.09E-23	0
	TN04_ERR037297	0	2.45E-47	0
	TN05_ERR037298	0	2.99E-38	0
	TN08_ERR037301	0	1.66E-46	0
	TN103_ERR037317	0	1.89E-52	0
	TN108_ERR037318	0	2.57E-31	0
	TN109_ERR037319	0	1.20E-44	0
	TN10_ERR037294	0	7.59E-25	0
	TN121_ERR037320	0	1.61E-44	0
	TN124_ERR037321	0	1.77E-54	0
	TN126_ERR037322	0	3.73E-24	0
	TN128_ERR037323	0	3.93E-33	0
	TN129_ERR037324	0	1.57E-40	0
	TN12_ERR037302	0	5.66E-45	0

	TN138_ERR037325	0	1.22E-61	0
	TN149_ERR037326	0	0	0
	TN161_ERR037327	0	1.43E-33	0
	TN163_ERR037328	0	1.96E-38	0
	TN170_ERR037329	0	1.92E-38	0
	TN171_ERR037332	0	2.26E-66	0
	TN172_ERR037333	0	9.09E-30	0
	TN176_ERR037330	0	1.50E-30	0
	TN177_ERR037331	0	1.03E-28	0
	TN24_ERR037299	0	2.71E-38	0
	TN28_ERR037295	0	8.19E-17	0
	TN30_ERR037305	0	8.03E-45	0
	TN31_ERR037303	0	9.10E-37	0
	TN38_ERR037304	0	2.71E-44	0
	TN44_ERR037306	0	2.60E-22	0
	TN48_ERR037307	0	7.30E-50	0
	TN51_ERR037308	0	9.14E-37	0
	TN57_ERR037309	0	5.98E-65	0
	TN72_ERR037312	0	8.60E-31	0
	TN76_ERR037311	0	1.67E-78	0
	TN77_ERR037310	0	1.81E-60	0
	TN85_ERR037315	0	2.08E-47	0
	TN88_ERR037313	0	9.47E-37	0
	TN90_ERR037316	0	1.38E-76	0
	Tapidor_ERR037334	0	1.02E-22	0
BnaC02g03220.1D2	Darmor_AUP_AOSW_2_D09BTACXX.I	169	20.96955	16.96161
	Darmor_AUP_BOSW_3_D09BTACXX.I	197	30.00217	25.07581
	Darmor_AUP_DOSW_2_D09BTACXX.I	190	18.56191	15.31231
	Darmor_AUP_FOSW_4_D09BTACXX.II	116	15.77239	12.25229
	Darmor_AUP_HOSW_5_D09BTACXX.I	111	18.64124	13.96175
	Darmor_AUP_IAOSW_1_1_C0UW4A(	741	23.21722	21.22982
	Darmor_AUP_KAOSW_2_1_C0UW4A	757	27.32497	24.66639
	Darmor_AUP_LAOSW_3_1_C0UW4A(	373	13.69043	12.0909

	Darmor_AUP_MAOSW_4_1_C0UW4A	366	12.38897	10.86817
	Darmor_AUP_NAOSW_5_1_C0UW4A	335	11.24558	9.55752
	Darmor_AUP_PAOSW_6_1_C0UW4A(	766	25.99584	23.59256
	Ningyou_ERR037335	37	23.23785	16.72781
	TN04_ERR037297	16	7.70207	4.657971
	TN05_ERR037298	45	17.62066	12.36589
	TN08_ERR037301	19	12.73978	7.193807
	TN103_ERR037317	44	18.03993	11.59847
	TN108_ERR037318	44	19.27833	13.04654
	TN109_ERR037319	54	24.94026	18.89348
	TN10_ERR037294	23	13.42443	8.047812
	TN121_ERR037320	40	20.45645	14.13355
	TN124_ERR037321	24	9.734604	5.362092
	TN126_ERR037322	32	13.59618	8.981635
	TN128_ERR037323	0	4.40E-18	0
	TN129_ERR037324	67	30.63373	24.06948
	TN12_ERR037302	19	10.60784	6.096238
	TN138_ERR037325	50	22.78741	16.28422
	TN149_ERR037326	27	8.241108	3.773446
	TN161_ERR037327	8	6.089457	1.86022
	TN163_ERR037328	18	12.53293	7.132315
	TN170_ERR037329	65	22.36911	17.45742
	TN171_ERR037332	0	1.37E-22	0
	TN172_ERR037333	53	20.09167	14.75089
	TN176_ERR037330	47	17.30037	12.79341
	TN177_ERR037331	14	5.816994	2.932144
	TN24_ERR037299	0	7.01E-16	0
	TN28_ERR037295	44	21.4049	15.53173
	TN30_ERR037305	28	16.45665	10.93775
	TN31_ERR037303	40	15.45619	10.86985
	TN38_ERR037304	37	19.79663	13.60916
	TN44_ERR037306	17	4.227658	2.386678
	TN48_ERR037307	20	13.68047	8.69017

	TN51_ERR037308	31	18.35626	10.67137
	TN57_ERR037309	12	5.744418	2.194283
	TN72_ERR037312	19	9.542981	5.115403
	TN76_ERR037311	19	6.543863	3.66483
	TN77_ERR037310	0	2.06E-19	0
	TN85_ERR037315	13	11.73611	6.852141
	TN88_ERR037313	0	1.24E-21	0
	TN90_ERR037316	20	8.823033	4.969911
	Tapidor_ERR037334	0	2.00E-15	0
BnaC02g06920.1D2	Darmor_AUP_AOSW_2_D09BTACXX.I	2	0.1416902	0
	Darmor_AUP_BOSW_3_D09BTACXX.I	1	0.09414451	0.09414451
	Darmor_AUP_DOSW_2_D09BTACXX.I	0	0	0
	Darmor_AUP_FOSW_4_D09BTACXX.II	0	3.11E-09	0
	Darmor_AUP_HOSW_5_D09BTACXX.I	12	1.215308	0.7590646
	Darmor_AUP_IAOSW_1_1_C0UW4AC	0	0	0
	Darmor_AUP_KAOSW_2_1_C0UW4A	1	0.03302563	0.03302563
	Darmor_AUP_LAOSW_3_1_C0UW4AC	0	0	0
	Darmor_AUP_MAOSW_4_1_C0UW4A	0	0	0
	Darmor_AUP_NAOSW_5_1_C0UW4A	0	0	0
	Darmor_AUP_PAOSW_6_1_C0UW4AC	1	0.03198871	0.03198871
	Ningyou_ERR037335	0	2.44E-44	0
	TN04_ERR037297	0	0	0
	TN05_ERR037298	0	0	0
	TN08_ERR037301	0	0	0
	TN103_ERR037317	0	0	0
	TN108_ERR037318	0	0	0
	TN109_ERR037319	0	0	0
	TN10_ERR037294	0	2.38E-31	0
	TN121_ERR037320	0	0	0
	TN124_ERR037321	0	0	0
	TN126_ERR037322	0	0	0
	TN128_ERR037323	0	7.38E-33	0
	TN129_ERR037324	0	1.12E-23	0

	TN12_ERR037302	0	1.07E-42	0
	TN138_ERR037325	0	7.25E-44	0
	TN149_ERR037326	0	0	0
	TN161_ERR037327	0	0	0
	TN163_ERR037328	0	0	0
	TN170_ERR037329	0	0	0
	TN171_ERR037332	0	0	0
	TN172_ERR037333	0	2.74E-43	0
	TN176_ERR037330	0	3.51E-42	0
	TN177_ERR037331	0	0	0
	TN24_ERR037299	0	7.03E-41	0
	TN28_ERR037295	0	1.06E-54	0
	TN30_ERR037305	0	4.01E-45	0
	TN31_ERR037303	0	0	0
	TN38_ERR037304	0	1.36E-35	0
	TN44_ERR037306	0	0	0
	TN48_ERR037307	0	0	0
	TN51_ERR037308	0	5.03E-40	0
	TN57_ERR037309	0	0	0
	TN72_ERR037312	0	0	0
	TN76_ERR037311	0	1.61E-33	0
	TN77_ERR037310	0	0	0
	TN85_ERR037315	0	3.09E-51	0
	TN88_ERR037313	0	2.25E-43	0
	TN90_ERR037316	0	0	0
	Tapidor_ERR037334	0	0	0
BnaC02g04350.1D2	Darmor_AUP_AOSW_2_D09BTACXX.I	24	0.8937709	0.3863646
	Darmor_AUP_BOSW_3_D09BTACXX.I	41	1.659599	1.587123
	Darmor_AUP_DOSW_2_D09BTACXX.I	84	3.108633	2.249533
	Darmor_AUP_FOSW_4_D09BTACXX.II	64	2.74	2.612317
	Darmor_AUP_HOSW_5_D09BTACXX.I	46	1.823216	1.796875
	Darmor_AUP_IAOSW_1_1_C0UW4AC	51	0.6569947	0.6569947
	Darmor_AUP_KAOSW_2_1_C0UW4A	45	0.5850607	0.5846903

	Darmor_AUP_LAOSW_3_1_C0UW4A(	95	1.299188	1.244231
	Darmor_AUP_MAOSW_4_1_C0UW4/	90	1.289147	1.289081
	Darmor_AUP_NAOSW_5_1_C0UW4A	75	1.062947	0.9810717
	Darmor_AUP_PAOSW_6_1_C0UW4A(	66	0.8919591	0.8919591
	Ningyou_ERR037335	28	2.331818	1.950158
	TN04_ERR037297	15	0.6799302	0.6772901
	TN05_ERR037298	38	2.227954	2.086886
	TN08_ERR037301	7	0.3410532	0.3206014
	TN103_ERR037317	13	0.8443124	0.3697962
	TN108_ERR037318	19	1.302149	0.8698711
	TN109_ERR037319	44	2.566087	1.517514
	TN10_ERR037294	16	0.9091798	0.8402331
	TN121_ERR037320	26	1.649769	0.6736957
	TN124_ERR037321	10	0.3892028	0.3889339
	TN126_ERR037322	33	1.747416	1.465549
	TN128_ERR037323	0	7.01E-30	0
	TN129_ERR037324	30	1.50935	1.352831
	TN12_ERR037302	19	1.448127	0.6637716
	TN138_ERR037325	22	1.723771	0.08067859
	TN149_ERR037326	14	1.010547	0.966189
	TN161_ERR037327	4	0.213289	0.2052732
	TN163_ERR037328	15	0.8149331	0.6855029
	TN170_ERR037329	33	1.745697	1.524618
	TN171_ERR037332	0	2.92E-17	0
	TN172_ERR037333	16	0.9491174	0.6377404
	TN176_ERR037330	24	1.321613	1.198966
	TN177_ERR037331	10	0.4208537	0.3998442
	TN24_ERR037299	0	1.50E-27	0
	TN28_ERR037295	27	1.345845	1.342792
	TN30_ERR037305	24	1.651419	1.238783
	TN31_ERR037303	10	0.9458498	0
	TN38_ERR037304	34	1.8531	1.795574
	TN44_ERR037306	10	0.6486333	0.6486332

	TN48_ERR037307	14	1.072789	0.1618884
	TN51_ERR037308	19	1.339832	1.125904
	TN57_ERR037309	8	0.4500798	0.449978
	TN72_ERR037312	14	1.033328	0.1973336
	TN76_ERR037311	12	0.6968725	0.6945083
	TN77_ERR037310	0	3.38E-28	0
	TN85_ERR037315	17	1.617145	0.8310468
	TN88_ERR037313	0	1.31E-26	0
	TN90_ERR037316	12	0.8151158	0
	Tapidor_ERR037334	0	1.33E-27	0
BnaC03g02100.1D2	Darmor_AUP_AOSW_2_D09BTACXX.I	92	2.292845	2.179446
	Darmor_AUP_BOSW_3_D09BTACXX.I	141	4.777481	4.672142
	Darmor_AUP_DOSW_2_D09BTACXX.I	201	5.317728	4.491087
	Darmor_AUP_FOSW_4_D09BTACXX.II	41	1.18529	0.5649439
	Darmor_AUP_HOSW_5_D09BTACXX.I	175	6.237941	5.641443
	Darmor_AUP_IAOSW_1_1_C0UW4AC	240	2.968271	2.968271
	Darmor_AUP_KAOSW_2_1_C0UW4A	165	2.039423	2.038511
	Darmor_AUP_LAOSW_3_1_C0UW4AC	37	0.4391514	0.4391326
	Darmor_AUP_MAOSW_4_1_C0UW4A	59	0.6986326	0.6986326
	Darmor_AUP_NAOSW_5_1_C0UW4A	55	0.6756692	0.6756692
	Darmor_AUP_PAOSW_6_1_C0UW4AC	216	2.66888	2.651218
	Ningyou_ERR037335	7	0.753297	0.5341889
	TN04_ERR037297	0	4.91E-24	0
	TN05_ERR037298	0	8.35E-29	0
	TN08_ERR037301	0	7.02E-32	0
	TN103_ERR037317	0	5.97E-17	0
	TN108_ERR037318	0	7.00E-27	0
	TN109_ERR037319	0	7.11E-31	0
	TN10_ERR037294	0	5.27E-30	0
	TN121_ERR037320	0	3.94E-31	0
	TN124_ERR037321	0	1.82E-26	0
	TN126_ERR037322	0	4.72E-24	0
	TN128_ERR037323	0	2.77E-27	0

	TN129_ERR037324	0	1.09E-12	0
	TN12_ERR037302	0	6.54E-27	0
	TN138_ERR037325	0	2.71E-16	0
	TN149_ERR037326	0	7.20E-32	0
	TN161_ERR037327	16	0.7581255	0.06123296
	TN163_ERR037328	4	0.1553289	0.107052
	TN170_ERR037329	0	1.48E-20	0
	TN171_ERR037332	0	1.69E-30	0
	TN172_ERR037333	0	2.97E-27	0
	TN176_ERR037330	0	1.23E-29	0
	TN177_ERR037331	0	5.72E-28	0
	TN24_ERR037299	0	9.44E-25	0
	TN28_ERR037295	0	2.92E-26	0
	TN30_ERR037305	0	4.54E-27	0
	TN31_ERR037303	12	0.4728734	0.4719394
	TN38_ERR037304	2	0.08448883	0.0840634
	TN44_ERR037306	0	1.43E-27	0
	TN48_ERR037307	0	3.75E-26	0
	TN51_ERR037308	10	0.5104325	0.5083398
	TN57_ERR037309	22	1.314837	0.9122036
	TN72_ERR037312	1	0.04255361	0.04237481
	TN76_ERR037311	0	1.41E-30	0
	TN77_ERR037310	22	1.084934	1.036147
	TN85_ERR037315	0	1.22E-30	0
	TN88_ERR037313	0	1.51E-05	1.51E-05
	TN90_ERR037316	0	5.29E-18	0
	Tapidor_ERR037334	0	6.02E-26	0
BnaC02g05440.1D2	Darmor_AUP_AOSW_2_D09BTACXX.I	71	1.338373	1.013472
	Darmor_AUP_BOSW_3_D09BTACXX.I	45	0.7859965	0.6078124
	Darmor_AUP_DOSW_2_D09BTACXX.I	272	4.712979	4.004203
	Darmor_AUP_FOSW_4_D09BTACXX.I	554	10.09104	9.161122
	Darmor_AUP_HOSW_5_D09BTACXX.I	287	7.625237	6.723359
	Darmor_AUP_IAOSW_1_1_C0UW4AC	150	0.9391342	0.7108083

	Darmor_AUP_KAOSW_2_1_C0UW4A	245	1.503172	1.240554
	Darmor_AUP_LAOSW_3_1_C0UW4A(	908	6.94229	6.47645
	Darmor_AUP_MAOSW_4_1_C0UW4A	1006	6.774535	6.337306
	Darmor_AUP_NAOSW_5_1_C0UW4A	836	5.791434	5.325412
	Darmor_AUP_PAOSW_6_1_C0UW4A(	185	1.247655	1.090511
	Ningyou_ERR037335	92	4.436809	3.112474
	TN04_ERR037297	41	1.372154	1.07304
	TN05_ERR037298	35	1.32158	0.5683977
	TN08_ERR037301	39	2.171228	1.244266
	TN103_ERR037317	30	1.566122	0.9013454
	TN108_ERR037318	61	2.988277	1.854379
	TN109_ERR037319	37	1.508052	1.117087
	TN10_ERR037294	76	2.980434	2.115144
	TN121_ERR037320	47	1.795502	1.127892
	TN124_ERR037321	64	2.213752	1.453065
	TN126_ERR037322	57	2.109695	1.356265
	TN128_ERR037323	21	0.3597158	0.2050726
	TN129_ERR037324	56	1.810289	1.120609
	TN12_ERR037302	20	1.017919	0.4141487
	TN138_ERR037325	38	1.717894	0.730578
	TN149_ERR037326	51	2.350522	1.602281
	TN161_ERR037327	32	1.119221	0.3829264
	TN163_ERR037328	80	3.140814	2.31996
	TN170_ERR037329	120	4.36331	3.400778
	TN171_ERR037332	23	0.7808509	0.7177589
	TN172_ERR037333	160	5.817521	4.674199
	TN176_ERR037330	142	4.179513	3.391567
	TN177_ERR037331	44	2.264407	1.418622
	TN24_ERR037299	24	0.8262458	0.6093173
	TN28_ERR037295	61	1.660888	1.111439
	TN30_ERR037305	30	1.094804	0.8189088
	TN31_ERR037303	82	2.816818	2.18869
	TN38_ERR037304	27	1.432892	1.404864

	TN44_ERR037306	23	1.447145	0.6942377
	TN48_ERR037307	61	1.788088	1.17161
	TN51_ERR037308	59	3.510073	2.459963
	TN57_ERR037309	52	2.215532	1.490365
	TN72_ERR037312	49	1.710071	1.042962
	TN76_ERR037311	33	0.6770253	0.326686
	TN77_ERR037310	22	0.4308206	0.3748604
	TN85_ERR037315	32	1.522494	0.8121371
	TN88_ERR037313	29	0.5384924	0.3773688
	TN90_ERR037316	42	1.889933	1.328351
	Tapidor_ERR037334	27	0.5339163	0.2939051
BnaC07g30540.1D2	Darmor_AUP_AOSW_2_D09BTACXX.I	0	0	0
	Darmor_AUP_BOSW_3_D09BTACXX.I	0	0	0
	Darmor_AUP_DOSW_2_D09BTACXX.I	0	0	0
	Darmor_AUP_FOSW_4_D09BTACXX.I	0	0	0
	Darmor_AUP_HOSW_5_D09BTACXX.I	0	0	0
	Darmor_AUP_IAOSW_1_1_C0UW4A(	0	0	0
	Darmor_AUP_KAOSW_2_1_C0UW4A	0	0	0
	Darmor_AUP_LAOSW_3_1_C0UW4A(	0	0	0
	Darmor_AUP_MAOSW_4_1_C0UW4/	0	0	0
	Darmor_AUP_NAOSW_5_1_C0UW4A	0	0	0
	Darmor_AUP_PAOSW_6_1_C0UW4A(	0	3.79E-10	0
	Ningyou_ERR037335	0	0	0
	TN04_ERR037297	0	0	0
	TN05_ERR037298	0	0	0
	TN08_ERR037301	0	0	0
	TN103_ERR037317	1	0.1368345	0.1368345
	TN108_ERR037318	0	0	0
	TN109_ERR037319	0	0	0
	TN10_ERR037294	0	0	0
	TN121_ERR037320	0	0	0
	TN124_ERR037321	0	0	0
	TN126_ERR037322	0	0	0

	TN128_ERR037323	0	0	0
	TN129_ERR037324	0	0	0
	TN12_ERR037302	0	0	0
	TN138_ERR037325	0	0	0
	TN149_ERR037326	0	0	0
	TN161_ERR037327	0	0	0
	TN163_ERR037328	0	0	0
	TN170_ERR037329	0	0	0
	TN171_ERR037332	0	0	0
	TN172_ERR037333	2	0.0924063	0.0924063
	TN176_ERR037330	0	0	0
	TN177_ERR037331	0	0	0
	TN24_ERR037299	0	0	0
	TN28_ERR037295	0	0	0
	TN30_ERR037305	0	0	0
	TN31_ERR037303	0	0	0
	TN38_ERR037304	0	0	0
	TN44_ERR037306	0	0	0
	TN48_ERR037307	0	0	0
	TN51_ERR037308	0	0	0
	TN57_ERR037309	0	0	0
	TN72_ERR037312	0	0	0
	TN76_ERR037311	0	0	0
	TN77_ERR037310	0	0	0
	TN85_ERR037315	0	0	0
	TN88_ERR037313	0	0	0
	TN90_ERR037316	0	0	0
	Tapidor_ERR037334	0	0	0
BnaC02g07840.1D2	Darmor_AUP_AOSW_2_D09BTACXX.I	0	0.02014586	0.02012992
	Darmor_AUP_BOSW_3_D09BTACXX.I	1	0.0278033	0.0278033
	Darmor_AUP_DOSW_2_D09BTACXX.I	0	0	0
	Darmor_AUP_FOSW_4_D09BTACXX.II	0	0	0
	Darmor_AUP_HOSW_5_D09BTACXX.I	101	2.709835	2.500379

	Darmor_AUP_IAOSW_1_1_C0UW4A(	1	0.009116795	0.009116795
	Darmor_AUP_KAOSW_2_1_C0UW4A	2	0.01863739	0.01863739
	Darmor_AUP_LAOSW_3_1_C0UW4A(	0	0	0
	Darmor_AUP_MAOSW_4_1_C0UW4/	0	0	0
	Darmor_AUP_NAOSW_5_1_C0UW4A	0	0	0
	Darmor_AUP_PAOSW_6_1_C0UW4A(	1	0.00978798	0.00978798
	Ningyou_ERR037335	0	0	0
	TN04_ERR037297	0	0	0
	TN05_ERR037298	0	0	0
	TN08_ERR037301	0	0	0
	TN103_ERR037317	0	0	0
	TN108_ERR037318	0	0	0
	TN109_ERR037319	0	0	0
	TN10_ERR037294	0	0	0
	TN121_ERR037320	0	0	0
	TN124_ERR037321	0	0	0
	TN126_ERR037322	0	0	0
	TN128_ERR037323	0	0	0
	TN129_ERR037324	0	0	0
	TN12_ERR037302	0	0	0
	TN138_ERR037325	0	0.01435075	0
	TN149_ERR037326	0	0	0
	TN161_ERR037327	0	0	0
	TN163_ERR037328	0	0	0
	TN170_ERR037329	0	0	0
	TN171_ERR037332	0	0	0
	TN172_ERR037333	0	0	0
	TN176_ERR037330	0	0	0
	TN177_ERR037331	0	0	0
	TN24_ERR037299	0	0	0
	TN28_ERR037295	0	0	0
	TN30_ERR037305	0	0	0
	TN31_ERR037303	0	0	0

	TN38_ERR037304	0	0	0
	TN44_ERR037306	0	0	0
	TN48_ERR037307	0	0	0
	TN51_ERR037308	0	0	0
	TN57_ERR037309	0	0	0
	TN72_ERR037312	0	0	0
	TN76_ERR037311	0	3.61E-06	0
	TN77_ERR037310	0	0	0
	TN85_ERR037315	0	0	0
	TN88_ERR037313	0	0	0
	TN90_ERR037316	0	0	0
	Tapidor_ERR037334	0	0	0
BnaC02g10350.1D2	Darmor_AUP_AOSW_2_D09BTACXX.I	0	0	0
	Darmor_AUP_BOSW_3_D09BTACXX.I	0	0	0
	Darmor_AUP_DOSW_2_D09BTACXX.I	0	0	0
	Darmor_AUP_FOSW_4_D09BTACXX.II	0	0	0
	Darmor_AUP_HOSW_5_D09BTACXX.I	0	0	0
	Darmor_AUP_IAOSW_1_1_C0UW4AC	0	0	0
	Darmor_AUP_KAOSW_2_1_C0UW4A	0	0	0
	Darmor_AUP_LAOSW_3_1_C0UW4AC	0	0	0
	Darmor_AUP_MAOSW_4_1_C0UW4A	0	0	0
	Darmor_AUP_NAOSW_5_1_C0UW4A	0	0	0
	Darmor_AUP_PAOSW_6_1_C0UW4AC	0	0	0
	Ningyou_ERR037335	0	0	0
	TN04_ERR037297	0	0	0
	TN05_ERR037298	0	0	0
	TN08_ERR037301	0	0	0
	TN103_ERR037317	0	0	0
	TN108_ERR037318	0	0	0
	TN109_ERR037319	0	0	0
	TN10_ERR037294	0	0	0
	TN121_ERR037320	0	0	0
	TN124_ERR037321	0	0	0

	TN126_ERR037322	0	0	0
	TN128_ERR037323	0	0	0
	TN129_ERR037324	0	0	0
	TN12_ERR037302	0	0	0
	TN138_ERR037325	0	0	0
	TN149_ERR037326	0	0	0
	TN161_ERR037327	0	0	0
	TN163_ERR037328	0	0	0
	TN170_ERR037329	0	0	0
	TN171_ERR037332	0	0	0
	TN172_ERR037333	0	0	0
	TN176_ERR037330	0	0	0
	TN177_ERR037331	0	0	0
	TN24_ERR037299	0	0	0
	TN28_ERR037295	0	0	0
	TN30_ERR037305	0	0	0
	TN31_ERR037303	0	0	0
	TN38_ERR037304	0	0	0
	TN44_ERR037306	0	0	0
	TN48_ERR037307	0	0	0
	TN51_ERR037308	0	0	0
	TN57_ERR037309	0	0	0
	TN72_ERR037312	0	0	0
	TN76_ERR037311	0	0	0
	TN77_ERR037310	0	0	0
	TN85_ERR037315	0	0	0
	TN88_ERR037313	0	0	0
	TN90_ERR037316	0	0	0
	Tapidor_ERR037334	0	0	0
BnaA01g31800.1D2	Darmor_AUP_AOSW_2_D09BTACXX.I	0	3.08E-39	0
	Darmor_AUP_BOSW_3_D09BTACXX.I	0	2.37E-32	0
	Darmor_AUP_DOSW_2_D09BTACXX.I	0	3.78E-38	0
	Darmor_AUP_FOSW_4_D09BTACXX.I	0	0.006880779	0

	Darmor_AUP_HOSW_5_D09BTACXX.I	0	0.2687108	0
	Darmor_AUP_IAOSW_1_1_C0UW4A(	0	0	0
	Darmor_AUP_KAOSW_2_1_C0UW4A	0	0	0
	Darmor_AUP_LAOSW_3_1_C0UW4A(	0	0	0
	Darmor_AUP_MAOSW_4_1_C0UW4A	0	0.002769109	0
	Darmor_AUP_NAOSW_5_1_C0UW4A	0	0	0
	Darmor_AUP_PAOSW_6_1_C0UW4A(	0	1.18E-48	0
	Ningyou_ERR037335	0	1.88E-06	0
	TN04_ERR037297	0	3.18E-43	0
	TN05_ERR037298	0	3.00E-35	0
	TN08_ERR037301	0	0	0
	TN103_ERR037317	0	2.64E-32	0
	TN108_ERR037318	0	3.07E-28	0
	TN109_ERR037319	0	0	0
	TN10_ERR037294	0	1.32E-37	0
	TN121_ERR037320	0	5.98E-33	0
	TN124_ERR037321	0	3.05E-09	0
	TN126_ERR037322	0	8.86E-34	0
	TN128_ERR037323	0	0	0
	TN129_ERR037324	0	2.47E-25	0
	TN12_ERR037302	0	1.90E-31	0
	TN138_ERR037325	0	6.41E-32	0
	TN149_ERR037326	0	4.88E-27	0
	TN161_ERR037327	0	2.02E-31	0
	TN163_ERR037328	0	2.35E-27	0
	TN170_ERR037329	0	1.25E-09	0
	TN171_ERR037332	0	4.61E-35	0
	TN172_ERR037333	0	6.79E-40	0
	TN176_ERR037330	0	1.63E-45	0
	TN177_ERR037331	0	6.50E-06	0
	TN24_ERR037299	0	1.84E-25	0
	TN28_ERR037295	0	2.82E-33	0
	TN30_ERR037305	0	3.71E-31	0

	TN31_ERR037303	0	4.06E-31	0
	TN38_ERR037304	0	2.80E-35	0
	TN44_ERR037306	0	0.001789722	0
	TN48_ERR037307	0	1.80E-29	0
	TN51_ERR037308	0	1.28E-27	0
	TN57_ERR037309	0	1.29E-32	0
	TN72_ERR037312	0	5.76E-32	0
	TN76_ERR037311	0	7.32E-33	0
	TN77_ERR037310	0	1.47E-41	0
	TN85_ERR037315	0	0	0
	TN88_ERR037313	0	8.18E-33	0
	TN90_ERR037316	0	0	0
	Tapidor_ERR037334	0	0.00150621	0
BnaC02g07440.1D2	Darmor_AUP_AOSW_2_D09BTACXX.I	688	28.73316	27.28556
	Darmor_AUP_BOSW_3_D09BTACXX.I	449	20.42459	19.198
	Darmor_AUP_DOSW_2_D09BTACXX.I	1020	34.38396	32.73035
	Darmor_AUP_FOSW_4_D09BTACXX.II	226	9.405085	8.212166
	Darmor_AUP_HOSW_5_D09BTACXX.I	282	18.48946	16.62539
	Darmor_AUP_IAOSW_1_1_C0UW4AC	1704	26.70795	26.09219
	Darmor_AUP_KAOSW_2_1_C0UW4A	2201	34.99242	34.69817
	Darmor_AUP_LAOSW_3_1_C0UW4AC	525	8.006359	7.978017
	Darmor_AUP_MAOSW_4_1_C0UW4A	560	8.406944	8.067442
	Darmor_AUP_NAOSW_5_1_C0UW4A	516	7.666226	7.277939
	Darmor_AUP_PAOSW_6_1_C0UW4AC	2119	32.61775	32.4285
	Ningyou_ERR037335	0	4.01E-27	0
	TN04_ERR037297	0	2.02E-18	0
	TN05_ERR037298	0	7.39E-26	0
	TN08_ERR037301	0	8.43E-29	0
	TN103_ERR037317	0	2.05E-23	0
	TN108_ERR037318	0	4.33E-26	0
	TN109_ERR037319	0	4.79E-26	0
	TN10_ERR037294	1	0.3218429	0.3052417
	TN121_ERR037320	0	1.05E-28	0

	TN124_ERR037321	0	2.28E-29	0
	TN126_ERR037322	0	5.17E-29	0
	TN128_ERR037323	0	9.22E-19	0
	TN129_ERR037324	0	3.00E-29	0
	TN12_ERR037302	0	2.19E-27	0
	TN138_ERR037325	0	1.87E-24	0
	TN149_ERR037326	0	3.25E-28	0
	TN161_ERR037327	0	5.23E-28	0
	TN163_ERR037328	0	1.52E-28	0
	TN170_ERR037329	0	3.68E-28	0
	TN171_ERR037332	0	8.63E-19	0
	TN172_ERR037333	0	6.88E-29	0
	TN176_ERR037330	0	2.56E-29	0
	TN177_ERR037331	0	1.86E-22	0
	TN24_ERR037299	0	4.70E-26	0
	TN28_ERR037295	0	1.71E-17	0
	TN30_ERR037305	0	2.27E-28	0
	TN31_ERR037303	0	1.46E-28	0
	TN38_ERR037304	0	1.49E-28	0
	TN44_ERR037306	0	2.33E-19	0
	TN48_ERR037307	0	5.56E-28	0
	TN51_ERR037308	0	2.62E-21	0
	TN57_ERR037309	0	2.94E-29	0
	TN72_ERR037312	0	2.68E-28	0
	TN76_ERR037311	0	3.44E-29	0
	TN77_ERR037310	1	0.0481669	0.04816006
	TN85_ERR037315	0	1.07E-22	0
	TN88_ERR037313	0	4.85E-24	0
	TN90_ERR037316	0	2.08E-27	0
	Tapidor_ERR037334	0	6.86E-18	0
BnaU01g02910.1D2	Darmor_AUP_AOSW_2_D09BTACXX.I	129	2.648612	2.111126
	Darmor_AUP_BOSW_3_D09BTACXX.I	102	2.920173	2.497056
	Darmor_AUP_DOSW_2_D09BTACXX.I	57	0.8911423	0.7487545

	Darmor_AUP_FOSW_4_D09BTACXX.II	148	2.629316	2.187566
	Darmor_AUP_HOSW_5_D09BTACXX.I	65	1.311521	0.9998195
	Darmor_AUP_IAOSW_1_1_C0UW4AC	426	2.786886	2.570694
	Darmor_AUP_KAOSW_2_1_C0UW4A	329	2.230232	2.193839
	Darmor_AUP_LAOSW_3_1_C0UW4AC	188	1.295834	1.155047
	Darmor_AUP_MAOSW_4_1_C0UW4A	285	1.933336	1.856321
	Darmor_AUP_NAOSW_5_1_C0UW4A	125	0.8442447	0.8344967
	Darmor_AUP_PAOSW_6_1_C0UW4AC	359	2.510041	2.487225
	Ningyou_ERR037335	8	0.4367525	0.02603342
	TN04_ERR037297	5	0.111757	0.07584102
	TN05_ERR037298	15	0.3488656	0.06888059
	TN08_ERR037301	22	0.5142411	0.5133893
	TN103_ERR037317	8	0.2290059	0.2114753
	TN108_ERR037318	7	0.2229485	0.04665362
	TN109_ERR037319	12	0.2815234	0.2815234
	TN10_ERR037294	8	0.2655015	0.259222
	TN121_ERR037320	9	0.2496627	0.2496599
	TN124_ERR037321	5	0.2899767	0.00196351
	TN126_ERR037322	3	0.07563627	0.07563626
	TN128_ERR037323	0	3.62E-31	0
	TN129_ERR037324	19	0.4811413	0.4803872
	TN12_ERR037302	8	0.2676084	0.2673929
	TN138_ERR037325	5	0.1752341	0.03805422
	TN149_ERR037326	3	0.1731312	0.1731312
	TN161_ERR037327	2	0.05518912	0.05413949
	TN163_ERR037328	3	0.09273186	0
	TN170_ERR037329	13	0.3144709	0.1749035
	TN171_ERR037332	0	1.30E-29	0
	TN172_ERR037333	14	0.3147303	0.3033109
	TN176_ERR037330	15	0.362101	0.1947422
	TN177_ERR037331	7	0.1479307	0.04355149
	TN24_ERR037299	0	4.94E-08	0
	TN28_ERR037295	18	0.3976036	0.3955039

	TN30_ERR037305	6	0.1271274	0.04041649
	TN31_ERR037303	22	0.5701708	0.5686103
	TN38_ERR037304	11	0.2443323	0.0594753
	TN44_ERR037306	3	0.1095679	0.1089963
	TN48_ERR037307	4	0.1850465	0.1850465
	TN51_ERR037308	4	0.1066263	0.1065834
	TN57_ERR037309	4	0.08059197	0.03878011
	TN72_ERR037312	6	0.14494	0.1449093
	TN76_ERR037311	4	0.08646909	0.08130576
	TN77_ERR037310	0	4.68E-13	0
	TN85_ERR037315	3	0.0707646	0.06790331
	TN88_ERR037313	1	0.04268454	0
	TN90_ERR037316	4	0.1753386	0.1752929
	Tapidor_ERR037334	1	0.02219195	0.02219195
BnaC01g20210.1D2	Darmor_AUP_AOSW_2_D09BTACXX.I	15	0.2250041	0.1369468
	Darmor_AUP_BOSW_3_D09BTACXX.I	27	0.4555211	0.3770889
	Darmor_AUP_DOSW_2_D09BTACXX.I	18	0.2444429	0.2429431
	Darmor_AUP_FOSW_4_D09BTACXX.II	436	6.227613	5.60556
	Darmor_AUP_HOSW_5_D09BTACXX.I	225	3.661206	3.385816
	Darmor_AUP_IAOSW_1_1_C0UW4A(	48	0.2530513	0.2510614
	Darmor_AUP_KAOSW_2_1_C0UW4A	62	0.3272138	0.3222841
	Darmor_AUP_LAOSW_3_1_C0UW4A(	1182	6.648739	6.457217
	Darmor_AUP_MAOSW_4_1_C0UW4A	1115	6.366319	6.184684
	Darmor_AUP_NAOSW_5_1_C0UW4A	1145	6.49648	6.355046
	Darmor_AUP_PAOSW_6_1_C0UW4A(	40	0.2176432	0.2171239
	Ningyou_ERR037335	228	5.403686	4.851384
	TN04_ERR037297	254	4.540869	4.104896
	TN05_ERR037298	330	6.561361	5.94908
	TN08_ERR037301	221	6.135084	5.364078
	TN103_ERR037317	163	4.89296	4.207774
	TN108_ERR037318	251	7.005306	6.338233
	TN109_ERR037319	0	3.55E-23	0
	TN10_ERR037294	327	6.89542	6.023824

	TN121_ERR037320	0	2.02E-24	0
	TN124_ERR037321	0	6.54E-27	0
	TN126_ERR037322	0	9.25E-25	0
	TN128_ERR037323	317	6.101769	5.471313
	TN129_ERR037324	331	6.121021	5.419633
	TN12_ERR037302	0	1.01E-26	0
	TN138_ERR037325	0	3.44E-25	0
	TN149_ERR037326	0	4.89E-24	0
	TN161_ERR037327	184	3.995544	3.595157
	TN163_ERR037328	340	7.724613	6.735977
	TN170_ERR037329	2	0.03070924	0.02224305
	TN171_ERR037332	197	4.39069	3.641121
	TN172_ERR037333	0	2.99E-27	0
	TN176_ERR037330	433	7.343542	6.449951
	TN177_ERR037331	2	0.03259273	0.03034251
	TN24_ERR037299	0	2.33E-25	0
	TN28_ERR037295	335	6.956862	6.463077
	TN30_ERR037305	259	5.448304	4.934539
	TN31_ERR037303	356	7.786603	7.011466
	TN38_ERR037304	1	0.04932599	0
	TN44_ERR037306	159	5.503551	4.510673
	TN48_ERR037307	0	9.40E-26	0
	TN51_ERR037308	0	2.01E-25	0
	TN57_ERR037309	343	6.096976	5.357071
	TN72_ERR037312	220	5.965939	5.187254
	TN76_ERR037311	257	6.199952	5.079072
	TN77_ERR037310	126	3.182838	2.660021
	TN85_ERR037315	340	7.629168	6.720445
	TN88_ERR037313	217	4.384685	3.742172
	TN90_ERR037316	333	8.823907	7.800239
	Tapidor_ERR037334	0	1.95E-23	0
BnaU01g04540.1D2	Darmor_AUP_AOSW_2_D09BTACXX.I	17	1.235115	1.235115
	Darmor_AUP_BOSW_3_D09BTACXX.I	8	0.7872366	0.7872366

	Darmor_AUP_DOSW_2_D09BTACXX.I	15	1.029523	1.0263
	Darmor_AUP_FOSW_4_D09BTACXX.II	4	0.3422307	0.3421916
	Darmor_AUP_HOSW_5_D09BTACXX.I	1	0.09876414	0.09876414
	Darmor_AUP_IAOSW_1_1_C0UW4AC	9	0.2951762	0.2951762
	Darmor_AUP_KAOSW_2_1_C0UW4A	10	0.3295639	0.3295639
	Darmor_AUP_LAOSW_3_1_C0UW4A(	7	0.2273112	0.2273112
	Darmor_AUP_MAOSW_4_1_C0UW4A	3	0.09757736	0.09757736
	Darmor_AUP_NAOSW_5_1_C0UW4A	7	0.2323525	0.2323525
	Darmor_AUP_PAOSW_6_1_C0UW4A(	6	0.1977747	0.1977747
	Ningyou_ERR037335	1	0.1227338	0.1211307
	TN04_ERR037297	0	0	0
	TN05_ERR037298	0	5.07E-54	0
	TN08_ERR037301	0	8.10E-38	0
	TN103_ERR037317	0	0	0
	TN108_ERR037318	0	0	0
	TN109_ERR037319	0	0	0
	TN10_ERR037294	0	4.16E-47	0
	TN121_ERR037320	0	1.28E-37	0
	TN124_ERR037321	0	0	0
	TN126_ERR037322	0	3.53E-35	0
	TN128_ERR037323	0	2.90E-36	0
	TN129_ERR037324	0	2.22E-29	0
	TN12_ERR037302	0	0	0
	TN138_ERR037325	0	0	0
	TN149_ERR037326	0	9.28E-43	0
	TN161_ERR037327	0	0	0
	TN163_ERR037328	0	0	0
	TN170_ERR037329	0	3.57E-28	0
	TN171_ERR037332	0	4.48E-61	0
	TN172_ERR037333	0	5.38E-26	0
	TN176_ERR037330	0	3.72E-08	3.72E-08
	TN177_ERR037331	0	5.59E-38	0
	TN24_ERR037299	0	4.08E-37	0

	TN28_ERR037295	0	2.97E-40	0
	TN30_ERR037305	0	3.95E-104	0
	TN31_ERR037303	0	2.92E-38	0
	TN38_ERR037304	0	1.54E-37	0
	TN44_ERR037306	0	8.97E-89	0
	TN48_ERR037307	0	2.45E-76	0
	TN51_ERR037308	0	1.89E-95	0
	TN57_ERR037309	0	0	0
	TN72_ERR037312	0	0	0
	TN76_ERR037311	0	0	0
	TN77_ERR037310	0	0	0
	TN85_ERR037315	0	0	0
	TN88_ERR037313	0	7.61E-25	0
	TN90_ERR037316	0	7.18E-35	0
	Tapidor_ERR037334	0	1.05E-38	0
BnaC02g06840.1D2	Darmor_AUP_AOSW_2_D09BTACXX.I	276	8.126687	7.260045
	Darmor_AUP_BOSW_3_D09BTACXX.I	152	6.165458	5.200896
	Darmor_AUP_DOSW_2_D09BTACXX.I	301	6.903671	5.939593
	Darmor_AUP_FOSW_4_D09BTACXX.II	148	4.156555	3.369988
	Darmor_AUP_HOSW_5_D09BTACXX.I	207	7.480783	6.583302
	Darmor_AUP_IAOSW_1_1_C0UW4AC	881	9.246281	8.7794
	Darmor_AUP_KAOSW_2_1_C0UW4A	844	8.897485	8.537705
	Darmor_AUP_LAOSW_3_1_C0UW4AC	275	2.764425	2.741172
	Darmor_AUP_MAOSW_4_1_C0UW4A	330	3.22195	3.106399
	Darmor_AUP_NAOSW_5_1_C0UW4A	217	2.124938	1.996868
	Darmor_AUP_PAOSW_6_1_C0UW4AC	902	10.39726	9.99293
	Ningyou_ERR037335	8	0.2837306	0
	TN04_ERR037297	0	1.49E-17	0
	TN05_ERR037298	3	0.09663195	0.08615074
	TN08_ERR037301	1	0.03276517	0.01621012
	TN103_ERR037317	3	0.1264259	0.09857012
	TN108_ERR037318	0	4.62E-16	0
	TN109_ERR037319	5	0.3432492	0.131208

	TN10_ERR037294	1	0.0287973	0	
	TN121_ERR037320	0	2.40E-19	0	
	TN124_ERR037321	1	0.06757777	0	
	TN126_ERR037322	2	0.05640097	0.04707046	
	TN128_ERR037323	1	0.09993491	0	
	TN129_ERR037324	3	0.08563074	0.06390204	
	TN12_ERR037302	0	4.38E-17	0	
	TN138_ERR037325	0	5.27E-20	0	
	TN149_ERR037326	0	2.68E-16	0	
	TN161_ERR037327	0	3.82E-23	0	
	TN163_ERR037328	0	5.63E-18	0	
	TN170_ERR037329	0	2.96E-23	0	
	TN171_ERR037332	1	0.02966624	0.01152549	
	TN172_ERR037333	3	0.1435296	0	
	TN176_ERR037330	0	8.57E-20	0	
	TN177_ERR037331	5	0.1507838	0.01267928	
	TN24_ERR037299	3	0.3061198	0.08895055	
	TN28_ERR037295	1	0.0282114	0.0135192	
	TN30_ERR037305	3	0.0930485	0.08292202	
	TN31_ERR037303	3	0.09759287	0.05435875	
	TN38_ERR037304	2	0.3553359	0.2505288	
	TN44_ERR037306	3	0.1339485	0	
	TN48_ERR037307	1	0.03341419	0.0291646	
	TN51_ERR037308	2	0.07723735	0.05819874	
	TN57_ERR037309	0	4.15E-24	0	
	TN72_ERR037312	2	0.07151741	0.06481924	
	TN76_ERR037311	3	0.09592705	0	
	TN77_ERR037310	0	8.27E-23	0	
	TN85_ERR037315	0	1.39E-23	0	
	TN88_ERR037313	0	1.04E-17	0	
	TN90_ERR037316	1	0.03224901	0	
	Tapidor_ERR037334	1	0.3151808	0	
BnaA03g42810.1D2	Darmor_AUP_AOSW_2_D09BTACXX.I	0	0	0	

	Darmor_AUP_BOSW_3_D09BTACXX.I	0	0	0
	Darmor_AUP_DOSW_2_D09BTACXX.I	0	0	0
	Darmor_AUP_FOSW_4_D09BTACXX.II	0	0	0
	Darmor_AUP_HOSW_5_D09BTACXX.I	0	0	0
	Darmor_AUP_IAOSW_1_1_C0UW4A(	0	0	0
	Darmor_AUP_KAOSW_2_1_C0UW4A	0	0	0
	Darmor_AUP_LAOSW_3_1_C0UW4A(	0	0	0
	Darmor_AUP_MAOSW_4_1_C0UW4/	0	0	0
	Darmor_AUP_NAOSW_5_1_C0UW4A	0	0	0
	Darmor_AUP_PAOSW_6_1_C0UW4A(	1	0.09500131	0.09500131
	Ningyou_ERR037335	0	0	0
	TN04_ERR037297	0	0	0
	TN05_ERR037298	0	0	0
	TN08_ERR037301	0	0	0
	TN103_ERR037317	0	0	0
	TN108_ERR037318	0	0	0
	TN109_ERR037319	0	0	0
	TN10_ERR037294	0	0	0
	TN121_ERR037320	0	0	0
	TN124_ERR037321	0	0	0
	TN126_ERR037322	0	0	0
	TN128_ERR037323	0	0	0
	TN129_ERR037324	0	0	0
	TN12_ERR037302	0	0	0
	TN138_ERR037325	0	0	0
	TN149_ERR037326	0	0	0
	TN161_ERR037327	0	0	0
	TN163_ERR037328	0	0	0
	TN170_ERR037329	0	0	0
	TN171_ERR037332	0	0	0
	TN172_ERR037333	0	0	0
	TN176_ERR037330	0	0	0
	TN177_ERR037331	0	0	0

	TN24_ERR037299	0	0	0
	TN28_ERR037295	0	0	0
	TN30_ERR037305	0	0	0
	TN31_ERR037303	0	0	0
	TN38_ERR037304	0	0	0
	TN44_ERR037306	0	0	0
	TN48_ERR037307	0	0	0
	TN51_ERR037308	0	0	0
	TN57_ERR037309	0	0	0
	TN72_ERR037312	0	0	0
	TN76_ERR037311	0	0	0
	TN77_ERR037310	0	0	0
	TN85_ERR037315	0	0	0
	TN88_ERR037313	0	0	0
	TN90_ERR037316	0	0	0
	Tapidor_ERR037334	0	0	0
BnaC05g10000.1D2	Darmor_AUP_AOSW_2_D09BTACXX.I	0	0	0
	Darmor_AUP_BOSW_3_D09BTACXX.I	0	0	0
	Darmor_AUP_DOSW_2_D09BTACXX.I	0	0	0
	Darmor_AUP_FOSW_4_D09BTACXX.II	0	0	0
	Darmor_AUP_HOSW_5_D09BTACXX.I	0	0	0
	Darmor_AUP_IAOSW_1_1_C0UW4AC	0	0	0
	Darmor_AUP_KAOSW_2_1_C0UW4A	0	0	0
	Darmor_AUP_LAOSW_3_1_C0UW4AC	0	0	0
	Darmor_AUP_MAOSW_4_1_C0UW4A	0	0	0
	Darmor_AUP_NAOSW_5_1_C0UW4A	0	0	0
	Darmor_AUP_PAOSW_6_1_C0UW4AC	0	0	0
	Ningyou_ERR037335	0	4.75E-40	0
	TN04_ERR037297	0	0	0
	TN05_ERR037298	0	0	0
	TN08_ERR037301	0	0	0
	TN103_ERR037317	0	0	0
	TN108_ERR037318	0	0	0

	TN109_ERR037319	0	0	0
	TN10_ERR037294	0	0	0
	TN121_ERR037320	0	0	0
	TN124_ERR037321	0	0	0
	TN126_ERR037322	0	0	0
	TN128_ERR037323	0	0	0
	TN129_ERR037324	0	0	0
	TN12_ERR037302	0	0	0
	TN138_ERR037325	0	0	0
	TN149_ERR037326	0	0	0
	TN161_ERR037327	0	0	0
	TN163_ERR037328	0	0	0
	TN170_ERR037329	0	0	0
	TN171_ERR037332	0	0	0
	TN172_ERR037333	0	0	0
	TN176_ERR037330	0	0	0
	TN177_ERR037331	0	0	0
	TN24_ERR037299	0	0	0
	TN28_ERR037295	0	0	0
	TN30_ERR037305	0	0	0
	TN31_ERR037303	0	0	0
	TN38_ERR037304	0	0	0
	TN44_ERR037306	0	0	0
	TN48_ERR037307	0	0	0
	TN51_ERR037308	0	0	0
	TN57_ERR037309	0	0	0
	TN72_ERR037312	0	0	0
	TN76_ERR037311	0	0	0
	TN77_ERR037310	0	0	0
	TN85_ERR037315	0	0	0
	TN88_ERR037313	0	0	0
	TN90_ERR037316	0	0	0
	Tapidor_ERR037334	0	0	0

BnaC03g12730.1D2	Darmor_AUP_AOSW_2_D09BTACXX.I	80	27.68174	27.68174
	Darmor_AUP_BOSW_3_D09BTACXX.I	82	38.36585	38.36585
	Darmor_AUP_DOSW_2_D09BTACXX.I	116	36.95063	0
	Darmor_AUP_FOSW_4_D09BTACXX.II	59	24.08864	24.08864
	Darmor_AUP_HOSW_5_D09BTACXX.I	45	20.73745	20.73745
	Darmor_AUP_IAOSW_1_1_C0UW4AC	172	22.6368	22.6368
	Darmor_AUP_KAOSW_2_1_C0UW4A	111	14.91014	14.91014
	Darmor_AUP_LAOSW_3_1_C0UW4AC	69	9.887364	9.887364
	Darmor_AUP_MAOSW_4_1_C0UW4A	81	11.69283	11.69283
	Darmor_AUP_NAOSW_5_1_C0UW4A	82	11.98611	11.98611
	Darmor_AUP_PAOSW_6_1_C0UW4AC	117	16.39936	16.39936
	Ningyou_ERR037335	22	15.10124	15.08074
	TN04_ERR037297	13	7.274361	7.274361
	TN05_ERR037298	0	0	0
	TN08_ERR037301	0	0	0
	TN103_ERR037317	28	19.65389	17.35098
	TN108_ERR037318	0	0	0
	TN109_ERR037319	0	0	0
	TN10_ERR037294	0	0	0
	TN121_ERR037320	0	0	0
	TN124_ERR037321	32	16.34381	15.15343
	TN126_ERR037322	23	11.17062	11.04965
	TN128_ERR037323	17	10.42592	9.776068
	TN129_ERR037324	0	0	0
	TN12_ERR037302	0	0	0
	TN138_ERR037325	0	0	0
	TN149_ERR037326	0	0	0
	TN161_ERR037327	14	9.61158	9.61158
	TN163_ERR037328	15	8.768442	8.768442
	TN170_ERR037329	0	0	0
	TN171_ERR037332	0	0	0
	TN172_ERR037333	0	0	0
	TN176_ERR037330	27	15.62398	12.03961

	TN177_ERR037331	21	12.51947	3.765088
	TN24_ERR037299	0	0	0
	TN28_ERR037295	0	0	0
	TN30_ERR037305	0	0	0
	TN31_ERR037303	0	0	0
	TN38_ERR037304	0	1.36E-35	0
	TN44_ERR037306	0	0	0
	TN48_ERR037307	0	0	0
	TN51_ERR037308	14	9.739478	8.050255
	TN57_ERR037309	24	11.59059	11.59059
	TN72_ERR037312	0	0	0
	TN76_ERR037311	0	0	0
	TN77_ERR037310	1	0.5588834	0.5588834
	TN85_ERR037315	16	10.24044	10.20701
	TN88_ERR037313	0	0	0
	TN90_ERR037316	0	0	0
	Tapidor_ERR037334	0	0	0
BnaC06g20070.1D2	Darmor_AUP_AOSW_2_D09BTACXX.I	50	1.788112	1.788112
	Darmor_AUP_BOSW_3_D09BTACXX.I	98	4.67085	4.67085
	Darmor_AUP_DOSW_2_D09BTACXX.I	1	0.03273471	0.03273471
	Darmor_AUP_FOSW_4_D09BTACXX.II	6	0.2494773	0.2494773
	Darmor_AUP_HOSW_5_D09BTACXX.I	5	0.2431672	0.2431672
	Darmor_AUP_IAOSW_1_1_C0UW4AC	128	1.754557	1.754557
	Darmor_AUP_KAOSW_2_1_C0UW4A	88	1.235919	1.235919
	Darmor_AUP_LAOSW_3_1_C0UW4A(	3	0.04440506	0.04440506
	Darmor_AUP_MAOSW_4_1_C0UW4A	4	0.05931307	0.05931307
	Darmor_AUP_NAOSW_5_1_C0UW4A	0	0	0
	Darmor_AUP_PAOSW_6_1_C0UW4A(	77	1.144247	1.144247
	Ningyou_ERR037335	0	0	0
	TN04_ERR037297	0	0	0
	TN05_ERR037298	0	0	0
	TN08_ERR037301	2	0.1034497	0.1034497
	TN103_ERR037317	1	0.07084282	0.07084282

	TN108_ERR037318	0	0	0
	TN109_ERR037319	0	0	0
	TN10_ERR037294	2	0.08949739	0.08949739
	TN121_ERR037320	0	0	0
	TN124_ERR037321	1	0.04851165	0.04851165
	TN126_ERR037322	1	0.04972901	0.04972901
	TN128_ERR037323	0	0	0
	TN129_ERR037324	1	0.0489866	0.0489866
	TN12_ERR037302	0	0	0
	TN138_ERR037325	1	0.06604351	0.06604351
	TN149_ERR037326	1	0.07066862	0.07066862
	TN161_ERR037327	0	0	0
	TN163_ERR037328	2	0.1176629	0.1176629
	TN170_ERR037329	0	0	0
	TN171_ERR037332	1	0.04548245	0.04548245
	TN172_ERR037333	0	0	0
	TN176_ERR037330	0	0	0
	TN177_ERR037331	0	0	0
	TN24_ERR037299	2	0.1328223	0.1328223
	TN28_ERR037295	0	0	0
	TN30_ERR037305	0	0	0
	TN31_ERR037303	0	0	0
	TN38_ERR037304	0	0	0
	TN44_ERR037306	1	0.07023049	0.07023049
	TN48_ERR037307	0	0	0
	TN51_ERR037308	0	0	0
	TN57_ERR037309	0	0	0
	TN72_ERR037312	0	0	0
	TN76_ERR037311	0	0	0
	TN77_ERR037310	0	0	0
	TN85_ERR037315	1	0.05930163	0.05930163
	TN88_ERR037313	0	0	0
	TN90_ERR037316	1	0.05277146	0.05277146

	Tapidor_ERR037334	0	0	0
BnaC03g02810.1D2	Darmor_AUP_AOSW_2_D09BTACXX.I	147	5.772974	5.772974
	Darmor_AUP_BOSW_3_D09BTACXX.I	272	14.18994	14.1899
	Darmor_AUP_DOSW_2_D09BTACXX.I	9	0.3132219	0.3132219
	Darmor_AUP_FOSW_4_D09BTACXX.I	2	0.08823061	0.08823061
	Darmor_AUP_HOSW_5_D09BTACXX.I	46	2.461899	2.461899
	Darmor_AUP_IAOSW_1_1_C0UW4A(	1121	17.20211	17.20211
	Darmor_AUP_KAOSW_2_1_C0UW4A	398	6.188707	6.188707
	Darmor_AUP_LAOSW_3_1_C0UW4A(	3	0.0490202	0.0490202
	Darmor_AUP_MAOSW_4_1_C0UW4/	1	0.01640374	0.01640374
	Darmor_AUP_NAOSW_5_1_C0UW4A	2	0.03261088	0.03261088
	Darmor_AUP_PAOSW_6_1_C0UW4A(	940	15.385	15.385
	Ningyou_ERR037335	1	0.06557848	0.06557204
	TN04_ERR037297	0	5.26E-45	0
	TN05_ERR037298	0	0	0
	TN08_ERR037301	0	0	0
	TN103_ERR037317	0	6.57E-30	0
	TN108_ERR037318	0	9.80E-31	0
	TN109_ERR037319	0	0	0
	TN10_ERR037294	0	2.14E-35	0
	TN121_ERR037320	0	6.23E-42	0
	TN124_ERR037321	0	9.92E-63	0
	TN126_ERR037322	0	1.13E-29	0
	TN128_ERR037323	0	2.98E-32	0
	TN129_ERR037324	0	7.20E-32	0
	TN12_ERR037302	0	2.34E-29	0
	TN138_ERR037325	0	1.17E-28	0
	TN149_ERR037326	0	0	0
	TN161_ERR037327	0	0	0
	TN163_ERR037328	4	0.2582177	0.2582177
	TN170_ERR037329	0	3.19E-32	0
	TN171_ERR037332	0	4.94E-28	0
	TN172_ERR037333	0	6.88E-66	0

	TN176_ERR037330	0	3.22E-32	0
	TN177_ERR037331	0	2.63E-43	0
	TN24_ERR037299	0	1.20E-26	0
	TN28_ERR037295	0	2.19E-35	0
	TN30_ERR037305	0	2.24E-06	0
	TN31_ERR037303	0	1.14E-12	0
	TN38_ERR037304	0	0	0
	TN44_ERR037306	0	8.45E-41	0
	TN48_ERR037307	0	0	0
	TN51_ERR037308	1	0.06747015	0.06747015
	TN57_ERR037309	1	0.05318477	0.05317836
	TN72_ERR037312	0	0	0
	TN76_ERR037311	0	2.52E-31	0
	TN77_ERR037310	3	0.1672957	0.1672877
	TN85_ERR037315	0	5.95E-31	0
	TN88_ERR037313	0	1.67E-26	0
	TN90_ERR037316	0	9.07E-54	0
	Tapidor_ERR037334	0	3.32E-35	0
BnaC04g25350.1D2	Darmor_AUP_AOSW_2_D09BTACXX.I	0	0	0
	Darmor_AUP_BOSW_3_D09BTACXX.I	0	0	0
	Darmor_AUP_DOSW_2_D09BTACXX.I	0	0	0
	Darmor_AUP_FOSW_4_D09BTACXX.II	0	0	0
	Darmor_AUP_HOSW_5_D09BTACXX.I	0	0	0
	Darmor_AUP_IAOSW_1_1_C0UW4AC	0	0	0
	Darmor_AUP_KAOSW_2_1_C0UW4A	0	0	0
	Darmor_AUP_LAOSW_3_1_C0UW4AC	0	0	0
	Darmor_AUP_MAOSW_4_1_C0UW4A	0	0	0
	Darmor_AUP_NAOSW_5_1_C0UW4A	0	0	0
	Darmor_AUP_PAOSW_6_1_C0UW4AC	0	0	0
	Ningyou_ERR037335	0	0	0
	TN04_ERR037297	0	0	0
	TN05_ERR037298	0	0	0
	TN08_ERR037301	0	0	0

	TN103_ERR037317	0	0	0
	TN108_ERR037318	0	0	0
	TN109_ERR037319	0	0	0
	TN10_ERR037294	0	0	0
	TN121_ERR037320	0	0	0
	TN124_ERR037321	0	0	0
	TN126_ERR037322	0	0	0
	TN128_ERR037323	0	0	0
	TN129_ERR037324	0	0	0
	TN12_ERR037302	0	0	0
	TN138_ERR037325	0	0	0
	TN149_ERR037326	0	0	0
	TN161_ERR037327	0	0	0
	TN163_ERR037328	0	0	0
	TN170_ERR037329	0	0	0
	TN171_ERR037332	0	0	0
	TN172_ERR037333	0	0	0
	TN176_ERR037330	0	0	0
	TN177_ERR037331	0	0	0
	TN24_ERR037299	0	0	0
	TN28_ERR037295	0	0	0
	TN30_ERR037305	0	0	0
	TN31_ERR037303	0	0	0
	TN38_ERR037304	0	0	0
	TN44_ERR037306	0	0	0
	TN48_ERR037307	0	0	0
	TN51_ERR037308	0	0	0
	TN57_ERR037309	0	0	0
	TN72_ERR037312	0	0	0
	TN76_ERR037311	0	0	0
	TN77_ERR037310	0	0	0
	TN85_ERR037315	0	0	0
	TN88_ERR037313	0	0	0

	TN90_ERR037316	0	0	0
	Tapidor_ERR037334	0	0	0
BnaC01g07850.1D2	Darmor_AUP_AOSW_2_D09BTACXX.I	0	0	0
	Darmor_AUP_BOSW_3_D09BTACXX.I	0	0	0
	Darmor_AUP_DOSW_2_D09BTACXX.I	0	0	0
	Darmor_AUP_FOSW_4_D09BTACXX.II	0	0	0
	Darmor_AUP_HOSW_5_D09BTACXX.I	13	0.6777737	0.6777737
	Darmor_AUP_IAOSW_1_1_C0UW4AC	0	0	0
	Darmor_AUP_KAOSW_2_1_C0UW4A	0	0	0
	Darmor_AUP_LAOSW_3_1_C0UW4AC	0	0	0
	Darmor_AUP_MAOSW_4_1_C0UW4A	0	0	0
	Darmor_AUP_NAOSW_5_1_C0UW4A	0	0	0
	Darmor_AUP_PAOSW_6_1_C0UW4AC	0	0	0
	Ningyou_ERR037335	0	0	0
	TN04_ERR037297	0	0	0
	TN05_ERR037298	0	0	0
	TN08_ERR037301	0	0	0
	TN103_ERR037317	0	0	0
	TN108_ERR037318	1	0.07039784	0.07039784
	TN109_ERR037319	0	0	0
	TN10_ERR037294	0	0	0
	TN121_ERR037320	0	0	0
	TN124_ERR037321	0	0	0
	TN126_ERR037322	0	0	0
	TN128_ERR037323	0	0	0
	TN129_ERR037324	0	0	0
	TN12_ERR037302	0	0	0
	TN138_ERR037325	0	0	0
	TN149_ERR037326	0	0	0
	TN161_ERR037327	0	0	0
	TN163_ERR037328	0	0	0
	TN170_ERR037329	0	0	0
	TN171_ERR037332	0	0	0

	TN172_ERR037333	0	0	0
	TN176_ERR037330	0	0	0
	TN177_ERR037331	0	0	0
	TN24_ERR037299	0	0	0
	TN28_ERR037295	0	0	0
	TN30_ERR037305	0	0	0
	TN31_ERR037303	0	0	0
	TN38_ERR037304	0	0	0
	TN44_ERR037306	0	0	0
	TN48_ERR037307	0	0	0
	TN51_ERR037308	0	0	0
	TN57_ERR037309	0	0	0
	TN72_ERR037312	0	0	0
	TN76_ERR037311	0	0	0
	TN77_ERR037310	0	0	0
	TN85_ERR037315	0	0	0
	TN88_ERR037313	0	0	0
	TN90_ERR037316	0	0	0
	Tapidor_ERR037334	0	0	0
BnaA09g47180.1D2	Darmor_AUP_AOSW_2_D09BTACXX.I	3	0.1011155	0.1011155
	Darmor_AUP_BOSW_3_D09BTACXX.I	0	1.67E-23	0
	Darmor_AUP_DOSW_2_D09BTACXX.I	0	3.23E-32	0
	Darmor_AUP_FOSW_4_D09BTACXX.II	10	0.3685965	0.367477
	Darmor_AUP_HOSW_5_D09BTACXX.I	26	1.983066	1.177698
	Darmor_AUP_IAOSW_1_1_C0UW4A(	9	0.1249431	0.1085491
	Darmor_AUP_KAOSW_2_1_C0UW4A	1	0.02788435	0.02788435
	Darmor_AUP_LAOSW_3_1_C0UW4A(	15	0.2291779	0.2240149
	Darmor_AUP_MAOSW_4_1_C0UW4A	8	0.1112147	0.1111799
	Darmor_AUP_NAOSW_5_1_C0UW4A	24	0.3354243	0.295622
	Darmor_AUP_PAOSW_6_1_C0UW4A(	7	0.09976651	0.09976651
	Ningyou_ERR037335	9	0.5308244	0.3943103
	TN04_ERR037297	0	5.19E-24	0
	TN05_ERR037298	0	1.21E-29	0

	TN08_ERR037301	0	0	0
	TN103_ERR037317	6	0.7287038	0.6978266
	TN108_ERR037318	0	1.17E-24	0
	TN109_ERR037319	6	0.3307485	0.3307295
	TN10_ERR037294	0	3.60E-24	0
	TN121_ERR037320	4	0.2647848	0.2647846
	TN124_ERR037321	0	1.13E-35	0
	TN126_ERR037322	12	0.9169544	0.8191904
	TN128_ERR037323	10	0.8111516	0.8088771
	TN129_ERR037324	4	0.3665519	0.3158848
	TN12_ERR037302	7	0.3348823	0.3348823
	TN138_ERR037325	1	0.3137462	0.3136807
	TN149_ERR037326	0	2.10E-28	0
	TN161_ERR037327	9	0.8965968	0
	TN163_ERR037328	19	1.457373	1.275775
	TN170_ERR037329	0	4.93E-12	0
	TN171_ERR037332	0	0	0
	TN172_ERR037333	0	2.43E-25	0
	TN176_ERR037330	2	0.08546523	0.08546523
	TN177_ERR037331	0	3.84E-29	0
	TN24_ERR037299	0	6.69E-19	0
	TN28_ERR037295	5	0.3599846	0.3599845
	TN30_ERR037305	0	1.26E-22	0
	TN31_ERR037303	0	1.95E-26	0
	TN38_ERR037304	0	0	0
	TN44_ERR037306	0	1.05E-06	0
	TN48_ERR037307	7	0.3578088	0.1473578
	TN51_ERR037308	13	1.015857	0.8934591
	TN57_ERR037309	20	1.052525	0.1856929
	TN72_ERR037312	9	1.070697	0.9979253
	TN76_ERR037311	3	0.3038208	0.3038018
	TN77_ERR037310	0	1.45E-05	1.43E-05
	TN85_ERR037315	10	1.160184	1.152741

	TN88_ERR037313	0	7.20E-23	0
	TN90_ERR037316	8	0.6557161	0.2066004
	Tapidor_ERR037334	0	3.63E-29	0
BnaC02g06050.1D2	Darmor_AUP_AOSW_2_D09BTACXX.I	9	0.5286093	0
	Darmor_AUP_BOSW_3_D09BTACXX.I	5	0.3829976	0.369711
	Darmor_AUP_DOSW_2_D09BTACXX.I	53	5.900912	4.066256
	Darmor_AUP_FOSW_4_D09BTACXX.II	16	6.271788	3.1655
	Darmor_AUP_HOSW_5_D09BTACXX.I	92	18.6113	12.97481
	Darmor_AUP_IAOSW_1_1_C0UW4A(	10	0.4814103	0.4810262
	Darmor_AUP_KAOSW_2_1_C0UW4A	32	1.381361	0.976919
	Darmor_AUP_LAOSW_3_1_C0UW4A(	151	3.081857	2.683834
	Darmor_AUP_MAOSW_4_1_C0UW4/	147	3.759365	2.952749
	Darmor_AUP_NAOSW_5_1_C0UW4A	73	1.633236	1.483566
	Darmor_AUP_PAOSW_6_1_C0UW4A(	17	0.3050093	0.2543395
	Ningyou_ERR037335	0	1.14E-09	0
	TN04_ERR037297	0	2.69E-09	0
	TN05_ERR037298	0	1.21E-08	0
	TN08_ERR037301	0	2.32E-09	0
	TN103_ERR037317	0	0.08103396	0.08103396
	TN108_ERR037318	0	0	0
	TN109_ERR037319	0	0	0
	TN10_ERR037294	0	0.05015654	0.0501562
	TN121_ERR037320	0	2.35E-12	0
	TN124_ERR037321	0	5.18E-09	0
	TN126_ERR037322	0	6.02E-27	0
	TN128_ERR037323	0	2.52E-06	0
	TN129_ERR037324	0	4.59E-29	0
	TN12_ERR037302	0	4.17E-09	0
	TN138_ERR037325	0	2.67E-31	0
	TN149_ERR037326	0	1.89E-16	0
	TN161_ERR037327	0	8.44E-11	0
	TN163_ERR037328	0	0	0
	TN170_ERR037329	0	8.69E-33	0

	TN171_ERR037332	0	0.04174659	0.03817887
	TN172_ERR037333	0	3.60E-08	0
	TN176_ERR037330	0	2.88E-27	0
	TN177_ERR037331	0	4.68E-12	0
	TN24_ERR037299	0	1.17E-17	0
	TN28_ERR037295	0	3.08E-29	0
	TN30_ERR037305	1	0.0775402	0.05462455
	TN31_ERR037303	0	3.80E-22	0
	TN38_ERR037304	0	0.008428885	0
	TN44_ERR037306	0	0.2358055	0.2358055
	TN48_ERR037307	0	7.76E-13	0
	TN51_ERR037308	0	0	0
	TN57_ERR037309	0	2.04E-28	0
	TN72_ERR037312	0	4.99E-18	0
	TN76_ERR037311	0	0.000185347	0.0001831165
	TN77_ERR037310	0	2.74E-14	0
	TN85_ERR037315	0	3.18E-25	0
	TN88_ERR037313	0	5.54E-12	0
	TN90_ERR037316	0	3.52E-07	0
	Tapidor_ERR037334	0	0.02762287	0.02511343
BnaC09g10800.1D2	Darmor_AUP_AOSW_2_D09BTACXX.I	373	39.80954	34.29618
	Darmor_AUP_BOSW_3_D09BTACXX.I	276	39.69768	33.49211
	Darmor_AUP_DOSW_2_D09BTACXX.I	647	62.76665	56.06304
	Darmor_AUP_FOSW_4_D09BTACXX.II	345	42.96125	37.04527
	Darmor_AUP_HOSW_5_D09BTACXX.I	239	33.28575	27.43133
	Darmor_AUP_IAOSW_1_1_C0UW4A(	1288	41.8941	39.23428
	Darmor_AUP_KAOSW_2_1_C0UW4A	1364	44.45589	41.3923
	Darmor_AUP_LAOSW_3_1_C0UW4A(	1384	45.6642	42.27843
	Darmor_AUP_MAOSW_4_1_C0UW4A	1485	49.03291	45.77701
	Darmor_AUP_NAOSW_5_1_C0UW4A	1399	44.06983	41.14981
	Darmor_AUP_PAOSW_6_1_C0UW4A(	1431	49.77893	46.18059
	Ningyou_ERR037335	59	24.90686	18.46106
	TN04_ERR037297	0	1.44E-10	0

	TN05_ERR037298	0	4.47E-10	0
	TN08_ERR037301	0	8.43E-09	0
	TN103_ERR037317	70	26.98391	25.89149
	TN108_ERR037318	80	24.44135	22.17776
	TN109_ERR037319	75	24.07092	18.20331
	TN10_ERR037294	0	1.01E-09	0
	TN121_ERR037320	83	23.29914	23.29914
	TN124_ERR037321	0	4.16E-18	0
	TN126_ERR037322	0	1.22E-18	0
	TN128_ERR037323	94	26.12694	26.12662
	TN129_ERR037324	0	2.04E-15	0
	TN12_ERR037302	0	1.28E-08	0
	TN138_ERR037325	72	23.84992	23.61118
	TN149_ERR037326	0	1.20E-06	0
	TN161_ERR037327	0	8.09E-27	0
	TN163_ERR037328	0	1.95E-13	0
	TN170_ERR037329	89	25.28504	25.28478
	TN171_ERR037332	0	9.15E-27	0
	TN172_ERR037333	0	5.87E-11	0
	TN176_ERR037330	0	3.88E-07	0
	TN177_ERR037331	104	22.01969	22.01967
	TN24_ERR037299	64	21.16683	21.10572
	TN28_ERR037295	110	27.17997	26.90687
	TN30_ERR037305	102	27.25249	20.57212
	TN31_ERR037303	0	3.07E-10	0
	TN38_ERR037304	2	0.6537127	0.2696128
	TN44_ERR037306	80	22.98374	19.28119
	TN48_ERR037307	101	23.46156	17.62508
	TN51_ERR037308	92	22.94108	18.04281
	TN57_ERR037309	117	27.77792	27.74619
	TN72_ERR037312	77	24.63552	24.53183
	TN76_ERR037311	115	29.59008	22.49515
	TN77_ERR037310	112	27.02519	25.61427

	TN85_ERR037315	91	25.37788	25.37751
	TN88_ERR037313	100	26.28983	22.02699
	TN90_ERR037316	1	0.2565246	0
	Tapidor_ERR037334	0	2.93E-13	0
BnaC03g33340.1D2	Darmor_AUP_AOSW_2_D09BTACXX.I	194	0.05804004	0.04682001
	Darmor_AUP_BOSW_3_D09BTACXX.I	235	0.1074157	0.08608987
	Darmor_AUP_DOSW_2_D09BTACXX.I	235	0.0790101	0.06770442
	Darmor_AUP_FOSW_4_D09BTACXX.I	64	0.0225171	0.01345029
	Darmor_AUP_HOSW_5_D09BTACXX.I	265	0.1028279	0.08761186
	Darmor_AUP_IAOSW_1_1_C0UW4A	680	0.1325794	0.1288198
	Darmor_AUP_KAOSW_2_1_C0UW4A	508	0.1029324	0.09599207
	Darmor_AUP_LAOSW_3_1_C0UW4A	222	0.04277595	0.04234677
	Darmor_AUP_MAOSW_4_1_C0UW4	206	0.03897268	0.0367014
	Darmor_AUP_NAOSW_5_1_C0UW4A	256	0.05008951	0.0456241
	Darmor_AUP_PAOSW_6_1_C0UW4A	640	0.1254564	0.1227954
	Ningyou_ERR037335	13	0.01469271	0.001896067
	TN04_ERR037297	13	0.0204273	0.01279458
	TN05_ERR037298	32	0.0308669	0.02078806
	TN08_ERR037301	30	0.02210064	0.01403415
	TN103_ERR037317	32	0.03204494	0.01598566
	TN108_ERR037318	18	0.01373999	0.007327073
	TN109_ERR037319	43	0.02615219	0.0222974
	TN10_ERR037294	26	0.02150821	0.005228236
	TN121_ERR037320	43	0.02556723	0.01501537
	TN124_ERR037321	31	0.03107457	0.01513304
	TN126_ERR037322	39	0.02061338	0.01393896
	TN128_ERR037323	23	0.02285334	0.006710188
	TN129_ERR037324	48	0.02296885	0.01855979
	TN12_ERR037302	50	0.05856927	0.04374173
	TN138_ERR037325	41	0.04779941	0.0431515
	TN149_ERR037326	27	0.0270643	0.02143813
	TN161_ERR037327	34	0.03042246	0.0184204
	TN163_ERR037328	27	0.02550583	0.007398542

	TN170_ERR037329	24	0.02658504	0.01384787
	TN171_ERR037332	25	0.01367617	0.00855703
	TN172_ERR037333	26	0.01352469	0.0110158
	TN176_ERR037330	46	0.04555307	0.03627248
	TN177_ERR037331	33	0.02552744	0.01600851
	TN24_ERR037299	22	0.01977323	0.00770046
	TN28_ERR037295	24	0.02269514	0.01455479
	TN30_ERR037305	45	0.02888644	0.01616611
	TN31_ERR037303	30	0.01962582	0.01199492
	TN38_ERR037304	32	0.03074774	0.018793
	TN44_ERR037306	25	0.02820312	0.01653149
	TN48_ERR037307	33	0.03835998	0.02033025
	TN51_ERR037308	13	0.01069046	0.002499771
	TN57_ERR037309	34	0.03662698	0.02582534
	TN72_ERR037312	30	0.0202571	0.01571573
	TN76_ERR037311	35	0.04918475	0.03624936
	TN77_ERR037310	18	0.02031436	0.006334208
	TN85_ERR037315	28	0.01951379	0.009987362
	TN88_ERR037313	38	0.02370056	0.01416884
	TN90_ERR037316	27	0.01865285	0.007692881
	Tapidor_ERR037334	21	0.02169435	0.01423283
BnaC04g14480.1D2	Darmor_AUP_AOSW_2_D09BTACXX.I	5	0.2571946	0.2499668
	Darmor_AUP_BOSW_3_D09BTACXX.I	7	0.308562	0.2270413
	Darmor_AUP_DOSW_2_D09BTACXX.I	22	1.535696	0.967652
	Darmor_AUP_FOSW_4_D09BTACXX.II	38	3.101234	1.851394
	Darmor_AUP_HOSW_5_D09BTACXX.I	76	3.630793	2.822493
	Darmor_AUP_IAOSW_1_1_C0UW4AC	6	0.09024911	0.08690048
	Darmor_AUP_KAOSW_2_1_C0UW4A	4	0.05984663	0.05974248
	Darmor_AUP_LAOSW_3_1_C0UW4A(	47	0.6979295	0.6364213
	Darmor_AUP_MAOSW_4_1_C0UW4A	52	0.7621571	0.7289217
	Darmor_AUP_NAOSW_5_1_C0UW4A	51	0.7556292	0.4929869
	Darmor_AUP_PAOSW_6_1_C0UW4A(	5	0.08905013	0.08843
	Ningyou_ERR037335	0	2.43E-12	0

	TN04_ERR037297	0	2.54E-23	0
	TN05_ERR037298	0	4.78E-24	0
	TN08_ERR037301	0	1.31E-26	0
	TN103_ERR037317	0	2.00E-20	0
	TN108_ERR037318	0	1.93E-23	0
	TN109_ERR037319	0	4.74E-26	0
	TN10_ERR037294	0	2.99E-25	0
	TN121_ERR037320	0	2.17E-25	0
	TN124_ERR037321	0	2.17E-20	0
	TN126_ERR037322	1	0.04554564	0.04545993
	TN128_ERR037323	0	1.36E-21	0
	TN129_ERR037324	0	2.32E-17	0
	TN12_ERR037302	0	1.15E-24	0
	TN138_ERR037325	0	2.00E-23	0
	TN149_ERR037326	0	1.05E-21	0
	TN161_ERR037327	0	5.20E-25	0
	TN163_ERR037328	0	4.79E-25	0
	TN170_ERR037329	0	1.09E-25	0
	TN171_ERR037332	0	7.28E-26	0
	TN172_ERR037333	0	2.60E-20	0
	TN176_ERR037330	0	6.72E-24	0
	TN177_ERR037331	0	8.41E-23	0
	TN24_ERR037299	0	1.08E-23	0
	TN28_ERR037295	0	1.24E-20	0
	TN30_ERR037305	0	3.39E-23	0
	TN31_ERR037303	0	4.44E-25	0
	TN38_ERR037304	0	6.74E-26	0
	TN44_ERR037306	0	3.64E-24	0
	TN48_ERR037307	0	2.51E-25	0
	TN51_ERR037308	0	1.98E-21	0
	TN57_ERR037309	0	6.70E-21	0
	TN72_ERR037312	0	8.73E-24	0
	TN76_ERR037311	0	1.97E-22	0

	TN77_ERR037310	0	3.87E-24	0
	TN85_ERR037315	0	1.58E-24	0
	TN88_ERR037313	0	1.28E-16	0
	TN90_ERR037316	0	1.58E-24	0
	Tapidor_ERR037334	0	4.06E-19	0
BnaC02g08000.1D2	Darmor_AUP_AOSW_2_D09BTACXX.I	249	6.537989	5.844607
	Darmor_AUP_BOSW_3_D09BTACXX.I	362	9.42626	8.506588
	Darmor_AUP_DOSW_2_D09BTACXX.I	674	13.43929	12.5323
	Darmor_AUP_FOSW_4_D09BTACXX.II	1411	40.97367	39.45954
	Darmor_AUP_HOSW_5_D09BTACXX.I	461	13.8649	12.68963
	Darmor_AUP_IAOSW_1_1_C0UW4AC	797	7.546845	7.436459
	Darmor_AUP_KAOSW_2_1_C0UW4A	819	7.440279	7.193321
	Darmor_AUP_LAOSW_3_1_C0UW4AC	4552	41.39	40.71676
	Darmor_AUP_MAOSW_4_1_C0UW4A	4852	42.59436	42.01363
	Darmor_AUP_NAOSW_5_1_C0UW4A	4151	36.91706	36.45848
	Darmor_AUP_PAOSW_6_1_C0UW4AC	865	8.02104	7.694958
	Ningyou_ERR037335	67	3.141641	2.826339
	TN04_ERR037297	72	2.083286	1.630669
	TN05_ERR037298	96	2.843315	2.635375
	TN08_ERR037301	54	1.649679	1.300302
	TN103_ERR037317	78	2.911947	2.291459
	TN108_ERR037318	71	2.751777	1.994908
	TN109_ERR037319	53	1.634287	1.029777
	TN10_ERR037294	85	2.574366	1.923957
	TN121_ERR037320	66	1.929897	1.520202
	TN124_ERR037321	66	1.901429	1.670582
	TN126_ERR037322	74	1.894387	1.378915
	TN128_ERR037323	67	1.811636	1.566698
	TN129_ERR037324	55	1.532092	1.243049
	TN12_ERR037302	60	1.852053	1.511299
	TN138_ERR037325	92	3.824997	3.329741
	TN149_ERR037326	118	4.058574	3.247028
	TN161_ERR037327	54	3.174211	2.527814

	TN163_ERR037328	102	3.015798	2.767912
	TN170_ERR037329	63	1.717943	1.300485
	TN171_ERR037332	51	1.357935	1.135791
	TN172_ERR037333	63	2.385653	2.182012
	TN176_ERR037330	56	1.374926	0.9721946
	TN177_ERR037331	48	1.633856	1.432835
	TN24_ERR037299	94	3.423182	3.197529
	TN28_ERR037295	61	1.896727	1.566263
	TN30_ERR037305	76	2.258782	1.933112
	TN31_ERR037303	66	2.454901	1.870184
	TN38_ERR037304	83	2.519871	2.376583
	TN44_ERR037306	60	2.746401	2.478193
	TN48_ERR037307	49	1.855774	1.733731
	TN51_ERR037308	53	2.090331	1.788808
	TN57_ERR037309	75	1.948491	1.682748
	TN72_ERR037312	63	2.046936	1.720299
	TN76_ERR037311	82	2.8194	2.169395
	TN77_ERR037310	79	2.114076	1.880826
	TN85_ERR037315	58	1.787836	1.393168
	TN88_ERR037313	75	2.736764	2.249802
	TN90_ERR037316	62	1.755621	1.540172
	Tapidor_ERR037334	62	2.79378	1.731923
BnaA01g22340.1D2	Darmor_AUP_AOSW_2_D09BTACXX.I	1	0.05871608	0.05871607
	Darmor_AUP_BOSW_3_D09BTACXX.I	2	0.1618442	0.1538344
	Darmor_AUP_DOSW_2_D09BTACXX.I	8	0.5577702	0.5568021
	Darmor_AUP_FOSW_4_D09BTACXX.I	71	5.675686	3.469503
	Darmor_AUP_HOSW_5_D09BTACXX.I	28	2.260398	2.253158
	Darmor_AUP_IAOSW_1_1_C0UW4A	14	0.4128382	0.4120055
	Darmor_AUP_KAOSW_2_1_C0UW4A	8	0.224701	0.2151452
	Darmor_AUP_LAOSW_3_1_C0UW4A	171	4.273236	4.270371
	Darmor_AUP_MAOSW_4_1_C0UW4	162	3.752326	3.74929
	Darmor_AUP_NAOSW_5_1_C0UW4A	232	5.493415	5.493415
	Darmor_AUP_PAOSW_6_1_C0UW4A	10	0.2407932	0.2407932

	Ningyou_ERR037335	9	0.8086392	0.567286
	TN04_ERR037297	0	2.57E-22	0
	TN05_ERR037298	0	4.44E-20	0
	TN08_ERR037301	5	0.4333055	0.2077385
	TN103_ERR037317	6	0.8345375	0.2738877
	TN108_ERR037318	0	0	0
	TN109_ERR037319	12	0.9897238	0.24624
	TN10_ERR037294	5	0.7331901	0
	TN121_ERR037320	18	2.073344	0.2737136
	TN124_ERR037321	7	0.4879045	0.4619938
	TN126_ERR037322	7	0.5888597	0.5853092
	TN128_ERR037323	0	0.7756904	0.7756904
	TN129_ERR037324	9	0.6068047	0.6006112
	TN12_ERR037302	0	1.11E-28	0
	TN138_ERR037325	0	1.93E-28	0
	TN149_ERR037326	5	0.4138616	0.4054508
	TN161_ERR037327	0	7.21E-30	0
	TN163_ERR037328	0	5.99E-21	0
	TN170_ERR037329	11	0.7537834	0.3608189
	TN171_ERR037332	0	1.09E-26	0
	TN172_ERR037333	0	4.39E-31	0
	TN176_ERR037330	0	1.13E-18	0
	TN177_ERR037331	5	0.3734264	0.3591145
	TN24_ERR037299	6	0.5728565	0.5726827
	TN28_ERR037295	0	0.8243623	0.8243623
	TN30_ERR037305	15	1.262548	1.222729
	TN31_ERR037303	7	0.5621219	0.5221871
	TN38_ERR037304	13	1.971809	0.1899753
	TN44_ERR037306	0	6.34E-28	0
	TN48_ERR037307	0	2.60E-24	0
	TN51_ERR037308	0	7.73E-30	0
	TN57_ERR037309	6	0.4332255	0.0337574
	TN72_ERR037312	0	6.64E-28	0

	TN76_ERR037311	10	1.232208	1.169595
	TN77_ERR037310	0	2.56E-18	0
	TN85_ERR037315	10	0.8432185	0.8427985
	TN88_ERR037313	0	6.33E-27	0
	TN90_ERR037316	0	4.77E-20	0
	Tapidor_ERR037334	0	1.68E-16	0
BnaC02g07860.1D2	Darmor_AUP_AOSW_2_D09BTACXX.I	74	1.554962	1.524636
	Darmor_AUP_BOSW_3_D09BTACXX.I	55	1.707509	1.634093
	Darmor_AUP_DOSW_2_D09BTACXX.I	97	2.100408	1.857183
	Darmor_AUP_FOSW_4_D09BTACXX.II	90	2.329984	2.155247
	Darmor_AUP_HOSW_5_D09BTACXX.I	83	2.406637	2.275906
	Darmor_AUP_IAOSW_1_1_C0UW4AC	125	1.37454	1.343011
	Darmor_AUP_KAOSW_2_1_C0UW4A	103	1.132242	1.132242
	Darmor_AUP_LAOSW_3_1_C0UW4AC	116	1.200274	1.200274
	Darmor_AUP_MAOSW_4_1_C0UW4A	141	1.468079	1.468079
	Darmor_AUP_NAOSW_5_1_C0UW4A	86	0.9019415	0.9019415
	Darmor_AUP_PAOSW_6_1_C0UW4AC	123	1.301899	1.301899
	Ningyou_ERR037335	0	3.37E-19	0
	TN04_ERR037297	0	6.89E-22	0
	TN05_ERR037298	0	7.54E-27	0
	TN08_ERR037301	0	6.17E-29	0
	TN103_ERR037317	0	1.72E-22	0
	TN108_ERR037318	0	5.57E-27	0
	TN109_ERR037319	0	9.21E-29	0
	TN10_ERR037294	0	1.18E-20	0
	TN121_ERR037320	0	4.99E-32	0
	TN124_ERR037321	0	5.70E-21	0
	TN126_ERR037322	0	8.26E-27	0
	TN128_ERR037323	0	3.82E-27	0
	TN129_ERR037324	0	1.73E-29	0
	TN12_ERR037302	0	1.43E-17	0
	TN138_ERR037325	0	4.49E-28	0
	TN149_ERR037326	0	7.39E-25	0

	TN161_ERR037327	0	3.67E-27	0
	TN163_ERR037328	0	1.11E-25	0
	TN170_ERR037329	0	1.36E-29	0
	TN171_ERR037332	0	1.36E-31	0
	TN172_ERR037333	0	1.07E-26	0
	TN176_ERR037330	0	1.05E-30	0
	TN177_ERR037331	0	8.67E-29	0
	TN24_ERR037299	0	3.84E-27	0
	TN28_ERR037295	0	2.26E-29	0
	TN30_ERR037305	0	4.42E-33	0
	TN31_ERR037303	0	5.57E-28	0
	TN38_ERR037304	0	1.13E-26	0
	TN44_ERR037306	0	3.66E-27	0
	TN48_ERR037307	0	0	0
	TN51_ERR037308	0	7.52E-26	0
	TN57_ERR037309	0	2.47E-30	0
	TN72_ERR037312	0	1.16E-16	0
	TN76_ERR037311	0	3.94E-27	0
	TN77_ERR037310	0	1.21E-25	0
	TN85_ERR037315	0	2.29E-19	0
	TN88_ERR037313	0	1.53E-28	0
	TN90_ERR037316	0	1.03E-15	0
	Tapidor_ERR037334	1	0.2252606	0.2252606
BnaC02g05610.1D2	Darmor_AUP_AOSW_2_D09BTACXX.I	479	58.15144	52.03464
	Darmor_AUP_BOSW_3_D09BTACXX.I	172	25.51143	21.05608
	Darmor_AUP_DOSW_2_D09BTACXX.I	339	39.68554	34.507
	Darmor_AUP_FOSW_4_D09BTACXX.II	338	49.89699	42.2211
	Darmor_AUP_HOSW_5_D09BTACXX.I	248	40.5987	34.95497
	Darmor_AUP_IAOSW_1_1_C0UW4AC	770	29.09844	26.65526
	Darmor_AUP_KAOSW_2_1_C0UW4A	936	38.36974	35.42806
	Darmor_AUP_LAOSW_3_1_C0UW4AC	694	23.63687	21.32032
	Darmor_AUP_MAOSW_4_1_C0UW4A	953	33.74524	31.17805
	Darmor_AUP_NAOSW_5_1_C0UW4A	482	16.02605	14.20861

	Darmor_AUP_PAOSW_6_1_C0UW4A(	731	28.08053	25.78969
	Ningyou_ERR037335	22	9.647213	5.102836
	TN04_ERR037297	14	4.06997	1.633325
	TN05_ERR037298	25	4.0126	2.091744
	TN08_ERR037301	5	0.8707576	0
	TN103_ERR037317	21	4.492329	1.958238
	TN108_ERR037318	16	5.776228	3.289946
	TN109_ERR037319	7	4.644198	1.211714
	TN10_ERR037294	27	10.3723	6.083649
	TN121_ERR037320	13	2.500262	0.9990895
	TN124_ERR037321	4	1.052223	0
	TN126_ERR037322	28	7.462723	4.407219
	TN128_ERR037323	0	3.07E-07	0
	TN129_ERR037324	23	9.597841	4.741196
	TN12_ERR037302	9	1.872715	0.6862768
	TN138_ERR037325	19	8.899919	3.465187
	TN149_ERR037326	6	3.639455	0
	TN161_ERR037327	7	2.025434	0.08253549
	TN163_ERR037328	37	6.58682	3.704832
	TN170_ERR037329	45	10.21211	5.61198
	TN171_ERR037332	0	7.12E-21	0
	TN172_ERR037333	50	19.39259	13.69413
	TN176_ERR037330	15	4.288025	1.045306
	TN177_ERR037331	3	0.9065643	0
	TN24_ERR037299	0	2.77E-18	0
	TN28_ERR037295	23	5.107838	1.950228
	TN30_ERR037305	12	3.521442	1.606803
	TN31_ERR037303	6	2.49585	0.5003817
	TN38_ERR037304	18	2.684363	1.241217
	TN44_ERR037306	15	2.604026	0.8794548
	TN48_ERR037307	8	1.546145	0.1183447
	TN51_ERR037308	26	13.29719	8.524607
	TN57_ERR037309	6	0.7587903	0

	TN72_ERR037312	4	2.153364	0
	TN76_ERR037311	9	1.707694	0.2166823
	TN77_ERR037310	0	3.14E-17	0
	TN85_ERR037315	8	0.8933477	0.3966989
	TN88_ERR037313	0	1.21E-22	0
	TN90_ERR037316	3	1.855765	0.1293415
	Tapidor_ERR037334	0	2.80E-15	0
BnaC03g01420.1D2	Darmor_AUP_AOSW_2_D09BTACXX.I	976	375.7337	364.6867
	Darmor_AUP_BOSW_3_D09BTACXX.I	808	428.7438	428.2135
	Darmor_AUP_DOSW_2_D09BTACXX.I	1491	546.5017	544.8565
	Darmor_AUP_FOSW_4_D09BTACXX.I	142	64.18667	56.01926
	Darmor_AUP_HOSW_5_D09BTACXX.I	331	175.313	175.2959
	Darmor_AUP_IAOSW_1_1_C0UW4A(	1099	188.1577	188.132
	Darmor_AUP_KAOSW_2_1_C0UW4A	975	168.2049	168.2049
	Darmor_AUP_LAOSW_3_1_C0UW4A(	284	46.82536	46.82536
	Darmor_AUP_MAOSW_4_1_C0UW4A	291	48.75788	48.75788
	Darmor_AUP_NAOSW_5_1_C0UW4A	281	48.95338	48.46902
	Darmor_AUP_PAOSW_6_1_C0UW4A(	1159	193.4483	191.6065
	Ningyou_ERR037335	29	32.25464	18.73363
	TN04_ERR037297	0	9.39E-34	0
	TN05_ERR037298	0	6.65E-25	0
	TN08_ERR037301	0	9.08E-33	0
	TN103_ERR037317	0	3.09E-30	0
	TN108_ERR037318	0	5.87E-30	0
	TN109_ERR037319	0	4.41E-31	0
	TN10_ERR037294	0	4.63E-25	0
	TN121_ERR037320	0	4.38E-31	0
	TN124_ERR037321	0	6.29E-27	0
	TN126_ERR037322	0	1.40E-31	0
	TN128_ERR037323	0	6.36E-32	0
	TN129_ERR037324	0	2.14E-29	0
	TN12_ERR037302	0	2.99E-31	0
	TN138_ERR037325	0	4.63E-31	0

	TN149_ERR037326	0	2.36E-30	0
	TN161_ERR037327	72	52.23678	49.17781
	TN163_ERR037328	51	44.33629	34.98809
	TN170_ERR037329	0	1.25E-21	0
	TN171_ERR037332	0	2.85E-31	0
	TN172_ERR037333	0	5.23E-32	0
	TN176_ERR037330	0	1.82E-32	0
	TN177_ERR037331	0	3.96E-31	0
	TN24_ERR037299	0	1.27E-29	0
	TN28_ERR037295	0	6.99E-31	0
	TN30_ERR037305	0	9.08E-22	0
	TN31_ERR037303	46	41.49689	26.29227
	TN38_ERR037304	4	7.257668	7.255622
	TN44_ERR037306	0	1.24E-29	0
	TN48_ERR037307	0	1.76E-26	0
	TN51_ERR037308	34	28.12235	27.48688
	TN57_ERR037309	74	49.82061	38.75083
	TN72_ERR037312	0	1.69E-30	0
	TN76_ERR037311	0	4.29E-26	0
	TN77_ERR037310	83	68.18577	56.32613
	TN85_ERR037315	0	3.27E-26	0
	TN88_ERR037313	0	1.85E-31	0
	TN90_ERR037316	0	4.76E-31	0
	Tapidor_ERR037334	0	4.05E-31	0
BnaC03g01240.1D2	Darmor_AUP_AOSW_2_D09BTACXX.I	329	18.93125	16.82332
	Darmor_AUP_BOSW_3_D09BTACXX.I	343	22.66326	22.10787
	Darmor_AUP_DOSW_2_D09BTACXX.I	618	32.80293	30.7391
	Darmor_AUP_FOSW_4_D09BTACXX.II	722	42.98818	40.32265
	Darmor_AUP_HOSW_5_D09BTACXX.I	435	36.30569	33.13361
	Darmor_AUP_IAOSW_1_1_C0UW4AC	668	13.16641	12.57208
	Darmor_AUP_KAOSW_2_1_C0UW4A	812	16.07471	15.84718
	Darmor_AUP_LAOSW_3_1_C0UW4AC	1970	39.40222	39.06919
	Darmor_AUP_MAOSW_4_1_C0UW4A	2100	41.20447	40.92874

	Darmor_AUP_NAOSW_5_1_C0UW4A	2042	40.06602	39.54423
	Darmor_AUP_PAOSW_6_1_C0UW4A(	785	15.77892	15.77789
	Ningyou_ERR037335	254	30.77536	25.76413
	TN04_ERR037297	21	2.122667	0.7694138
	TN05_ERR037298	32	4.365476	3.156863
	TN08_ERR037301	32	2.33663	1.535424
	TN103_ERR037317	16	1.409474	0.8931431
	TN108_ERR037318	19	3.618741	3.068927
	TN109_ERR037319	0	5.49E-21	0
	TN10_ERR037294	0	0.00025937	0
	TN121_ERR037320	0	5.20E-20	0
	TN124_ERR037321	11	1.062276	0
	TN126_ERR037322	27	1.652607	1.383846
	TN128_ERR037323	21	1.238472	0.9708493
	TN129_ERR037324	0	3.11E-22	0
	TN12_ERR037302	26	1.811302	1.16747
	TN138_ERR037325	19	1.449932	1.202472
	TN149_ERR037326	30	2.458189	1.675193
	TN161_ERR037327	229	17.45239	15.42676
	TN163_ERR037328	334	23.36589	22.3356
	TN170_ERR037329	0	4.60E-26	0
	TN171_ERR037332	0	1.48E-26	0
	TN172_ERR037333	26	1.593742	0.7443928
	TN176_ERR037330	0	1.57E-26	0
	TN177_ERR037331	0	2.06E-24	0
	TN24_ERR037299	0	7.75E-24	0
	TN28_ERR037295	18	1.888799	0.3735902
	TN30_ERR037305	24	2.798755	2.036865
	TN31_ERR037303	340	27.55879	24.23059
	TN38_ERR037304	4	0.7427778	0
	TN44_ERR037306	14	3.384373	2.380645
	TN48_ERR037307	27	3.73014	2.016194
	TN51_ERR037308	254	24.47102	20.83488

	TN57_ERR037309	374	25.58465	21.91905
	TN72_ERR037312	2	0.1487114	0.1419322
	TN76_ERR037311	19	2.132124	1.735787
	TN77_ERR037310	386	30.31972	26.95275
	TN85_ERR037315	0	1.44E-19	0
	TN88_ERR037313	0	1.24E-26	0
	TN90_ERR037316	0	1.79E-27	0
	Tapidor_ERR037334	0	3.21E-27	0
BnaC03g02570.1D2	Darmor_AUP_AOSW_2_D09BTACXX.I	0	0	0
	Darmor_AUP_BOSW_3_D09BTACXX.I	1	0.1772325	0.1772325
	Darmor_AUP_DOSW_2_D09BTACXX.I	0	0	0
	Darmor_AUP_FOSW_4_D09BTACXX.II	0	0	0
	Darmor_AUP_HOSW_5_D09BTACXX.I	0	0	0
	Darmor_AUP_IAOSW_1_1_C0UW4A(	8	0.4315193	0.4315193
	Darmor_AUP_KAOSW_2_1_C0UW4A	2	0.1116509	0.1116509
	Darmor_AUP_LAOSW_3_1_C0UW4A(	0	0	0
	Darmor_AUP_MAOSW_4_1_C0UW4A	0	0	0
	Darmor_AUP_NAOSW_5_1_C0UW4A	0	0	0
	Darmor_AUP_PAOSW_6_1_C0UW4A(	7	0.3884271	0.3884271
	Ningyou_ERR037335	0	0	0
	TN04_ERR037297	0	0	0
	TN05_ERR037298	0	0	0
	TN08_ERR037301	0	0	0
	TN103_ERR037317	0	0	0
	TN108_ERR037318	0	0	0
	TN109_ERR037319	0	0	0
	TN10_ERR037294	0	0	0
	TN121_ERR037320	0	0	0
	TN124_ERR037321	0	0	0
	TN126_ERR037322	0	0	0
	TN128_ERR037323	0	0	0
	TN129_ERR037324	0	0	0
	TN12_ERR037302	0	0	0

	TN138_ERR037325	0	0	0
	TN149_ERR037326	0	0	0
	TN161_ERR037327	0	0	0
	TN163_ERR037328	0	0	0
	TN170_ERR037329	0	0	0
	TN171_ERR037332	0	0	0
	TN172_ERR037333	0	0	0
	TN176_ERR037330	0	0	0
	TN177_ERR037331	0	0	0
	TN24_ERR037299	0	0	0
	TN28_ERR037295	0	0	0
	TN30_ERR037305	0	0	0
	TN31_ERR037303	0	0	0
	TN38_ERR037304	0	0	0
	TN44_ERR037306	0	0	0
	TN48_ERR037307	0	0	0
	TN51_ERR037308	0	0	0
	TN57_ERR037309	0	0	0
	TN72_ERR037312	0	0	0
	TN76_ERR037311	0	0	0
	TN77_ERR037310	0	0	0
	TN85_ERR037315	0	0	0
	TN88_ERR037313	0	0	0
	TN90_ERR037316	0	0	0
	Tapidor_ERR037334	0	0	0
BnaA03g30720.1D2	Darmor_AUP_AOSW_2_D09BTACXX.I	2	0.05665441	0.05665441
	Darmor_AUP_BOSW_3_D09BTACXX.I	16	0.6148227	0.6148227
	Darmor_AUP_DOSW_2_D09BTACXX.I	1	0.02704896	0.02704896
	Darmor_AUP_FOSW_4_D09BTACXX.II	1	0.03467474	0.03467474
	Darmor_AUP_HOSW_5_D09BTACXX.I	6	0.2408148	0.2408148
	Darmor_AUP_IAOSW_1_1_C0UW4AC	18	0.2316603	0.2316603
	Darmor_AUP_KAOSW_2_1_C0UW4A	33	0.4253284	0.4253284
	Darmor_AUP_LAOSW_3_1_C0UW4AC	1	0.01233659	0.01233659

	Darmor_AUP_MAOSW_4_1_C0UW4A	2	0.024664	0.024664
	Darmor_AUP_NAOSW_5_1_C0UW4A	2	0.02510267	0.02510267
	Darmor_AUP_PAOSW_6_1_C0UW4A	29	0.364806	0.364806
	Ningyou_ERR037335	0	0	0
	TN04_ERR037297	0	0	0
	TN05_ERR037298	0	0	0
	TN08_ERR037301	0	0	0
	TN103_ERR037317	0	0	0
	TN108_ERR037318	0	0	0
	TN109_ERR037319	0	0	0
	TN10_ERR037294	0	0	0
	TN121_ERR037320	0	0	0
	TN124_ERR037321	0	0	0
	TN126_ERR037322	0	0	0
	TN128_ERR037323	0	0	0
	TN129_ERR037324	0	0	0
	TN12_ERR037302	0	0	0
	TN138_ERR037325	0	0	0
	TN149_ERR037326	0	0	0
	TN161_ERR037327	0	0	0
	TN163_ERR037328	0	0	0
	TN170_ERR037329	0	0	0
	TN171_ERR037332	0	0	0
	TN172_ERR037333	0	0	0
	TN176_ERR037330	0	0	0
	TN177_ERR037331	0	0	0
	TN24_ERR037299	0	0	0
	TN28_ERR037295	0	0	0
	TN30_ERR037305	0	0	0
	TN31_ERR037303	0	0	0
	TN38_ERR037304	0	0	0
	TN44_ERR037306	0	0	0
	TN48_ERR037307	0	0	0

	TN51_ERR037308	0	0	0
	TN57_ERR037309	0	0	0
	TN72_ERR037312	0	0	0
	TN76_ERR037311	0	0	0
	TN77_ERR037310	0	0	0
	TN85_ERR037315	0	0	0
	TN88_ERR037313	0	0	0
	TN90_ERR037316	0	0	0
	Tapidor_ERR037334	0	0	0
BnaU01g10730.1D2	Darmor_AUP_AOSW_2_D09BTACXX.I	22	1.814059	0.3747785
	Darmor_AUP_BOSW_3_D09BTACXX.I	13	1.340183	0.6637104
	Darmor_AUP_DOSW_2_D09BTACXX.I	49	6.04932	5.284559
	Darmor_AUP_FOSW_4_D09BTACXX.II	83	11.18834	8.819192
	Darmor_AUP_HOSW_5_D09BTACXX.I	36	5.114523	3.261963
	Darmor_AUP_IAOSW_1_1_C0UW4AC	17	1.109717	0.2613602
	Darmor_AUP_KAOSW_2_1_C0UW4A	24	1.125146	0.8130621
	Darmor_AUP_LAOSW_3_1_C0UW4AC	139	9.90934	7.881545
	Darmor_AUP_MAOSW_4_1_C0UW4A	143	8.620421	6.666839
	Darmor_AUP_NAOSW_5_1_C0UW4A	131	6.538969	4.860929
	Darmor_AUP_PAOSW_6_1_C0UW4AC	27	0.9459374	0.6933315
	Ningyou_ERR037335	15	4.621006	2.61803
	TN04_ERR037297	12	1.43347	0
	TN05_ERR037298	3	0.3346485	0.3232996
	TN08_ERR037301	1	0.3006631	0
	TN103_ERR037317	8	3.115021	0
	TN108_ERR037318	9	1.286919	0
	TN109_ERR037319	0	1.75E-26	0
	TN10_ERR037294	1	0.4549175	0.4062626
	TN121_ERR037320	12	2.144018	0.5539923
	TN124_ERR037321	3	0.306836	0.2549156
	TN126_ERR037322	0	1.73E-19	0
	TN128_ERR037323	0	2.35E-18	0
	TN129_ERR037324	0	1.65E-32	0

	TN12_ERR037302	0	7.26E-27	0
	TN138_ERR037325	0	1.94E-11	0
	TN149_ERR037326	3	1.242807	0
	TN161_ERR037327	1	0.1481251	0.1481251
	TN163_ERR037328	5	0.6296827	0.6281801
	TN170_ERR037329	14	2.744859	1.502553
	TN171_ERR037332	1	0.1034265	0.1034253
	TN172_ERR037333	27	5.10464	1.859951
	TN176_ERR037330	3	1.806306	1.607926
	TN177_ERR037331	7	1.775017	0
	TN24_ERR037299	0	1.57E-19	0
	TN28_ERR037295	1	1.047783	1.047088
	TN30_ERR037305	0	7.61E-30	0
	TN31_ERR037303	2	0.237919	0.120521
	TN38_ERR037304	3	0.3402365	0
	TN44_ERR037306	3	1.343278	1.32939
	TN48_ERR037307	3	0.3623984	0.353011
	TN51_ERR037308	11	1.7224	0.5872979
	TN57_ERR037309	2	0.3116617	0.3101395
	TN72_ERR037312	0	5.82E-15	0
	TN76_ERR037311	0	7.58E-05	0
	TN77_ERR037310	0	0.1422029	0
	TN85_ERR037315	10	2.413457	0
	TN88_ERR037313	1	0.1088254	0.1087929
	TN90_ERR037316	6	0.6943127	0.1752453
	Tapidor_ERR037334	3	0.3884005	0.3871062
BnaC05g20750.1D2	Darmor_AUP_AOSW_2_D09BTACXX.I	0	0	0
	Darmor_AUP_BOSW_3_D09BTACXX.I	0	0	0
	Darmor_AUP_DOSW_2_D09BTACXX.I	0	0	0
	Darmor_AUP_FOSW_4_D09BTACXX.I	0	0	0
	Darmor_AUP_HOSW_5_D09BTACXX.I	0	0	0
	Darmor_AUP_IAOSW_1_1_C0UW4AC	0	0	0
	Darmor_AUP_KAOSW_2_1_C0UW4A	0	0	0

	Darmor_AUP_LAOSW_3_1_C0UW4A(	0	0	0
	Darmor_AUP_MAOSW_4_1_C0UW4A	0	0	0
	Darmor_AUP_NAOSW_5_1_C0UW4A	0	0	0
	Darmor_AUP_PAOSW_6_1_C0UW4A(	0	0	0
	Ningyou_ERR037335	0	0	0
	TN04_ERR037297	0	0	0
	TN05_ERR037298	0	0	0
	TN08_ERR037301	0	0	0
	TN103_ERR037317	0	0	0
	TN108_ERR037318	0	0	0
	TN109_ERR037319	0	0	0
	TN10_ERR037294	0	0	0
	TN121_ERR037320	0	0	0
	TN124_ERR037321	0	0	0
	TN126_ERR037322	0	0	0
	TN128_ERR037323	0	0	0
	TN129_ERR037324	0	0	0
	TN12_ERR037302	0	0	0
	TN138_ERR037325	0	0	0
	TN149_ERR037326	0	0	0
	TN161_ERR037327	0	0	0
	TN163_ERR037328	0	0	0
	TN170_ERR037329	0	0	0
	TN171_ERR037332	0	0	0
	TN172_ERR037333	0	0	0
	TN176_ERR037330	0	0	0
	TN177_ERR037331	0	0	0
	TN24_ERR037299	0	0	0
	TN28_ERR037295	0	0	0
	TN30_ERR037305	0	0	0
	TN31_ERR037303	0	0	0
	TN38_ERR037304	0	0	0
	TN44_ERR037306	0	0	0

	TN48_ERR037307	0	0	0
	TN51_ERR037308	0	0	0
	TN57_ERR037309	0	0	0
	TN72_ERR037312	0	0	0
	TN76_ERR037311	0	0	0
	TN77_ERR037310	0	0	0
	TN85_ERR037315	0	0	0
	TN88_ERR037313	0	0	0
	TN90_ERR037316	0	0	0
	Tapidor_ERR037334	0	0	0
BnaC06g03070.1D2	Darmor_AUP_AOSW_2_D09BTACXX.I	1	0.0574894	0.0574894
	Darmor_AUP_BOSW_3_D09BTACXX.I	3	0.2276234	0.2276234
	Darmor_AUP_DOSW_2_D09BTACXX.I	1	0.05143338	0.05143338
	Darmor_AUP_FOSW_4_D09BTACXX.II	0	0	0
	Darmor_AUP_HOSW_5_D09BTACXX.I	4	0.3135374	0.3135374
	Darmor_AUP_IAOSW_1_1_C0UW4AC	1	0.02205253	0.02205253
	Darmor_AUP_KAOSW_2_1_C0UW4A	2	0.04469839	0.04469839
	Darmor_AUP_LAOSW_3_1_C0UW4AC	0	0	0
	Darmor_AUP_MAOSW_4_1_C0UW4A	0	0	0
	Darmor_AUP_NAOSW_5_1_C0UW4A	0	0	0
	Darmor_AUP_PAOSW_6_1_C0UW4AC	5	0.1165545	0.1165545
	Ningyou_ERR037335	3	0.2803831	0.2803831
	TN04_ERR037297	0	0	0
	TN05_ERR037298	0	0	0
	TN08_ERR037301	0	0	0
	TN103_ERR037317	3	0.3211548	0.3211548
	TN108_ERR037318	0	0	0
	TN109_ERR037319	1	0.09148476	0.09148476
	TN10_ERR037294	5	0.3624672	0.3624672
	TN121_ERR037320	0	0	0
	TN124_ERR037321	4	0.3102323	0.3102323
	TN126_ERR037322	0	0	0
	TN128_ERR037323	6	0.4462721	0.4462721

	TN129_ERR037324	0	0	0
	TN12_ERR037302	4	0.3252679	0.3252679
	TN138_ERR037325	0	0	0
	TN149_ERR037326	0	0	0
	TN161_ERR037327	3	0.328158	0.328158
	TN163_ERR037328	3	0.276141	0.276141
	TN170_ERR037329	5	0.3921986	0.3921986
	TN171_ERR037332	5	0.3632324	0.3632324
	TN172_ERR037333	5	0.378917	0.378917
	TN176_ERR037330	7	0.5138642	0.5138642
	TN177_ERR037331	0	0	0
	TN24_ERR037299	4	0.4227376	0.4227376
	TN28_ERR037295	4	0.3017728	0.3017728
	TN30_ERR037305	3	0.2404244	0.2404244
	TN31_ERR037303	4	0.3382186	0.3382186
	TN38_ERR037304	8	0.6494722	0.6494722
	TN44_ERR037306	3	0.3337219	0.3337219
	TN48_ERR037307	0	0	0
	TN51_ERR037308	4	0.4035713	0.4035713
	TN57_ERR037309	5	0.3903311	0.3903311
	TN72_ERR037312	0	0	0
	TN76_ERR037311	5	0.4299084	0.4299084
	TN77_ERR037310	6	0.5133902	0.5133902
	TN85_ERR037315	2	0.196044	0.196044
	TN88_ERR037313	0	0	0
	TN90_ERR037316	0	0	0
	Tapidor_ERR037334	0	0	0
BnaC03g01310.1D2	Darmor_AUP_AOSW_2_D09BTACXX.I	150	8.789344	7.427899
	Darmor_AUP_BOSW_3_D09BTACXX.I	260	21.79167	19.67434
	Darmor_AUP_DOSW_2_D09BTACXX.I	282	12.7034	10.57285
	Darmor_AUP_FOSW_4_D09BTACXX.II	105	6.790157	4.945178
	Darmor_AUP_HOSW_5_D09BTACXX.I	560	36.86864	34.27222
	Darmor_AUP_IAOSW_1_1_C0UW4AC	658	13.11679	12.91711

	Darmor_AUP_KAOSW_2_1_C0UW4A	408	7.88225	7.661547
	Darmor_AUP_LAOSW_3_1_C0UW4A(	360	6.941118	6.938844
	Darmor_AUP_MAOSW_4_1_C0UW4/	355	6.814326	6.542657
	Darmor_AUP_NAOSW_5_1_C0UW4A	298	5.693649	5.443007
	Darmor_AUP_PAOSW_6_1_C0UW4A(	538	11.08162	10.28929
	Ningyou_ERR037335	13	1.338568	0.03906683
	TN04_ERR037297	1	0.06298227	0.06298192
	TN05_ERR037298	0	0.05897072	0.05897072
	TN08_ERR037301	0	9.33E-31	0
	TN103_ERR037317	0	1.48E-15	0
	TN108_ERR037318	0	7.01E-17	0
	TN109_ERR037319	0	0.1339321	0.133932
	TN10_ERR037294	0	9.52E-29	0
	TN121_ERR037320	0	6.20E-27	0
	TN124_ERR037321	0	0.112929	0.1129176
	TN126_ERR037322	0	0.05441094	0.05441094
	TN128_ERR037323	0	0.1632784	0.163254
	TN129_ERR037324	0	2.34E-16	0
	TN12_ERR037302	0	1.16E-07	1.16E-07
	TN138_ERR037325	0	6.87E-28	0
	TN149_ERR037326	0	2.00E-20	0
	TN161_ERR037327	13	2.646203	1.561538
	TN163_ERR037328	18	1.624379	0.4594963
	TN170_ERR037329	0	2.02E-20	0
	TN171_ERR037332	0	3.63E-27	0
	TN172_ERR037333	0	6.65E-19	0
	TN176_ERR037330	0	1.95E-29	0
	TN177_ERR037331	0	5.29E-20	0
	TN24_ERR037299	0	4.55E-20	0
	TN28_ERR037295	0	2.65E-24	0
	TN30_ERR037305	0	9.24E-17	0
	TN31_ERR037303	17	1.892609	0.2933503
	TN38_ERR037304	3	1.638264	0.0359094

	TN44_ERR037306	0	1.47E-07	0
	TN48_ERR037307	0	2.03E-23	0
	TN51_ERR037308	19	2.374478	1.076999
	TN57_ERR037309	24	2.211982	0.3943392
	TN72_ERR037312	0	3.43E-07	0
	TN76_ERR037311	1	0.07212034	0.07156005
	TN77_ERR037310	23	2.930965	1.735787
	TN85_ERR037315	0	0.1991505	0.1991505
	TN88_ERR037313	0	0.07458814	0
	TN90_ERR037316	0	1.95E-20	0
	Tapidor_ERR037334	0	0.000119674	0
BnaC06g28360.1D2	Darmor_AUP_AOSW_2_D09BTACXX.I	0	4.09E-30	0
	Darmor_AUP_BOSW_3_D09BTACXX.I	1	0.01588492	0.01588492
	Darmor_AUP_DOSW_2_D09BTACXX.I	0	6.24E-34	0
	Darmor_AUP_FOSW_4_D09BTACXX.II	0	3.84E-32	0
	Darmor_AUP_HOSW_5_D09BTACXX.I	9	0.1435557	0.1433747
	Darmor_AUP_IAOSW_1_1_C0UW4AC	1	0.00508663	0.00508663
	Darmor_AUP_KAOSW_2_1_C0UW4A	1	0.03083763	0.03083763
	Darmor_AUP_LAOSW_3_1_C0UW4A(	0	1.48E-42	0
	Darmor_AUP_MAOSW_4_1_C0UW4A	0	1.45E-42	0
	Darmor_AUP_NAOSW_5_1_C0UW4A	0	3.30E-39	0
	Darmor_AUP_PAOSW_6_1_C0UW4A(	1	0.01524736	0.01522267
	Ningyou_ERR037335	0	8.85E-28	0
	TN04_ERR037297	0	6.99E-24	0
	TN05_ERR037298	0	5.28E-31	0
	TN08_ERR037301	0	2.52E-21	0
	TN103_ERR037317	0	2.14E-16	0
	TN108_ERR037318	0	9.84E-32	0
	TN109_ERR037319	0	3.21E-21	0
	TN10_ERR037294	0	1.01E-29	0
	TN121_ERR037320	0	1.05E-21	0
	TN124_ERR037321	0	2.20E-30	0
	TN126_ERR037322	0	3.65E-24	0

	TN128_ERR037323	0	4.51E-28	0
	TN129_ERR037324	0	1.10E-24	0
	TN12_ERR037302	0	2.41E-24	0
	TN138_ERR037325	0	6.32E-24	0
	TN149_ERR037326	0	2.64E-29	0
	TN161_ERR037327	0	3.46E-29	0
	TN163_ERR037328	0	3.49E-29	0
	TN170_ERR037329	0	2.64E-29	0
	TN171_ERR037332	0	6.60E-22	0
	TN172_ERR037333	0	3.39E-21	0
	TN176_ERR037330	0	2.92E-24	0
	TN177_ERR037331	0	0.01648223	0.01648223
	TN24_ERR037299	0	9.11E-18	0
	TN28_ERR037295	0	6.14E-16	0
	TN30_ERR037305	0	6.06E-26	0
	TN31_ERR037303	0	1.44E-23	0
	TN38_ERR037304	0	2.67E-23	0
	TN44_ERR037306	0	4.42E-25	0
	TN48_ERR037307	0	8.19E-23	0
	TN51_ERR037308	0	2.59E-30	0
	TN57_ERR037309	0	4.52E-32	0
	TN72_ERR037312	0	1.85E-23	0
	TN76_ERR037311	0	5.31E-24	0
	TN77_ERR037310	0	1.94E-18	0
	TN85_ERR037315	0	3.61E-23	0
	TN88_ERR037313	0	1.08E-17	0
	TN90_ERR037316	0	8.86E-19	0
	Tapidor_ERR037334	0	6.43E-14	0
BnaC01g20150.1D2	Darmor_AUP_AOSW_2_D09BTACXX.I	0	5.86E-36	0
	Darmor_AUP_BOSW_3_D09BTACXX.I	0	1.17E-34	0
	Darmor_AUP_DOSW_2_D09BTACXX.I	0	0	0
	Darmor_AUP_FOSW_4_D09BTACXX.II	0	0	0
	Darmor_AUP_HOSW_5_D09BTACXX.I	0	0	0

	Darmor_AUP_IAOSW_1_1_C0UW4A(	0	0	0
	Darmor_AUP_KAOSW_2_1_C0UW4A	0	0	0
	Darmor_AUP_LAOSW_3_1_C0UW4A(	0	0	0
	Darmor_AUP_MAOSW_4_1_C0UW4A	0	0	0
	Darmor_AUP_NAOSW_5_1_C0UW4A	0	0	0
	Darmor_AUP_PAOSW_6_1_C0UW4A(	0	0	0
	Ningyou_ERR037335	0	7.03E-29	0
	TN04_ERR037297	0	1.94E-28	0
	TN05_ERR037298	0	4.87E-26	0
	TN08_ERR037301	0	5.97E-30	0
	TN103_ERR037317	0	2.09E-30	0
	TN108_ERR037318	0	2.01E-28	0
	TN109_ERR037319	0	8.89E-29	0
	TN10_ERR037294	0	6.36E-30	0
	TN121_ERR037320	0	1.80E-29	0
	TN124_ERR037321	0	9.73E-30	0
	TN126_ERR037322	0	7.04E-30	0
	TN128_ERR037323	0	5.02E-27	0
	TN129_ERR037324	0	3.52E-27	0
	TN12_ERR037302	0	2.55E-29	0
	TN138_ERR037325	0	1.14E-28	0
	TN149_ERR037326	0	1.46E-28	0
	TN161_ERR037327	0	9.69E-29	0
	TN163_ERR037328	0	1.01E-29	0
	TN170_ERR037329	0	1.81E-25	0
	TN171_ERR037332	0	7.92E-30	0
	TN172_ERR037333	0	9.27E-30	0
	TN176_ERR037330	0	2.84E-31	0
	TN177_ERR037331	0	3.08E-29	0
	TN24_ERR037299	0	6.68E-29	0
	TN28_ERR037295	0	5.33E-30	0
	TN30_ERR037305	0	2.17E-27	0
	TN31_ERR037303	0	2.18E-28	0

	TN38_ERR037304	0	5.06E-27	0
	TN44_ERR037306	0	1.82E-24	0
	TN48_ERR037307	0	4.62E-30	0
	TN51_ERR037308	0	1.18E-28	0
	TN57_ERR037309	0	2.71E-30	0
	TN72_ERR037312	0	1.13E-29	0
	TN76_ERR037311	0	3.09E-29	0
	TN77_ERR037310	0	1.21E-29	0
	TN85_ERR037315	0	7.52E-30	0
	TN88_ERR037313	0	2.45E-25	0
	TN90_ERR037316	0	2.04E-26	0
	Tapidor_ERR037334	0	5.07E-29	0
BnaC02g07870.1D2	Darmor_AUP_AOSW_2_D09BTACXX.I	404	5.732407	5.206708
	Darmor_AUP_BOSW_3_D09BTACXX.I	417	7.148917	6.491246
	Darmor_AUP_DOSW_2_D09BTACXX.I	673	8.372936	7.733028
	Darmor_AUP_FOSW_4_D09BTACXX.II	303	3.94783	3.429566
	Darmor_AUP_HOSW_5_D09BTACXX.I	245	4.157927	3.678678
	Darmor_AUP_IAOSW_1_1_C0UW4AC	1866	8.983334	8.712857
	Darmor_AUP_KAOSW_2_1_C0UW4A	1699	8.04525	7.748859
	Darmor_AUP_LAOSW_3_1_C0UW4AC	903	4.686494	4.401937
	Darmor_AUP_MAOSW_4_1_C0UW4A	974	4.611447	4.396382
	Darmor_AUP_NAOSW_5_1_C0UW4A	1029	5.06641	4.876918
	Darmor_AUP_PAOSW_6_1_C0UW4AC	1696	9.287854	8.934726
	Ningyou_ERR037335	0	2.31E-23	0
	TN04_ERR037297	1	0.1125323	0.109829
	TN05_ERR037298	1	0.08271558	0.03188445
	TN08_ERR037301	1	0.01670084	0.007658951
	TN103_ERR037317	2	0.03805194	0.03540744
	TN108_ERR037318	0	3.12E-23	0
	TN109_ERR037319	0	1.35E-24	0
	TN10_ERR037294	4	0.05762696	0.04361184
	TN121_ERR037320	0	3.85E-23	0
	TN124_ERR037321	1	0.01347458	0.0133388

	TN126_ERR037322	0	2.06E-26	0
	TN128_ERR037323	0	1.59E-24	0
	TN129_ERR037324	0	8.93E-20	0
	TN12_ERR037302	3	0.1081729	0.05902603
	TN138_ERR037325	1	0.01728319	0.007341413
	TN149_ERR037326	0	9.90E-19	0
	TN161_ERR037327	0	3.19E-20	0
	TN163_ERR037328	1	0.01645777	0.01627442
	TN170_ERR037329	4	0.08653938	0.05667078
	TN171_ERR037332	2	0.02648701	0
	TN172_ERR037333	1	0.01304411	0.009021368
	TN176_ERR037330	0	1.47E-21	0
	TN177_ERR037331	1	0.0148038	0.009849841
	TN24_ERR037299	0	3.33E-21	0
	TN28_ERR037295	2	0.02669717	0.02113863
	TN30_ERR037305	1	0.01518493	0
	TN31_ERR037303	0	2.12E-18	0
	TN38_ERR037304	1	0.01578563	0.01499919
	TN44_ERR037306	0	7.69E-24	0
	TN48_ERR037307	0	1.42E-19	0
	TN51_ERR037308	0	3.52E-19	0
	TN57_ERR037309	1	0.01393272	0.01381864
	TN72_ERR037312	2	0.03378543	0.02890956
	TN76_ERR037311	0	1.90E-21	0
	TN77_ERR037310	2	0.1636969	0.1611449
	TN85_ERR037315	2	0.03287135	0.03249297
	TN88_ERR037313	1	0.01395762	0.01331493
	TN90_ERR037316	1	0.01559259	0.01559126
	Tapidor_ERR037334	3	0.04510741	0.04449277
BnaU01g20600.1D2	Darmor_AUP_AOSW_2_D09BTACXX.I	83	2.14806	2.147649
	Darmor_AUP_BOSW_3_D09BTACXX.I	29	1.143235	0.9053996
	Darmor_AUP_DOSW_2_D09BTACXX.I	4	0.1294055	0.1293968
	Darmor_AUP_FOSW_4_D09BTACXX.II	3	0.1409379	0.1409379

	Darmor_AUP_HOSW_5_D09BTACXX.I	51	2.084793	2.020895
	Darmor_AUP_IAOSW_1_1_C0UW4A(	58	0.5280138	0.3271252
	Darmor_AUP_KAOSW_2_1_C0UW4A	118	1.151167	1.047258
	Darmor_AUP_LAOSW_3_1_C0UW4A(	16	0.1460524	0.1460524
	Darmor_AUP_MAOSW_4_1_C0UW4A	23	0.2076184	0.2076184
	Darmor_AUP_NAOSW_5_1_C0UW4A	17	0.1726575	0.1610737
	Darmor_AUP_PAOSW_6_1_C0UW4A(	64	0.6320568	0.6184237
	Ningyou_ERR037335	2	0.07653948	0.07653948
	TN04_ERR037297	1	0.032976	0.02806718
	TN05_ERR037298	2	0.0960444	0.0960444
	TN08_ERR037301	3	0.09281514	0.07646485
	TN103_ERR037317	0	0	0
	TN108_ERR037318	0	0.02102005	0
	TN109_ERR037319	3	0.1065449	0.1065449
	TN10_ERR037294	2	0.0868222	0.0868222
	TN121_ERR037320	0	1.47E-07	0
	TN124_ERR037321	0	7.28E-05	0
	TN126_ERR037322	1	0.0294409	0.0294409
	TN128_ERR037323	0	1.32E-07	0
	TN129_ERR037324	5	0.1432812	0.05850104
	TN12_ERR037302	1	0.09385894	0.09385894
	TN138_ERR037325	0	0	0
	TN149_ERR037326	0	0.07943035	0.07838169
	TN161_ERR037327	0	0	0
	TN163_ERR037328	1	0.03304687	0.03304687
	TN170_ERR037329	1	0.0297388	0.0297388
	TN171_ERR037332	1	0.02822869	0.02621378
	TN172_ERR037333	1	0.02929452	0.02929452
	TN176_ERR037330	1	0.02793723	0.02793723
	TN177_ERR037331	1	0.03025684	0.02704877
	TN24_ERR037299	0	1.13E-08	0
	TN28_ERR037295	5	0.1407271	0.1293878
	TN30_ERR037305	0	0	0

	TN31_ERR037303	3	0.1004561	0.100456
	TN38_ERR037304	1	0.03212267	0.03212267
	TN44_ERR037306	0	7.74E-10	7.72E-10
	TN48_ERR037307	0	0.03603583	0.0008124507
	TN51_ERR037308	2	0.1333305	0
	TN57_ERR037309	1	0.05916306	0.05912761
	TN72_ERR037312	0	0.07165717	0
	TN76_ERR037311	0	1.64E-10	0
	TN77_ERR037310	0	3.09E-29	0
	TN85_ERR037315	0	1.60E-08	1.60E-08
	TN88_ERR037313	0	1.12E-34	0
	TN90_ERR037316	0	4.26E-08	0
	Tapidor_ERR037334	0	2.56E-32	0
BnaC03g06560.1D2	Darmor_AUP_AOSW_2_D09BTACXX.I	131	6.43214	5.32354
	Darmor_AUP_BOSW_3_D09BTACXX.I	190	11.30288	9.484012
	Darmor_AUP_DOSW_2_D09BTACXX.I	416	14.82213	13.34231
	Darmor_AUP_FOSW_4_D09BTACXX.I	204	8.044157	6.773474
	Darmor_AUP_HOSW_5_D09BTACXX.I	143	6.157409	4.883131
	Darmor_AUP_IAOSW_1_1_C0UW4A(	415	6.708562	6.300674
	Darmor_AUP_KAOSW_2_1_C0UW4A	378	6.018853	5.600244
	Darmor_AUP_LAOSW_3_1_C0UW4A(	752	10.9013	10.29165
	Darmor_AUP_MAOSW_4_1_C0UW4/	730	11.56132	11.46821
	Darmor_AUP_NAOSW_5_1_C0UW4A	632	9.115113	8.730929
	Darmor_AUP_PAOSW_6_1_C0UW4A(	425	6.357645	6.073942
	Ningyou_ERR037335	18	1.24213	0.8748249
	TN04_ERR037297	0	1.30E-25	0
	TN05_ERR037298	0	4.13E-26	0
	TN08_ERR037301	0	6.04E-19	0
	TN103_ERR037317	0	3.01E-20	0
	TN108_ERR037318	0	1.66E-25	0
	TN109_ERR037319	0	2.58E-25	0
	TN10_ERR037294	0	6.09E-26	0
	TN121_ERR037320	0	4.15E-26	0

	TN124_ERR037321	0	1.15E-26	0
	TN126_ERR037322	18	1.515023	1.359452
	TN128_ERR037323	0	1.24E-26	0
	TN129_ERR037324	0	3.94E-22	0
	TN12_ERR037302	0	5.06E-23	0
	TN138_ERR037325	0	3.34E-16	0
	TN149_ERR037326	0	2.42E-25	0
	TN161_ERR037327	2	0.1480072	0
	TN163_ERR037328	15	2.092262	0.8223218
	TN170_ERR037329	0	1.47E-25	0
	TN171_ERR037332	0	6.98E-26	0
	TN172_ERR037333	0	3.63E-21	0
	TN176_ERR037330	0	1.27E-18	0
	TN177_ERR037331	0	9.27E-26	0
	TN24_ERR037299	0	1.54E-23	0
	TN28_ERR037295	0	1.56E-26	0
	TN30_ERR037305	0	1.06E-22	0
	TN31_ERR037303	0	8.35E-18	0
	TN38_ERR037304	0	9.85E-21	0
	TN44_ERR037306	0	9.41E-20	0
	TN48_ERR037307	0	6.02E-17	0
	TN51_ERR037308	12	2.514927	1.391584
	TN57_ERR037309	18	1.353372	0.2611251
	TN72_ERR037312	0	1.12E-17	0
	TN76_ERR037311	0	1.86E-25	0
	TN77_ERR037310	0	1.34E-24	0
	TN85_ERR037315	0	1.83E-24	0
	TN88_ERR037313	0	2.87E-25	0
	TN90_ERR037316	0	9.52E-26	0
	Tapidor_ERR037334	0	1.22E-19	0
BnaA09g37250.1D2	Darmor_AUP_AOSW_2_D09BTACXX.I	0	0	0
	Darmor_AUP_BOSW_3_D09BTACXX.I	0	0.9798045	0
	Darmor_AUP_DOSW_2_D09BTACXX.I	0	0	0

	Darmor_AUP_FOSW_4_D09BTACXX.I	0	6.00E-35	0
	Darmor_AUP_HOSW_5_D09BTACXX.I	0	0	0
	Darmor_AUP_IAOSW_1_1_C0UW4A	0	0.2716188	0.2716144
	Darmor_AUP_KAOSW_2_1_C0UW4A	3	0.8459328	0.8459328
	Darmor_AUP_LAOSW_3_1_C0UW4A	0	0	0
	Darmor_AUP_MAOSW_4_1_C0UW4	0	0	0
	Darmor_AUP_NAOSW_5_1_C0UW4A	0	0	0
	Darmor_AUP_PAOSW_6_1_C0UW4A	1	1.635382	1.366071
	Ningyou_ERR037335	0	3.048798	0
	TN04_ERR037297	0	2.79E-30	0
	TN05_ERR037298	0	5.20E-29	0
	TN08_ERR037301	0	1.38E-15	0
	TN103_ERR037317	0	4.94E-07	0
	TN108_ERR037318	0	7.68E-07	0
	TN109_ERR037319	0	0.1046047	0
	TN10_ERR037294	0	5.09E-11	0
	TN121_ERR037320	0	6.307374	0
	TN124_ERR037321	0	1.38E-29	0
	TN126_ERR037322	0	0.6483557	0
	TN128_ERR037323	0	1.13E-29	0
	TN129_ERR037324	0	6.33E-19	0
	TN12_ERR037302	0	1.66E-17	0
	TN138_ERR037325	0	0.02961953	0
	TN149_ERR037326	0	8.30E-30	0
	TN161_ERR037327	0	4.31E-29	0
	TN163_ERR037328	0	2.73E-13	0
	TN170_ERR037329	0	5.43E-28	0
	TN171_ERR037332	0	7.97E-19	0
	TN172_ERR037333	0	1.22E-31	0
	TN176_ERR037330	0	4.43E-29	0
	TN177_ERR037331	0	3.15E-27	0
	TN24_ERR037299	0	1.84E-27	0
	TN28_ERR037295	0	8.53E-28	0

	TN30_ERR037305	0	5.92E-30	0
	TN31_ERR037303	0	1.26E-30	0
	TN38_ERR037304	0	5.01E-29	0
	TN44_ERR037306	0	2.41E-28	0
	TN48_ERR037307	0	1.98E-18	0
	TN51_ERR037308	0	7.93E-25	0
	TN57_ERR037309	0	1.71E-05	0
	TN72_ERR037312	0	9.01E-15	0
	TN76_ERR037311	0	7.76E-30	0
	TN77_ERR037310	0	3.01E-31	0
	TN85_ERR037315	0	7.80E-24	0
	TN88_ERR037313	0	1.01E-24	0
	TN90_ERR037316	0	1.20E-28	0
	Tapidor_ERR037334	0	5.07E-30	0
BnaC01g32710.1D2	Darmor_AUP_AOSW_2_D09BTACXX.I	10	45.60932	19.47794
	Darmor_AUP_BOSW_3_D09BTACXX.I	9	40.23079	7.163684
	Darmor_AUP_DOSW_2_D09BTACXX.I	10	31.1767	16.39007
	Darmor_AUP_FOSW_4_D09BTACXX.II	5	14.23264	0
	Darmor_AUP_HOSW_5_D09BTACXX.I	10	30.69341	4.492994
	Darmor_AUP_IAOSW_1_1_C0UW4A(	23	8.993292	2.284806
	Darmor_AUP_KAOSW_2_1_C0UW4A	24	14.78101	14.05177
	Darmor_AUP_LAOSW_3_1_C0UW4A(	7	3.829686	3.786799
	Darmor_AUP_MAOSW_4_1_C0UW4A	7	4.959592	3.98354
	Darmor_AUP_NAOSW_5_1_C0UW4A	3	1.198669	1.195234
	Darmor_AUP_PAOSW_6_1_C0UW4A(	22	14.17843	10.4131
	Ningyou_ERR037335	5	7.763105	7.623594
	TN04_ERR037297	7	11.7043	11.70365
	TN05_ERR037298	5	7.243192	5.205047
	TN08_ERR037301	6	10.69937	10.44604
	TN103_ERR037317	2	3.3399	3.339888
	TN108_ERR037318	3	4.859576	2.115402
	TN109_ERR037319	0	7.24E-12	0
	TN10_ERR037294	3	3.442703	0.5914118

	TN121_ERR037320	0	6.01E-25	0
	TN124_ERR037321	0	1.89E-09	0
	TN126_ERR037322	0	3.96E-21	0
	TN128_ERR037323	4	4.55606	4.45088
	TN129_ERR037324	2	2.409566	1.689883
	TN12_ERR037302	0	1.10E-14	0
	TN138_ERR037325	0	9.00E-07	0
	TN149_ERR037326	2	3.424172	3.024232
	TN161_ERR037327	3	4.007111	1.520165
	TN163_ERR037328	4	5.288968	5.267876
	TN170_ERR037329	0	4.45E-06	0
	TN171_ERR037332	3	10.35699	10.02284
	TN172_ERR037333	0	1.02E-17	0
	TN176_ERR037330	3	8.745892	8.171959
	TN177_ERR037331	0	8.23E-24	0
	TN24_ERR037299	0	1.04E-05	0
	TN28_ERR037295	3	3.338107	2.668959
	TN30_ERR037305	3	3.959038	0
	TN31_ERR037303	4	8.970282	8.742791
	TN38_ERR037304	0	3.745081	0
	TN44_ERR037306	2	5.41656	0.8674973
	TN48_ERR037307	0	1.01E-26	0
	TN51_ERR037308	0	2.40E-14	0
	TN57_ERR037309	2	2.275054	2.255622
	TN72_ERR037312	3	4.047446	2.919903
	TN76_ERR037311	2	8.500907	0
	TN77_ERR037310	1	1.227511	0.9717611
	TN85_ERR037315	0	0.6167611	0
	TN88_ERR037313	3	3.590229	2.590294
	TN90_ERR037316	2	8.867248	0.4209298
	Tapidor_ERR037334	0	8.82E-08	0
BnaU01g27050.1D2	Darmor_AUP_AOSW_2_D09BTACXX.I	32	14.52563	14.10013
	Darmor_AUP_BOSW_3_D09BTACXX.I	18	10.74814	1.450098

	Darmor_AUP_DOSW_2_D09BTACXX.I	10	4.056813	3.554745
	Darmor_AUP_FOSW_4_D09BTACXX.I	26	12.70644	12.68829
	Darmor_AUP_HOSW_5_D09BTACXX.I	24	14.60099	3.341353
	Darmor_AUP_IAOSW_1_1_C0UW4AC	55	8.49362	8.426262
	Darmor_AUP_KAOSW_2_1_C0UW4A	38	6.283828	6.283827
	Darmor_AUP_LAOSW_3_1_C0UW4AC	13	2.108976	2.108976
	Darmor_AUP_MAOSW_4_1_C0UW4A	26	4.266244	4.266244
	Darmor_AUP_NAOSW_5_1_C0UW4A	13	2.168516	2.164057
	Darmor_AUP_PAOSW_6_1_C0UW4AC	43	7.22887	6.324287
	Ningyou_ERR037335	4	3.14716	3.143164
	TN04_ERR037297	0	2.77E-28	0
	TN05_ERR037298	0	7.72E-29	0
	TN08_ERR037301	0	4.24E-32	0
	TN103_ERR037317	0	9.04E-30	0
	TN108_ERR037318	0	3.92E-29	0
	TN109_ERR037319	0	3.70E-30	0
	TN10_ERR037294	0	6.21E-27	0
	TN121_ERR037320	0	1.00E-30	0
	TN124_ERR037321	0	7.54E-32	0
	TN126_ERR037322	0	9.49E-29	0
	TN128_ERR037323	0	9.55E-27	0
	TN129_ERR037324	0	6.17E-28	0
	TN12_ERR037302	0	4.47E-29	0
	TN138_ERR037325	0	8.23E-28	0
	TN149_ERR037326	0	1.64E-29	0
	TN161_ERR037327	6	6.916462	6.353054
	TN163_ERR037328	9	12.95245	12.03907
	TN170_ERR037329	0	1.89E-28	0
	TN171_ERR037332	0	1.05E-25	0
	TN172_ERR037333	0	5.43E-29	0
	TN176_ERR037330	0	1.29E-31	0
	TN177_ERR037331	0	6.54E-31	0
	TN24_ERR037299	0	1.88E-24	0

	TN28_ERR037295	0	4.21E-29	0
	TN30_ERR037305	0	5.04E-31	0
	TN31_ERR037303	12	8.204851	2.510503
	TN38_ERR037304	0	2.67E-24	0
	TN44_ERR037306	0	2.93E-28	0
	TN48_ERR037307	0	1.15E-27	0
	TN51_ERR037308	5	9.375539	1.557412
	TN57_ERR037309	10	8.454522	1.673916
	TN72_ERR037312	0	0	0
	TN76_ERR037311	0	2.60E-18	0
	TN77_ERR037310	18	16.2532	16.03436
	TN85_ERR037315	0	1.07E-29	0
	TN88_ERR037313	0	1.87E-30	0
	TN90_ERR037316	0	1.96E-17	0
	Tapidor_ERR037334	0	2.19E-23	0
BnaU01g00010.1D2	Darmor_AUP_AOSW_2_D09BTACXX.I	57	16.89339	14.23399
	Darmor_AUP_BOSW_3_D09BTACXX.I	54	21.28361	18.14677
	Darmor_AUP_DOSW_2_D09BTACXX.I	83	22.28985	22.15614
	Darmor_AUP_FOSW_4_D09BTACXX.II	67	21.75456	21.7514
	Darmor_AUP_HOSW_5_D09BTACXX.I	51	22.83198	22.45979
	Darmor_AUP_IAOSW_1_1_C0UW4AC	26	3.53451	3.53451
	Darmor_AUP_KAOSW_2_1_C0UW4A	26	3.521484	3.521484
	Darmor_AUP_LAOSW_3_1_C0UW4AC	37	4.810671	4.810671
	Darmor_AUP_MAOSW_4_1_C0UW4A	44	5.950598	5.950598
	Darmor_AUP_NAOSW_5_1_C0UW4A	20	2.716894	2.716894
	Darmor_AUP_PAOSW_6_1_C0UW4AC	36	4.764784	4.513294
	Ningyou_ERR037335	8	4.37469	4.075084
	TN04_ERR037297	8	3.270481	3.270467
	TN05_ERR037298	1	0.3940869	0.3925269
	TN08_ERR037301	6	3.481892	2.671394
	TN103_ERR037317	7	3.604755	3.249857
	TN108_ERR037318	0	0	0
	TN109_ERR037319	1	0.4510819	0.4510819

	TN10_ERR037294	4	1.840247	1.758603
	TN121_ERR037320	6	2.558156	0
	TN124_ERR037321	6	2.266284	2.194984
	TN126_ERR037322	0	8.70E-25	0
	TN128_ERR037323	0	3.96E-18	0
	TN129_ERR037324	6	4.028042	2.28594
	TN12_ERR037302	8	6.457431	3.132876
	TN138_ERR037325	2	0.8942193	0.8942193
	TN149_ERR037326	0	4.14E-24	0
	TN161_ERR037327	0	2.84E-23	0
	TN163_ERR037328	1	0.4212436	0.4212435
	TN170_ERR037329	0	2.34E-25	0
	TN171_ERR037332	12	5.92286	1.688594
	TN172_ERR037333	6	5.114919	1.79812
	TN176_ERR037330	16	6.64224	6.508261
	TN177_ERR037331	2	0.825727	0.8237657
	TN24_ERR037299	0	2.73E-15	0
	TN28_ERR037295	6	4.033558	0.6528034
	TN30_ERR037305	6	3.015295	1.248812
	TN31_ERR037303	4	2.159996	0
	TN38_ERR037304	4	1.82229	1.82229
	TN44_ERR037306	3	4.499432	4.367528
	TN48_ERR037307	1	0.489901	0.4694728
	TN51_ERR037308	1	0.5536002	0.5536002
	TN57_ERR037309	7	5.119279	4.545407
	TN72_ERR037312	0	3.38E-25	0
	TN76_ERR037311	0	1.90E-23	0
	TN77_ERR037310	14	5.690272	5.599833
	TN85_ERR037315	5	2.252556	2.252556
	TN88_ERR037313	0	3.34E-24	0
	TN90_ERR037316	13	5.876938	5.876938
	Tapidor_ERR037334	0	9.37E-25	0
BnaC01g08040.1D2	Darmor_AUP_AOSW_2_D09BTACXX.I	0	0	0

	Darmor_AUP_BOSW_3_D09BTACXX.I	0	0	0
	Darmor_AUP_DOSW_2_D09BTACXX.I	0	1.10E-35	0
	Darmor_AUP_FOSW_4_D09BTACXX.II	0	0	0
	Darmor_AUP_HOSW_5_D09BTACXX.I	3	0.4381303	0.4381303
	Darmor_AUP_IAOSW_1_1_C0UW4AC	0	0	0
	Darmor_AUP_KAOSW_2_1_C0UW4A	0	0	0
	Darmor_AUP_LAOSW_3_1_C0UW4AC	0	0	0
	Darmor_AUP_MAOSW_4_1_C0UW4A	0	0	0
	Darmor_AUP_NAOSW_5_1_C0UW4A	0	0	0
	Darmor_AUP_PAOSW_6_1_C0UW4AC	0	0	0
	Ningyou_ERR037335	1	0.1113681	0.1113675
	TN04_ERR037297	0	0	0
	TN05_ERR037298	3	0.30046	0.2700546
	TN08_ERR037301	0	3.34E-29	0
	TN103_ERR037317	0	1.92E-21	0
	TN108_ERR037318	0	1.18E-25	0
	TN109_ERR037319	6	0.6667627	0.6575873
	TN10_ERR037294	1	0.08013128	0.08013126
	TN121_ERR037320	0	4.88E-09	4.88E-09
	TN124_ERR037321	4	0.3438116	0.325762
	TN126_ERR037322	1	0.2001569	0
	TN128_ERR037323	1	0.215726	0.1615087
	TN129_ERR037324	1	1.140965	1.140965
	TN12_ERR037302	0	1.96E-40	0
	TN138_ERR037325	1	0.1184064	0.1010027
	TN149_ERR037326	1	0.1697238	0
	TN161_ERR037327	0	2.89E-13	0
	TN163_ERR037328	0	6.39E-32	0
	TN170_ERR037329	2	0.8733552	0
	TN171_ERR037332	0	0	0
	TN172_ERR037333	0	0	0
	TN176_ERR037330	3	0.4715789	0.3873104
	TN177_ERR037331	7	0.6206517	0.3804154

	TN24_ERR037299	0	8.07E-10	8.07E-10
	TN28_ERR037295	0	1.72E-18	0
	TN30_ERR037305	2	0.18326	0.183136
	TN31_ERR037303	0	0	0
	TN38_ERR037304	1	1.195702	1.195702
	TN44_ERR037306	0	9.72E-61	0
	TN48_ERR037307	0	0	0
	TN51_ERR037308	0	0	0
	TN57_ERR037309	1	0.08587175	0.08558714
	TN72_ERR037312	1	0.1062855	0.1056346
	TN76_ERR037311	1	0.09729641	0.09411323
	TN77_ERR037310	0	2.77E-56	0
	TN85_ERR037315	1	0.1102883	0.1102883
	TN88_ERR037313	0	3.29E-30	0
	TN90_ERR037316	0	1.17E-48	0
	Tapidor_ERR037334	0	2.01E-38	0
BnaA03g42070.1D2	Darmor_AUP_AOSW_2_D09BTACXX.I	310	13.87459	11.71318
	Darmor_AUP_BOSW_3_D09BTACXX.I	366	23.13998	20.55571
	Darmor_AUP_DOSW_2_D09BTACXX.I	226	10.92221	9.257408
	Darmor_AUP_FOSW_4_D09BTACXX.II	282	14.92563	12.57737
	Darmor_AUP_HOSW_5_D09BTACXX.I	138	8.44345	6.761217
	Darmor_AUP_IAOSW_1_1_C0UW4AC	202	3.929923	3.562807
	Darmor_AUP_KAOSW_2_1_C0UW4A	326	6.180646	5.32504
	Darmor_AUP_LAOSW_3_1_C0UW4AC	424	7.852005	6.983477
	Darmor_AUP_MAOSW_4_1_C0UW4A	406	6.859899	6.085336
	Darmor_AUP_NAOSW_5_1_C0UW4A	424	8.853794	7.654532
	Darmor_AUP_PAOSW_6_1_C0UW4AC	236	3.700042	3.000234
	Ningyou_ERR037335	6	1.542157	0.5105437
	TN04_ERR037297	5	0.2782949	0
	TN05_ERR037298	12	0.8550619	0.4226473
	TN08_ERR037301	11	1.040885	0.2822341
	TN103_ERR037317	5	1.188783	0.06339425
	TN108_ERR037318	6	0.6209235	0

	TN109_ERR037319	9	0.5970869	0.1033009
	TN10_ERR037294	0	1.14E-13	0
	TN121_ERR037320	0	3.50E-25	0
	TN124_ERR037321	0	6.56E-15	0
	TN126_ERR037322	14	0.6896191	0.2663024
	TN128_ERR037323	15	1.181841	0.7759513
	TN129_ERR037324	0	7.93E-26	0
	TN12_ERR037302	10	1.074137	0.1276415
	TN138_ERR037325	0	1.67E-20	0
	TN149_ERR037326	7	0.623453	0.5813006
	TN161_ERR037327	0	1.41E-22	0
	TN163_ERR037328	0	2.53E-28	0
	TN170_ERR037329	0	3.67E-17	0
	TN171_ERR037332	10	0.5059801	0.3062754
	TN172_ERR037333	0	4.71E-18	0
	TN176_ERR037330	18	1.308941	0.5674336
	TN177_ERR037331	13	0.6239321	0.4508933
	TN24_ERR037299	0	3.72E-19	0
	TN28_ERR037295	0	9.25E-20	0
	TN30_ERR037305	9	0.4689576	0.282121
	TN31_ERR037303	0	4.93E-25	0
	TN38_ERR037304	8	1.534364	0.6122646
	TN44_ERR037306	10	0.7199252	0.3576413
	TN48_ERR037307	0	1.20E-21	0
	TN51_ERR037308	4	0.6593488	0
	TN57_ERR037309	14	0.6521228	0.1295273
	TN72_ERR037312	0	4.72E-16	0
	TN76_ERR037311	11	0.9682338	0.4422818
	TN77_ERR037310	10	0.68498	0.1078537
	TN85_ERR037315	6	0.3252517	0
	TN88_ERR037313	0	8.28E-27	0
	TN90_ERR037316	9	0.440333	0
	Tapidor_ERR037334	0	1.01E-30	0

BnaC02g03200.1D2	Darmor_AUP_AOSW_2_D09BTACXX.I	323	24.14863	20.52673
	Darmor_AUP_BOSW_3_D09BTACXX.I	164	16.35564	12.901
	Darmor_AUP_DOSW_2_D09BTACXX.I	544	40.7054	36.46555
	Darmor_AUP_FOSW_4_D09BTACXX.II	153	13.31051	10.06681
	Darmor_AUP_HOSW_5_D09BTACXX.I	249	27.7455	23.21506
	Darmor_AUP_IAOSW_1_1_C0UW4AC	1638	29.6437	27.77685
	Darmor_AUP_KAOSW_2_1_C0UW4A	1463	30.54292	28.72001
	Darmor_AUP_LAOSW_3_1_C0UW4AC	712	15.85615	14.22919
	Darmor_AUP_MAOSW_4_1_C0UW4A	733	16.17898	14.74827
	Darmor_AUP_NAOSW_5_1_C0UW4A	678	15.09623	13.75889
	Darmor_AUP_PAOSW_6_1_C0UW4AC	1722	37.07507	34.84773
	Ningyou_ERR037335	76	15.33019	10.43524
	TN04_ERR037297	62	8.52559	5.508439
	TN05_ERR037298	116	12.58993	9.41402
	TN08_ERR037301	29	7.960532	4.406987
	TN103_ERR037317	103	16.1324	12.69225
	TN108_ERR037318	95	15.79788	11.5149
	TN109_ERR037319	69	10.47502	7.527378
	TN10_ERR037294	94	10.46656	7.353844
	TN121_ERR037320	59	6.992492	4.752434
	TN124_ERR037321	60	6.298082	4.200151
	TN126_ERR037322	99	10.68298	8.542374
	TN128_ERR037323	0	1.35E-14	0
	TN129_ERR037324	97	9.192431	6.545536
	TN12_ERR037302	43	7.445472	4.896508
	TN138_ERR037325	77	11.1499	7.857327
	TN149_ERR037326	41	6.45427	3.233866
	TN161_ERR037327	40	7.361845	4.551002
	TN163_ERR037328	127	10.59149	8.447711
	TN170_ERR037329	148	18.57282	14.5982
	TN171_ERR037332	0	3.13E-20	0
	TN172_ERR037333	118	14.11081	10.84282
	TN176_ERR037330	108	11.5324	8.922754

	TN177_ERR037331	26	4.750847	2.652853
	TN24_ERR037299	0	4.44E-20	0
	TN28_ERR037295	100	16.85638	12.59386
	TN30_ERR037305	79	9.865137	7.129221
	TN31_ERR037303	49	9.379128	6.544448
	TN38_ERR037304	112	19.14346	14.65802
	TN44_ERR037306	41	8.623459	4.928675
	TN48_ERR037307	55	9.118292	6.646232
	TN51_ERR037308	85	14.47633	10.16158
	TN57_ERR037309	33	5.143542	2.428486
	TN72_ERR037312	35	7.188748	3.453858
	TN76_ERR037311	72	9.855454	6.958088
	TN77_ERR037310	0	6.50E-21	0
	TN85_ERR037315	62	9.937851	6.721087
	TN88_ERR037313	0	4.63E-21	0
	TN90_ERR037316	58	5.965953	3.561145
	Tapidor_ERR037334	0	1.41E-15	0
BnaC02g13740.1D2	Darmor_AUP_AOSW_2_D09BTACXX.I	252	16.91874	14.03182
	Darmor_AUP_BOSW_3_D09BTACXX.I	309	21.72115	18.62933
	Darmor_AUP_DOSW_2_D09BTACXX.I	129	6.420304	5.045753
	Darmor_AUP_FOSW_4_D09BTACXX.II	129	10.97683	8.634688
	Darmor_AUP_HOSW_5_D09BTACXX.I	138	13.13334	10.25376
	Darmor_AUP_IAOSW_1_1_C0UW4AC	888	14.43097	13.24966
	Darmor_AUP_KAOSW_2_1_C0UW4A	836	13.38694	12.27939
	Darmor_AUP_LAOSW_3_1_C0UW4AC	801	13.51669	12.32356
	Darmor_AUP_MAOSW_4_1_C0UW4A	746	12.41556	11.17894
	Darmor_AUP_NAOSW_5_1_C0UW4A	952	14.12142	12.89728
	Darmor_AUP_PAOSW_6_1_C0UW4AC	1012	14.98499	13.70597
	Ningyou_ERR037335	1	0.2674448	0.1208042
	TN04_ERR037297	0	3.83E-14	0
	TN05_ERR037298	0	3.07E-19	0
	TN08_ERR037301	0	6.41E-19	0
	TN103_ERR037317	0	6.59E-17	0

	TN108_ERR037318	0	2.27E-14	0
	TN109_ERR037319	0	5.10E-16	0
	TN10_ERR037294	0	7.90E-17	0
	TN121_ERR037320	0	2.91E-17	0
	TN124_ERR037321	0	1.94E-18	0
	TN126_ERR037322	0	7.95E-18	0
	TN128_ERR037323	0	1.61E-21	0
	TN129_ERR037324	0	1.13E-21	0
	TN12_ERR037302	0	6.34E-19	0
	TN138_ERR037325	0	4.81E-17	0
	TN149_ERR037326	0	1.11E-22	0
	TN161_ERR037327	0	2.60E-18	0
	TN163_ERR037328	0	9.74E-19	0
	TN170_ERR037329	3	0.121903	0.06763571
	TN171_ERR037332	0	5.74E-24	0
	TN172_ERR037333	0	2.54E-17	0
	TN176_ERR037330	0	9.64E-19	0
	TN177_ERR037331	0	6.13E-19	0
	TN24_ERR037299	0	9.78E-23	0
	TN28_ERR037295	0	5.27E-17	0
	TN30_ERR037305	0	3.82E-17	0
	TN31_ERR037303	0	7.93E-17	0
	TN38_ERR037304	0	2.19E-22	0
	TN44_ERR037306	0	1.75E-16	0
	TN48_ERR037307	0	2.33E-19	0
	TN51_ERR037308	0	1.74E-19	0
	TN57_ERR037309	0	2.86E-19	0
	TN72_ERR037312	0	3.96E-09	0
	TN76_ERR037311	0	4.29E-22	0
	TN77_ERR037310	0	2.93E-18	0
	TN85_ERR037315	0	3.11E-08	2.83E-08
	TN88_ERR037313	0	1.42E-12	0
	TN90_ERR037316	0	2.61E-20	0

	Tapidor_ERR037334	0	1.49E-19	0
BnaC06g05080.1D2	Darmor_AUP_AOSW_2_D09BTACXX.I	1	0.000345785	0.0003457742
	Darmor_AUP_BOSW_3_D09BTACXX.I	3	0.00208274	0.0006677359
	Darmor_AUP_DOSW_2_D09BTACXX.I	2	0.000648952	0.0006481304
	Darmor_AUP_FOSW_4_D09BTACXX.II	2	0.000914822	0.0003874085
	Darmor_AUP_HOSW_5_D09BTACXX.I	1	0.000490416	0.0004893207
	Darmor_AUP_IAOSW_1_1_C0UW4AC	0	2.86E-64	0
	Darmor_AUP_KAOSW_2_1_C0UW4A	0	2.82E-08	0
	Darmor_AUP_LAOSW_3_1_C0UW4AC	0	1.96E-41	0
	Darmor_AUP_MAOSW_4_1_C0UW4A	0	0	0
	Darmor_AUP_NAOSW_5_1_C0UW4A	1	0.000132714	0
	Darmor_AUP_PAOSW_6_1_C0UW4AC	0	0	0
	Ningyou_ERR037335	10	0.05766524	0.02851848
	TN04_ERR037297	4	0.05394427	0.02667205
	TN05_ERR037298	12	0.07743179	0.04392825
	TN08_ERR037301	4	0.02338103	0.008835847
	TN103_ERR037317	9	0.3208019	0.2503663
	TN108_ERR037318	8	0.07264686	0.04148435
	TN109_ERR037319	4	0.1514413	0.1026751
	TN10_ERR037294	3	0.01493499	0.006912727
	TN121_ERR037320	9	0.07299799	0.04148061
	TN124_ERR037321	7	0.07154303	0.03731354
	TN126_ERR037322	10	0.05895706	0.02746995
	TN128_ERR037323	3	0.06032942	0.03291277
	TN129_ERR037324	5	0.06220022	0.03315986
	TN12_ERR037302	4	0.02105141	0.003016851
	TN138_ERR037325	10	0.1771083	0.1293999
	TN149_ERR037326	10	0.2196868	0.1714384
	TN161_ERR037327	3	0.0243011	0.009056532
	TN163_ERR037328	3	0.03461063	0.01534717
	TN170_ERR037329	4	0.02739493	0.007777531
	TN171_ERR037332	1	0.02870705	0.008531969
	TN172_ERR037333	8	0.03272611	0.01633661

	TN176_ERR037330	5	0.03468817	0.01780936
	TN177_ERR037331	4	0.03290566	0.009968383
	TN24_ERR037299	3	0.06501955	0.02767358
	TN28_ERR037295	8	0.06098946	0.03151956
	TN30_ERR037305	3	0.02255487	0.001675346
	TN31_ERR037303	5	0.04650896	0.01669218
	TN38_ERR037304	5	0.03794925	0.001021299
	TN44_ERR037306	1	0.0190912	0.0004265702
	TN48_ERR037307	4	0.01530031	0.004731807
	TN51_ERR037308	4	0.01742397	0.001161842
	TN57_ERR037309	4	0.05941219	0.03125724
	TN72_ERR037312	2	0.08842362	0.05054254
	TN76_ERR037311	5	0.03685848	0.01200453
	TN77_ERR037310	7	0.07734435	0.04252457
	TN85_ERR037315	1	0.06446031	0.03381408
	TN88_ERR037313	14	0.07809491	0.0449852
	TN90_ERR037316	3	0.06251411	0.03489153
	Tapidor_ERR037334	2	0.09488558	0.04988895
BnaC06g26930.1D2	Darmor_AUP_AOSW_2_D09BTACXX.I	42	6.024166	6.024166
	Darmor_AUP_BOSW_3_D09BTACXX.I	109	22.2436	22.2426
	Darmor_AUP_DOSW_2_D09BTACXX.I	87	11.54222	11.54222
	Darmor_AUP_FOSW_4_D09BTACXX.II	3	0.6323044	0.6322505
	Darmor_AUP_HOSW_5_D09BTACXX.I	23	4.521988	4.521885
	Darmor_AUP_IAOSW_1_1_C0UW4AC	94	6.067059	6.067059
	Darmor_AUP_KAOSW_2_1_C0UW4A	68	4.380098	4.380098
	Darmor_AUP_LAOSW_3_1_C0UW4AC	7	0.4577862	0.4577862
	Darmor_AUP_MAOSW_4_1_C0UW4A	9	0.5933954	0.5933954
	Darmor_AUP_NAOSW_5_1_C0UW4A	11	0.7369776	0.7369776
	Darmor_AUP_PAOSW_6_1_C0UW4AC	83	5.540932	5.540932
	Ningyou_ERR037335	5	1.083126	1.082778
	TN04_ERR037297	2	0.4248272	0.424823
	TN05_ERR037298	17	3.51827	3.51769
	TN08_ERR037301	5	2.12712	2.127066

	TN103_ERR037317	8	2.005604	0.9322722
	TN108_ERR037318	0	3.75E-33	0
	TN109_ERR037319	20	4.241553	4.241553
	TN10_ERR037294	3	0.5737995	0.563137
	TN121_ERR037320	1	0.2063388	0.2062831
	TN124_ERR037321	11	1.819175	1.819128
	TN126_ERR037322	7	1.202328	1.19887
	TN128_ERR037323	1	1.126866	0
	TN129_ERR037324	20	4.293755	4.14577
	TN12_ERR037302	14	3.173064	3.172969
	TN138_ERR037325	7	1.580075	1.580019
	TN149_ERR037326	3	0.7562615	0.7554718
	TN161_ERR037327	2	0.4879369	0.4879015
	TN163_ERR037328	12	3.273012	3.272674
	TN170_ERR037329	2	0.3351736	0.3351736
	TN171_ERR037332	7	1.144469	1.144462
	TN172_ERR037333	6	1.84679	1.843674
	TN176_ERR037330	0	4.00E-42	0
	TN177_ERR037331	0	2.73E-26	0
	TN24_ERR037299	0	1.83E-31	0
	TN28_ERR037295	1	0.1753931	0.175071
	TN30_ERR037305	1	0.2022565	0.2022169
	TN31_ERR037303	0	1.16E-31	0
	TN38_ERR037304	16	5.123665	5.121632
	TN44_ERR037306	4	2.284985	2.284884
	TN48_ERR037307	8	1.725869	1.72586
	TN51_ERR037308	3	0.7648061	0.7648046
	TN57_ERR037309	8	2.198483	2.197277
	TN72_ERR037312	0	1.72E-27	0
	TN76_ERR037311	7	1.435401	1.407453
	TN77_ERR037310	11	2.064091	2.063978
	TN85_ERR037315	11	2.971532	2.971285
	TN88_ERR037313	10	1.835652	1.833041

	TN90_ERR037316	6	2.043509	0
	Tapidor_ERR037334	0	1.38E-31	0
BnaC06g28350.1D2	Darmor_AUP_AOSW_2_D09BTACXX.I	0	0.04500933	0
	Darmor_AUP_BOSW_3_D09BTACXX.I	0	1.66E-34	0
	Darmor_AUP_DOSW_2_D09BTACXX.I	0	5.71E-38	0
	Darmor_AUP_FOSW_4_D09BTACXX.II	0	0	0
	Darmor_AUP_HOSW_5_D09BTACXX.I	0	0	0
	Darmor_AUP_IAOSW_1_1_C0UW4AC	0	0	0
	Darmor_AUP_KAOSW_2_1_C0UW4A	0	0	0
	Darmor_AUP_LAOSW_3_1_C0UW4AC	0	0	0
	Darmor_AUP_MAOSW_4_1_C0UW4A	0	0	0
	Darmor_AUP_NAOSW_5_1_C0UW4A	0	0	0
	Darmor_AUP_PAOSW_6_1_C0UW4AC	0	0.03281187	0
	Ningyou_ERR037335	0	0	0
	TN04_ERR037297	0	0	0
	TN05_ERR037298	0	0	0
	TN08_ERR037301	0	0	0
	TN103_ERR037317	0	0	0
	TN108_ERR037318	0	0	0
	TN109_ERR037319	0	0	0
	TN10_ERR037294	0	0	0
	TN121_ERR037320	0	0	0
	TN124_ERR037321	0	0	0
	TN126_ERR037322	0	0	0
	TN128_ERR037323	0	0	0
	TN129_ERR037324	0	0	0
	TN12_ERR037302	0	0	0
	TN138_ERR037325	0	0	0
	TN149_ERR037326	0	0	0
	TN161_ERR037327	0	0	0
	TN163_ERR037328	0	0	0
	TN170_ERR037329	0	0	0
	TN171_ERR037332	0	0	0

	TN172_ERR037333	0	0	0
	TN176_ERR037330	0	0	0
	TN177_ERR037331	0	8.86E-36	0
	TN24_ERR037299	0	2.35E-31	0
	TN28_ERR037295	0	2.91E-37	0
	TN30_ERR037305	0	2.85E-64	0
	TN31_ERR037303	0	0	0
	TN38_ERR037304	0	0	0
	TN44_ERR037306	0	0	0
	TN48_ERR037307	0	0	0
	TN51_ERR037308	0	0	0
	TN57_ERR037309	0	0	0
	TN72_ERR037312	0	1.39E-41	0
	TN76_ERR037311	0	0	0
	TN77_ERR037310	0	0	0
	TN85_ERR037315	0	1.93E-29	0
	TN88_ERR037313	0	0	0
	TN90_ERR037316	0	1.46E-41	0
	Tapidor_ERR037334	0	0	0
BnaC05g35430.1D2	Darmor_AUP_AOSW_2_D09BTACXX.I	696	28.44548	26.56889
	Darmor_AUP_BOSW_3_D09BTACXX.I	882	47.86123	44.5571
	Darmor_AUP_DOSW_2_D09BTACXX.I	1456	60.21445	57.39945
	Darmor_AUP_FOSW_4_D09BTACXX.II	73	3.691202	2.970711
	Darmor_AUP_HOSW_5_D09BTACXX.I	283	15.1004	13.74436
	Darmor_AUP_IAOSW_1_1_C0UW4AC	505	9.726243	9.008621
	Darmor_AUP_KAOSW_2_1_C0UW4A	1224	22.11923	21.10917
	Darmor_AUP_LAOSW_3_1_C0UW4A	107	1.820047	1.812142
	Darmor_AUP_MAOSW_4_1_C0UW4A	118	2.282086	1.994518
	Darmor_AUP_NAOSW_5_1_C0UW4A	80	1.651003	1.616664
	Darmor_AUP_PAOSW_6_1_C0UW4A	857	17.36716	16.30616
	Ningyou_ERR037335	3	0.7951011	0
	TN04_ERR037297	6	0.4312184	0.4289586
	TN05_ERR037298	2	0.1073312	0.1052145

	TN08_ERR037301	0	7.16E-28	0
	TN103_ERR037317	3	0.9713712	0.7643937
	TN108_ERR037318	1	0.1429599	0.1426652
	TN109_ERR037319	3	0.1802208	0.118148
	TN10_ERR037294	0	1.25E-09	0
	TN121_ERR037320	4	0.2284057	0.2275254
	TN124_ERR037321	7	1.083923	0.6795503
	TN126_ERR037322	5	1.530452	0.5200178
	TN128_ERR037323	9	0.6165052	0.004434458
	TN129_ERR037324	0	1.50E-18	0
	TN12_ERR037302	0	1.54E-06	0
	TN138_ERR037325	0	7.11E-19	0
	TN149_ERR037326	0	1.02E-28	0
	TN161_ERR037327	2	0.5895564	0
	TN163_ERR037328	0	0.001126373	0
	TN170_ERR037329	1	0.0501126	0.04805736
	TN171_ERR037332	0	1.77E-18	0
	TN172_ERR037333	1	0.05012709	0.03161499
	TN176_ERR037330	1	0.1874673	0.01564573
	TN177_ERR037331	0	3.99E-27	0
	TN24_ERR037299	0	5.56E-25	0
	TN28_ERR037295	7	0.3981557	0
	TN30_ERR037305	2	0.241498	0.160437
	TN31_ERR037303	3	0.2471678	0.1360583
	TN38_ERR037304	7	0.7480327	0.01130695
	TN44_ERR037306	4	1.318078	0.6597666
	TN48_ERR037307	4	0.5376092	0
	TN51_ERR037308	1	0.1094335	0
	TN57_ERR037309	3	0.1465897	0.006011368
	TN72_ERR037312	0	7.67E-24	0
	TN76_ERR037311	4	0.2188372	0.04896593
	TN77_ERR037310	0	1.79E-22	0
	TN85_ERR037315	0	5.09E-28	0

	TN88_ERR037313	1	0.051126	0.05112441
	TN90_ERR037316	3	0.2337521	0
	Tapidor_ERR037334	1	0.05090966	0.05065381
BnaU01g13590.1D2	Darmor_AUP_AOSW_2_D09BTACXX.I	2	0.08754792	0.08754792
	Darmor_AUP_BOSW_3_D09BTACXX.I	5	0.3048815	0.3012093
	Darmor_AUP_DOSW_2_D09BTACXX.I	0	0	0
	Darmor_AUP_FOSW_4_D09BTACXX.I	0	0	0
	Darmor_AUP_HOSW_5_D09BTACXX.I	1	0.06093064	0.06093064
	Darmor_AUP_IAOSW_1_1_C0UW4AC	6	0.1139599	0.1139596
	Darmor_AUP_KAOSW_2_1_C0UW4A	11	0.2095386	0.2095386
	Darmor_AUP_LAOSW_3_1_C0UW4AC	0	0	0
	Darmor_AUP_MAOSW_4_1_C0UW4A	0	0	0
	Darmor_AUP_NAOSW_5_1_C0UW4A	0	0	0
	Darmor_AUP_PAOSW_6_1_C0UW4AC	12	0.2366763	0.2364218
	Ningyou_ERR037335	0	5.50E-62	0
	TN04_ERR037297	0	0	0
	TN05_ERR037298	0	0	0
	TN08_ERR037301	0	0	0
	TN103_ERR037317	0	0	0
	TN108_ERR037318	0	0	0
	TN109_ERR037319	0	0	0
	TN10_ERR037294	0	8.28E-58	0
	TN121_ERR037320	0	0	0
	TN124_ERR037321	0	0	0
	TN126_ERR037322	0	0	0
	TN128_ERR037323	0	0	0
	TN129_ERR037324	0	0	0
	TN12_ERR037302	0	0	0
	TN138_ERR037325	0	0	0
	TN149_ERR037326	0	0	0
	TN161_ERR037327	0	0	0
	TN163_ERR037328	0	0	0
	TN170_ERR037329	0	0	0

	TN171_ERR037332	0	1.66E-20	0
	TN172_ERR037333	0	7.43E-50	0
	TN176_ERR037330	0	0	0
	TN177_ERR037331	0	0	0
	TN24_ERR037299	0	0	0
	TN28_ERR037295	0	4.94E-56	0
	TN30_ERR037305	0	1.86E-45	0
	TN31_ERR037303	0	0	0
	TN38_ERR037304	0	1.40E-39	0
	TN44_ERR037306	0	0	0
	TN48_ERR037307	0	0	0
	TN51_ERR037308	0	0	0
	TN57_ERR037309	0	1.63E-61	0
	TN72_ERR037312	0	0	0
	TN76_ERR037311	0	1.19E-06	0
	TN77_ERR037310	0	0	0
	TN85_ERR037315	0	0	0
	TN88_ERR037313	0	1.03E-31	0
	TN90_ERR037316	0	0	0
	Tapidor_ERR037334	0	4.60E-38	0
BnaC02g04850.1D2	Darmor_AUP_AOSW_2_D09BTACXX.I	1	0.02224464	0.02224464
	Darmor_AUP_BOSW_3_D09BTACXX.I	2	0.05971009	0.05970836
	Darmor_AUP_DOSW_2_D09BTACXX.I	0	2.12E-34	0
	Darmor_AUP_FOSW_4_D09BTACXX.II	0	0	0
	Darmor_AUP_HOSW_5_D09BTACXX.I	0	2.16E-15	0
	Darmor_AUP_IAOSW_1_1_C0UW4A(	3	0.03022559	0.03022559
	Darmor_AUP_KAOSW_2_1_C0UW4A	1	0.01015246	0.01015246
	Darmor_AUP_LAOSW_3_1_C0UW4A(	0	0	0
	Darmor_AUP_MAOSW_4_1_C0UW4/	0	0	0
	Darmor_AUP_NAOSW_5_1_C0UW4A	0	0	0
	Darmor_AUP_PAOSW_6_1_C0UW4A(	0	0	0
	Ningyou_ERR037335	0	0.2949512	0
	TN04_ERR037297	1	0.03117362	0.03117362

	TN05_ERR037298	0	3.24E-46	0
	TN08_ERR037301	0	5.40E-23	0
	TN103_ERR037317	1	0.03864063	0.03829006
	TN108_ERR037318	0	0	0
	TN109_ERR037319	0	2.29E-44	0
	TN10_ERR037294	0	3.21E-25	0
	TN121_ERR037320	1	0.03258127	0.03258127
	TN124_ERR037321	0	0	0
	TN126_ERR037322	0	1.01E-56	0
	TN128_ERR037323	0	0	0
	TN129_ERR037324	0	1.55E-48	0
	TN12_ERR037302	0	0	0
	TN138_ERR037325	0	0	0
	TN149_ERR037326	0	0	0
	TN161_ERR037327	0	5.76E-08	0
	TN163_ERR037328	0	0	0
	TN170_ERR037329	4	0.1494518	0.1494518
	TN171_ERR037332	0	1.63E-57	0
	TN172_ERR037333	0	2.04E-41	0
	TN176_ERR037330	0	3.01E-05	0
	TN177_ERR037331	1	0.02971799	0.02971799
	TN24_ERR037299	0	6.03E-39	0
	TN28_ERR037295	0	4.37E-26	0
	TN30_ERR037305	0	3.72E-34	0
	TN31_ERR037303	0	0	0
	TN38_ERR037304	0	4.96E-54	0
	TN44_ERR037306	0	0	0
	TN48_ERR037307	0	0	0
	TN51_ERR037308	0	9.20E-61	0
	TN57_ERR037309	0	0	0
	TN72_ERR037312	0	5.26E-34	0
	TN76_ERR037311	1	0.03129742	0.01709218
	TN77_ERR037310	0	0	0

	TN85_ERR037315	0	1.19E-59	0
	TN88_ERR037313	0	4.18E-55	0
	TN90_ERR037316	0	1.77E-28	0
	Tapidor_ERR037334	0	1.64E-39	0
BnaC09g11140.1D2	Darmor_AUP_AOSW_2_D09BTACXX.I	456	15.36893	14.14853
	Darmor_AUP_BOSW_3_D09BTACXX.I	970	40.73686	38.45062
	Darmor_AUP_DOSW_2_D09BTACXX.I	1083	31.07823	29.37522
	Darmor_AUP_FOSW_4_D09BTACXX.II	196	7.166115	6.392525
	Darmor_AUP_HOSW_5_D09BTACXX.I	454	21.81027	19.87503
	Darmor_AUP_IAOSW_1_1_C0UW4AC	1461	16.34008	15.81885
	Darmor_AUP_KAOSW_2_1_C0UW4A	1429	16.11985	15.76225
	Darmor_AUP_LAOSW_3_1_C0UW4AC	879	10.02931	9.955987
	Darmor_AUP_MAOSW_4_1_C0UW4A	969	11.095	10.7851
	Darmor_AUP_NAOSW_5_1_C0UW4A	966	11.30439	11.17537
	Darmor_AUP_PAOSW_6_1_C0UW4AC	1680	19.77464	19.1397
	Ningyou_ERR037335	128	10.64388	9.07213
	TN04_ERR037297	0	1.21E-21	0
	TN05_ERR037298	0	3.39E-11	0
	TN08_ERR037301	1	0.2603327	0
	TN103_ERR037317	180	13.53336	11.93907
	TN108_ERR037318	190	14.45724	12.32403
	TN109_ERR037319	301	21.44776	18.8485
	TN10_ERR037294	0	1.05E-09	1.05E-09
	TN121_ERR037320	232	14.23138	12.7769
	TN124_ERR037321	0	3.43E-24	0
	TN126_ERR037322	0	1.10E-26	0
	TN128_ERR037323	258	16.34537	14.1086
	TN129_ERR037324	0	0.01001378	0
	TN12_ERR037302	0	0.006362043	0
	TN138_ERR037325	172	12.94343	11.35981
	TN149_ERR037326	0	9.22E-25	0
	TN161_ERR037327	0	8.20E-20	0
	TN163_ERR037328	0	6.68E-23	0

	TN170_ERR037329	188	10.00785	8.171463
	TN171_ERR037332	0	6.55E-21	0
	TN172_ERR037333	0	3.22E-21	0
	TN176_ERR037330	0	1.54E-26	0
	TN177_ERR037331	187	9.657308	8.217363
	TN24_ERR037299	186	13.41973	11.26439
	TN28_ERR037295	288	15.36833	13.31593
	TN30_ERR037305	290	14.82672	12.85695
	TN31_ERR037303	0	3.98E-17	0
	TN38_ERR037304	2	0.2194495	0
	TN44_ERR037306	207	16.42605	14.06583
	TN48_ERR037307	233	9.272032	7.495343
	TN51_ERR037308	173	11.94083	9.988343
	TN57_ERR037309	272	11.08719	9.628238
	TN72_ERR037312	239	13.20341	11.55426
	TN76_ERR037311	291	15.1189	13.4342
	TN77_ERR037310	230	11.92207	10.14038
	TN85_ERR037315	248	14.37229	12.10589
	TN88_ERR037313	266	13.4337	11.78654
	TN90_ERR037316	1	0.04156247	0.03118858
	Tapidor_ERR037334	2	0.0936087	0.09253533
BnaA03g42850.1D2	Darmor_AUP_AOSW_2_D09BTACXX.I	0	0	0
	Darmor_AUP_BOSW_3_D09BTACXX.I	0	0	0
	Darmor_AUP_DOSW_2_D09BTACXX.I	0	0	0
	Darmor_AUP_FOSW_4_D09BTACXX.II	0	0	0
	Darmor_AUP_HOSW_5_D09BTACXX.I	0	0	0
	Darmor_AUP_IAOSW_1_1_C0UW4AC	0	0	0
	Darmor_AUP_KAOSW_2_1_C0UW4A	0	0	0
	Darmor_AUP_LAOSW_3_1_C0UW4A	0	0	0
	Darmor_AUP_MAOSW_4_1_C0UW4A	0	0	0
	Darmor_AUP_NAOSW_5_1_C0UW4A	0	0	0
	Darmor_AUP_PAOSW_6_1_C0UW4A	0	0	0
	Ningyou_ERR037335	0	0	0

	TN04_ERR037297	0	0	0
	TN05_ERR037298	0	0	0
	TN08_ERR037301	0	0	0
	TN103_ERR037317	0	0	0
	TN108_ERR037318	0	0	0
	TN109_ERR037319	0	0	0
	TN10_ERR037294	0	0	0
	TN121_ERR037320	0	0	0
	TN124_ERR037321	0	0	0
	TN126_ERR037322	0	0	0
	TN128_ERR037323	0	0	0
	TN129_ERR037324	0	0	0
	TN12_ERR037302	0	0	0
	TN138_ERR037325	0	0	0
	TN149_ERR037326	0	0	0
	TN161_ERR037327	0	0	0
	TN163_ERR037328	0	0	0
	TN170_ERR037329	0	0	0
	TN171_ERR037332	0	0	0
	TN172_ERR037333	0	0	0
	TN176_ERR037330	0	0	0
	TN177_ERR037331	0	0	0
	TN24_ERR037299	0	0	0
	TN28_ERR037295	0	2.13E-62	0
	TN30_ERR037305	0	0	0
	TN31_ERR037303	0	0	0
	TN38_ERR037304	0	0	0
	TN44_ERR037306	0	0	0
	TN48_ERR037307	0	0	0
	TN51_ERR037308	0	0	0
	TN57_ERR037309	0	0	0
	TN72_ERR037312	0	0	0
	TN76_ERR037311	0	0	0

	TN77_ERR037310	0	0	0
	TN85_ERR037315	0	0	0
	TN88_ERR037313	0	0	0
	TN90_ERR037316	0	0	0
	Tapidor_ERR037334	0	0	0
BnaC02g00010.1D2	Darmor_AUP_AOSW_2_D09BTACXX.I	157	4.680896	4.142316
	Darmor_AUP_BOSW_3_D09BTACXX.I	125	5.070501	4.187642
	Darmor_AUP_DOSW_2_D09BTACXX.I	76	1.999601	1.488351
	Darmor_AUP_FOSW_4_D09BTACXX.I	118	3.334655	3.108181
	Darmor_AUP_HOSW_5_D09BTACXX.I	176	7.658962	6.879841
	Darmor_AUP_IAOSW_1_1_C0UW4A	385	4.183141	3.91453
	Darmor_AUP_KAOSW_2_1_C0UW4A	441	5.148202	4.7718
	Darmor_AUP_LAOSW_3_1_C0UW4A	340	3.790865	3.48466
	Darmor_AUP_MAOSW_4_1_C0UW4	354	4.017043	3.70154
	Darmor_AUP_NAOSW_5_1_C0UW4A	269	2.878456	2.67858
	Darmor_AUP_PAOSW_6_1_C0UW4A	409	4.438149	4.399437
	Ningyou_ERR037335	8	0.3454556	0.2705881
	TN04_ERR037297	9	0.3358857	0.3119312
	TN05_ERR037298	14	0.6131378	0.1206824
	TN08_ERR037301	5	0.6011433	0.5955105
	TN103_ERR037317	13	0.8101882	0.4214662
	TN108_ERR037318	15	0.8511315	0.6469414
	TN109_ERR037319	11	0.4344936	0.23968
	TN10_ERR037294	11	0.3780354	0.34952
	TN121_ERR037320	9	0.3390673	0.02884233
	TN124_ERR037321	10	0.3270449	0.221603
	TN126_ERR037322	6	0.4294605	0.4224717
	TN128_ERR037323	3	0.09750629	0.08733423
	TN129_ERR037324	11	0.3676589	0.06198691
	TN12_ERR037302	10	0.3727368	0.3524048
	TN138_ERR037325	10	0.437225	0.427548
	TN149_ERR037326	8	0.3868991	0.3784844
	TN161_ERR037327	2	0.08760107	0.08725725

	TN163_ERR037328	2	0.07761408	0.07131885
	TN170_ERR037329	17	0.6713735	0.6099389
	TN171_ERR037332	7	0.3849667	0.3469346
	TN172_ERR037333	8	0.4116688	0
	TN176_ERR037330	18	0.6970785	0.4851102
	TN177_ERR037331	8	0.2876884	0.2813476
	TN24_ERR037299	6	0.8180671	0.7046093
	TN28_ERR037295	9	0.2901523	0.2559663
	TN30_ERR037305	8	0.3072847	0
	TN31_ERR037303	5	0.1909596	0.1728963
	TN38_ERR037304	10	0.5030703	0.2507862
	TN44_ERR037306	5	0.4790666	0.4366026
	TN48_ERR037307	6	0.2353474	0.207091
	TN51_ERR037308	16	0.7247479	0.5217364
	TN57_ERR037309	8	0.2702777	0.2658199
	TN72_ERR037312	7	0.2805417	0.2497752
	TN76_ERR037311	11	0.4002301	0.3884019
	TN77_ERR037310	9	0.3143319	0.2938348
	TN85_ERR037315	17	1.114377	0.9160358
	TN88_ERR037313	6	0.7216628	0.7091752
	TN90_ERR037316	13	0.4896906	0.3805109
	Tapidor_ERR037334	10	0.4500867	0.3721501

Tapidor, Ningyou-7 and the Tapidor x Ningyou-7 population

FPKM Confidence high	TPM
0.271828	0.1581286
0.6124845	0.3855928
0	0
0	0
0.2871137	0.1669071
0.3935434	0.313914
0.8264428	0.6443172
0	0
0	0
0	0
2.561089	2.021563
3.41E-20	5.52E-37
0	0
0	0
0	0
0	0
0	0
0	0
0	0
0	0
0	0
0	0
0	0
0	0
0	0
0	0
0	0
0	0
0	0
0	0
0	0

0	0
0	0
0	0
0	0
0	0
0	0
0	0
0	0
0	0
0	0
0	0
0	0
0	0
0	0
0	0
0	0
0	0
0	0
0	0
4.633826	2.365997
9.408311	5.497614
5.524546	2.816585
4.861502	2.531896
3.91257	2.235188
5.458762	3.966943
5.236073	3.876276
5.686298	3.880153
5.967703	4.078935
6.787098	4.600241
5.503531	4.285506
0.0404371	0.03017504
7.05E-16	2.02E-27
0.2041466	0.1392246

2.92E-17	4.36E-29
0.095068	0.06513085
1.29E-14	2.97E-28
0.03875444	0.02517793
5.89E-10	1.51E-17
1.38E-13	3.06E-23
0.03294048	0.02248897
0.08779041	0.05131935
2.21E-16	7.34E-29
1.21E-10	1.05E-18
0.03831809	0.02575713
8.42E-16	1.05E-28
0.2377126	0.066954
0.2797166	0.03532725
0.1206086	0.05172356
0.06455451	0.04329789
6.90E-11	1.63E-18
0.0627381	0.04467514
7.91E-19	2.65E-33
9.98E-17	1.81E-29
0.05344719	0.03056242
2.82E-15	3.38E-26
9.10E-09	8.31E-14
7.41E-14	9.19E-26
0.05757544	0.02470954
1.62E-13	3.73E-25
0.03856848	0.02769259
0.05522242	0.03214328
0.04613227	0.02328608
6.84E-10	1.41E-16
0.03793936	0.02381222
3.37E-15	8.31E-29
7.55E-17	1.55E-26

2.42E-15	7.28E-29
0.07998287	0.04644913
2.03E-14	4.51E-26
0	0
0	0
0	0
0	0
0	0
0	0
7.19E-26	5.01E-44
0	0
0	0
0	0
0	0
1.74E-15	6.35E-29
0	0
0	0
0	0
0	0
0	0
0	0
0	0
0	0
0	0
0	0
0	0
7.40E-17	5.73E-33
0	0
0	0
0	0
0	0
0	0
0	0

0	0
0	0
0	0
0	0
0	0
0	0
0	0
0	0
0	0
0	0
0	0
0	0
0	0
0	0
0	0
0	0
0	0
1.73E-22	3.00E-39
0	0
3.24E-21	4.99E-41
0.1867761	0.03621731
4.10E-19	1.44E-34
2.91E-19	4.45E-38
0	0
0	0
0	0
0	0
0	0
0	0
0	0
0.1604257	0.04586418
0	0
0	0

0	0
0	0
0	0
0	0
0	0
0	0
0	0
0	0
0	0
0	0
0	0
0	0
0	0
0	0
0	0
0	0
0	0
0	0
0	0
0	0
6.48E-19	8.65E-36
1.40E-18	2.24E-31
2.72E-17	2.80E-37
6.59E-32	2.73E-64
0	0
0	0
0	0
0	0
0	0
0	0
2.43E-19	1.30E-41
0	0
0	0

3.26E-19	1.77E-29
0	0
8.68E-28	1.39E-41
0	0
0.1210097	0.06985363
0.08222614	0.05176053
1.98E-20	2.32E-37
0	0
0.04206064	0.02445101
0.02813636	0.02244326
0.0421691	0.03287617
0	0
0	0
0	0
0.03984651	0.03145234
3.31E-24	1.83E-45
0	0
0	0
0	0
0	0
0	0
1.72E-17	5.91E-33
0	0
0	0
0	0
0	0
0	0
1.47E-22	1.40E-44
0	0
0	0
0	0
0	0
0	0

0	0
0	0
0	0
9.20E-14	4.65E-27
0	0
0	0
0	0
0	0
0	0
0	0
0	0
0	0
0	0
0	0
0	0
0	0
0	0
0	0
0	0
0	0
0	0
0	0
0	0
0	0
0	0
0	0
0	0
0	0
0	0
0	0
0	0
0	0
0	0

0	0
0	0
0	0
0	0
0	0
0	0
0	0
0	0
0	0
0	0
0	0
0	0
0	0
0	0
0	0
0	0
0	0
0	0
0	0
0	0
0	0
0	0
0	0
0	0
0	0
0	0
0	0
0	0
0	0
0	0
0	0
0	0
0	0

0	0
0	0
0	0
0	0
0	0
7.35772	1.90737
3.744512	1.838734
11.58224	4.130881
62.9997	29.73521
11.60892	3.913489
0.794283	0.58169
2.490961	1.338925
45.81246	29.65461
54.02361	35.15582
35.38247	22.27805
1.783346	0.3544986
0.1825887	0.1364018
3.06E-13	2.81E-25
1.44E-09	8.26E-18
1.09E-13	2.89E-26
4.96E-11	2.79E-22
4.26E-13	1.70E-25
4.55E-14	1.14E-26
0.3238818	0.1080703
5.19E-14	1.28E-26
8.99E-13	2.88E-24
1.59E-12	1.40E-23
2.15E-10	1.54E-19
7.56E-12	3.33E-22
1.54E-10	5.35E-19
1.52E-13	1.13E-25
7.46E-14	1.84E-25
8.07E-14	4.41E-26

1.03E-09	1.24E-18
1.00E-12	3.47E-24
1.90E-11	8.04E-21
0.142038	0.09895664
1.519154	1.045057
7.07E-07	1.16E-11
3.55E-12	9.65E-23
3.74E-06	2.20E-11
1.93E-13	9.74E-26
0.3783497	0.2360451
8.01E-11	2.12E-20
6.53E-10	2.48E-18
2.94E-12	7.00E-24
1.24E-09	6.99E-18
4.84E-12	1.87E-22
3.93E-12	1.87E-23
8.56E-11	4.96E-20
1.47E-13	2.99E-25
3.11E-13	2.35E-25
5.01E-12	6.71E-23
1.62E-11	7.29E-23
0.195573	0.1258566
0	0
0	0
0	0
0	0
0	0
0	0
0	0
0	0
0	0
0	0
0	0

0	0
0	0
0	0
0	0
0	0
0	0
0	0
0	0
0	0
0	0
0	0
0	0
0	0
0	0
0	0
0	0
0	0
0	0
0	0
0	0
0	0
0	0
0	0
0	0
0	0
0	0
0	0
0	0
0	0
0	0
0	0
0	0
0	0

0	0
0	0
0	0
0	0
0	0
0	0
3.13E-20	1.79E-39
3.35E-17	1.49E-32
2.18E-22	2.13E-38
0.02064234	0.003976698
0.691058	0.156209
0	0
0	0
0	0
0.008307326	0.001998395
0	0
1.21E-27	9.32E-49
1.13E-05	1.40E-06
3.32E-23	2.16E-43
3.91E-19	2.04E-35
0	0
8.33E-21	1.81E-32
9.49E-16	2.13E-28
0	0
5.02E-23	9.35E-38
2.76E-17	4.13E-33
1.14E-06	2.23E-09
1.18E-17	6.32E-34
0	0
6.53E-18	1.70E-25
1.47E-18	1.33E-31
4.16E-17	4.18E-32
1.01E-16	3.33E-27

1.81E-16	1.40E-31
1.13E-17	1.63E-27
1.35E-06	8.68E-10
2.57E-18	3.41E-35
3.85E-21	4.86E-40
2.10E-25	1.14E-45
6.69E-05	4.57E-06
2.03E-15	1.29E-25
1.25E-17	1.96E-33
6.07E-19	2.58E-31
2.13E-17	2.93E-31
1.36E-18	1.90E-35
0.009579351	0.001283698
4.11E-16	1.29E-29
1.50E-14	9.29E-28
5.07E-19	9.32E-33
3.92E-18	3.97E-32
1.75E-18	5.04E-33
4.67E-26	9.85E-42
0	0
1.52E-19	5.58E-33
0	0
1.075181	0.001197652
6.221382	3.605799
17.68716	10.93903
6.547087	3.408064
5.192892	3.000608
2.526188	1.467668
4.784488	3.775069
5.759209	4.490028
2.365455	1.709128
2.632846	1.900058
2.740802	1.972353

4.539665	3.583327
1.15E-16	2.57E-31
6.06E-17	2.18E-32
1.51E-14	3.81E-28
0	0
1.76E-13	8.60E-26
3.39E-18	1.70E-28
3.36E-17	1.61E-32
0.1400114	0.09891473
2.67E-16	3.03E-31
4.75E-18	1.26E-32
8.05E-15	1.12E-27
1.21E-21	3.38E-33
1.11E-14	2.04E-28
3.35E-17	1.06E-32
6.39E-17	1.08E-31
1.42E-16	1.17E-25
6.37E-19	1.52E-32
1.30E-18	4.70E-26
2.99E-17	3.36E-33
5.70E-15	8.38E-28
4.33E-15	5.72E-27
2.94E-17	2.38E-30
1.34E-20	1.13E-32
2.09E-13	6.01E-23
1.15E-17	4.41E-32
8.78E-15	1.03E-27
2.87E-17	6.57E-33
1.53E-17	1.68E-27
2.16E-18	1.62E-30
0	0
2.92E-16	6.60E-31
1.69E-18	1.70E-33

3.09E-14	9.96E-27
3.94E-15	2.76E-27
1.31E-15	0
9.50E-20	1.90E-32
2.66E-16	2.38E-30
1.33E-14	9.73E-28
5.28E-16	1.95E-28
4.991039	1.969666
5.052195	2.071534
6.02997	2.36998
7.062854	3.326982
8.877743	3.821746
2.555459	1.787426
3.106676	2.001085
3.769594	2.199118
3.880482	2.517789
2.953572	1.604999
4.392504	2.59639
5.935315	2.748858
1.024793	0.358823
2.522718	0.9125014
2.151388	1.09149
4.097166	1.427844
3.864414	1.917368
2.480218	1.147978
1.003448	0.2770011
1.760451	0.642606
1.527641	0.6791017
1.581907	0.6342608
2.56E-08	7.58E-16
3.740284	1.33209
0.8077762	0.5117289
2.05167	0.7160956

3.502767	0.7627759
5.863858	0.7521246
1.544452	0.4540025
4.741584	2.079596
9.83E-10	6.10E-18
5.009875	2.400825
2.528451	1.099024
0.8005128	0.400797
1.08E-12	2.97E-24
4.383734	1.821155
1.441136	0.5331669
4.159735	1.777944
2.146979	1.10876
6.303878	2.175857
2.111031	1.134319
1.356326	0.724047
0.8301472	0.4359988
0.9200027	0.2919278
1.115456	0.4240309
3.41E-10	2.62E-19
0.9958073	0.2888964
1.24E-07	4.81E-14
1.113341	0.2814561
0.2464683	0.1022832
2.10E-16	4.64E-27
5.34E-12	1.94E-20
3.03E-19	1.72E-32
8.59E-13	7.47E-25
1.88E-14	2.08E-26
0	0
0	0
5.21E-23	1.17E-41
9.47E-17	1.32E-32

5.23E-22	1.00E-41
3.46E-19	8.00E-32
0	0
1.64E-10	6.92E-21
0	0
0	0
0	0
0	0
0.0243263	0.005461134
0.001110462	5.43E-07
0.8349221	0.3018913
0	0
1.16E-12	1.31E-19
1.03E-13	1.29E-26
0	0
0.0001527237	5.69E-06
0	0
0	0
0	0
0	0
0	0
8.29E-18	1.83E-19
0	0
0	0
2.69E-14	6.19E-26
2.29E-14	2.73E-25
1.44E-05	2.66E-06
0	0
1.66E-27	1.93E-56
0.3755089	0.08490488
1.86E-10	6.20E-21
3.02E-11	1.60E-22
0.005004739	0.001210914

1.36E-14	2.12E-24
2.62E-12	1.45E-24
8.03E-15	6.15E-28
1.30E-15	4.08E-26
2.90E-06	1.11E-12
0	0
4.49E-13	5.64E-22
6.98E-15	2.81E-28
1.37E-06	7.61E-14
6.40E-14	6.77E-21
0.0004700123	1.46E-08
5.09E-05	1.19E-10
4.93E-08	1.65E-16
5.84E-14	1.40E-26
1.11E-12	3.80E-25
5.61E-06	6.13E-12
4.574	1.361247
1.84E-08	5.83E-17
0.147831	0.004159981
4.13E-14	8.15E-24
1.87E-27	1.66E-47
2.31E-21	2.04E-38
1.45E-24	1.14E-46
7.07E-27	1.30E-52
8.59E-20	1.78E-31
2.67E-25	8.07E-45
2.12E-17	5.36E-25
1.56E-25	1.11E-44
9.71E-28	1.29E-54
9.10E-15	2.66E-24
1.24E-16	2.73E-33
1.43E-24	1.08E-40
2.36E-23	3.96E-45

1.40E-29	7.92E-62
0	0
2.64E-21	9.95E-34
2.02E-19	1.37E-38
3.00E-20	1.34E-38
9.04E-32	1.68E-66
5.58E-15	6.51E-30
7.82E-16	1.05E-30
4.77E-14	7.28E-29
1.28E-20	1.89E-38
5.02E-09	5.70E-17
1.43E-23	5.58E-45
7.55E-25	6.57E-37
8.19E-22	1.84E-44
4.38E-12	1.86E-22
1.23E-25	5.24E-50
6.59E-19	6.65E-37
4.73E-31	4.32E-65
4.88E-17	5.92E-31
6.07E-38	1.15E-78
7.05E-29	1.21E-60
1.44E-26	1.38E-47
1.03E-21	6.47E-37
6.16E-37	9.36E-77
2.09E-16	8.12E-23
24.97749	12.19847
34.92853	18.88802
21.81151	10.43656
19.29249	9.115542
23.32074	10.83667
25.20461	18.51945
29.98355	21.30329
15.28997	9.891837

13.90977	8.940801
12.93365	8.092618
28.39911	20.51949
29.7479	17.37138
10.74617	5.225639
22.87542	12.01907
18.28576	8.756802
24.48138	12.35911
25.51013	13.33157
30.98703	16.80804
18.80106	9.484045
26.77935	14.12338
14.10712	7.112524
18.21073	9.697528
6.85E-10	3.05E-18
37.19798	21.02943
15.11944	7.41927
29.29061	14.85595
12.70877	5.632732
10.31869	4.229802
17.93355	8.72163
27.28081	15.54573
2.57E-12	1.02E-22
25.43245	14.39216
21.80732	12.08257
8.701843	4.08977
6.01E-09	4.90E-16
27.27808	14.89191
21.97555	11.42857
20.04252	11.1676
25.9841	13.44185
6.068639	3.032336
18.67077	9.822935

26.04115	13.34114
9.294552	4.14664
13.97056	6.570788
9.422897	4.510472
1.49E-10	1.39E-19
16.62009	7.80775
3.57E-12	8.49E-22
12.67616	5.988152
3.82E-09	1.59E-15
0.3234826	0.08242444
0.09414451	0.05926916
0	0
2.38E-06	1.80E-09
1.671551	0.7064919
0	0
0.03302563	0.02574767
0	0
0	0
0	0
0.03198871	0.02524989
2.49E-23	1.82E-44
0	0
0	0
0	0
0	0
0	0
0	0
9.31E-19	1.68E-31
0	0
0	0
0	0
1.40E-17	5.12E-33
1.45E-12	7.66E-24

7.64E-23	7.51E-43
3.62E-22	4.72E-44
0	0
0	0
0	0
0	0
0	0
2.84E-24	1.96E-43
1.81E-22	2.45E-42
0	0
4.52E-21	4.91E-41
3.05E-28	7.37E-55
8.17E-23	2.78E-45
0	0
6.08E-20	9.25E-36
0	0
0	0
1.04E-22	3.66E-40
0	0
0	0
1.18E-18	1.11E-33
0	0
3.69E-27	2.06E-51
1.74E-23	1.54E-43
0	0
0	0
1.401177	0.5199271
1.732075	1.044809
3.967733	1.747849
2.867684	1.583564
1.849557	1.059886
0.6569947	0.5240586
0.585431	0.4561291

1.354144	0.9387103
1.289213	0.9303446
1.144822	0.7649245
0.8919591	0.7040568
2.713478	1.743143
0.6825702	0.4613136
2.369022	1.51969
0.361505	0.2344259
1.318829	0.5784365
1.734427	0.9004769
3.61466	1.729368
0.9781265	0.642314
2.625842	1.139021
0.3894716	0.2843684
2.029283	1.246351
7.30E-17	4.86E-30
1.665869	1.036138
2.232482	1.01284
3.366862	1.123789
1.054906	0.6907011
0.2213048	0.1481528
0.9443633	0.5671095
1.966776	1.213197
7.69E-10	2.16E-17
1.260494	0.6798762
1.444259	0.9230138
0.4418632	0.2958908
5.56E-14	1.05E-27
1.348899	0.9363373
2.064056	1.146853
2.083083	0.6834073
1.910626	1.258249
0.6486334	0.4652396

1.983689	0.7702905
1.553759	0.9737753
0.4501817	0.3248926
1.869323	0.7114948
0.6992366	0.4803315
3.78E-17	2.27E-28
2.403243	1.075847
3.96E-15	8.93E-27
2.142337	0.5532153
1.17E-15	1.05E-27
2.406245	1.333801
4.88282	3.007688
6.144368	2.989927
1.805636	0.68503
6.83444	3.626287
2.968271	2.367672
2.040334	1.589989
0.4391703	0.3173029
0.6986326	0.5041853
0.6756692	0.4862293
2.686542	2.106647
0.9724051	0.5631246
3.98E-14	3.33E-24
3.37E-16	5.70E-29
3.72E-18	4.83E-32
4.77E-10	4.09E-17
4.28E-14	4.84E-27
8.69E-16	4.79E-31
1.12E-16	3.72E-30
4.58E-17	2.72E-31
4.00E-15	1.33E-26
9.39E-13	3.36E-24
1.69E-14	1.92E-27

1.19E-08	7.49E-13
9.25E-14	4.58E-27
9.72E-09	1.77E-16
1.61E-17	4.92E-32
1.455018	0.526602
0.2036059	0.1080929
2.63E-12	1.03E-20
1.55E-16	1.25E-30
1.38E-14	2.13E-27
1.76E-15	8.58E-30
1.11E-14	4.02E-28
2.12E-14	6.60E-25
1.45E-14	2.03E-26
3.67E-14	3.15E-27
0.4738073	0.3416664
0.08491427	0.05736764
3.08E-15	1.02E-27
7.20E-14	2.69E-26
0.5125251	0.3709769
1.71747	0.9491224
0.04273242	0.02930015
7.11E-17	9.73E-31
1.13372	0.7281303
2.52E-16	8.10E-31
1.51E-05	1.03E-05
1.17E-11	3.59E-18
1.62E-15	4.79E-26
1.663273	0.7785622
0.9641807	0.4948282
5.421754	2.649903
11.02096	5.832047
8.527116	4.432761
1.16746	0.7491101

1.76579	1.171914
7.408129	5.016057
7.211764	4.889009
6.257456	4.167668
1.404799	0.9848209
5.761144	3.316722
1.671267	0.9309682
2.074762	0.9014509
3.09819	1.492413
2.230899	1.072947
4.122175	2.066488
1.899017	1.016325
3.845724	2.105606
2.463111	1.239636
2.97444	1.617463
2.863124	1.504748
0.514359	0.2495465
2.49997	1.242726
1.621688	0.7119464
2.70521	1.119958
3.098764	1.606563
1.855516	0.7774228
3.961669	2.185683
5.325841	3.032342
0.843943	0.5782434
6.960842	4.167233
4.96746	2.918971
3.110192	1.592043
1.043174	0.5773333
2.210337	1.15552
1.3707	0.7603033
3.444946	2.035243
1.460919	0.9729286

2.200052	1.037981
2.404565	1.283894
4.560183	2.551083
2.940699	1.599294
2.37718	1.177464
1.027365	0.4666514
0.4867808	0.2891361
2.232852	1.012878
0.6996159	0.367593
2.451515	1.282689
0.7739275	0.4245395
0	0
0	0
0	0
0	0
0	0
0	0
0	0
0	0
0	0
0	0
4.64E-06	2.99E-10
0	0
0	0
0	0
0	0
0.1368345	0.09374499
0	0
0	0
0	0
0	0
0	0
0	0

0	0
0	0
0	0
0	0
0	0
0	0
0	0
0	0
0	0
0.0924063	0.06619291
0	0
0	0
0	0
0	0
0	0
0	0
0	0
0	0
0	0
0	0
0	0
0	0
0	0
0	0
0	0
0	0
0	0
0	0
0.02016179	0.01171931
0.0278033	0.01750371
0	0
0	0
2.919292	1.575302

0.009116795	0.007272106
0.01863739	0.01453022
0	0
0	0
0	0
0.00978798	0.00772602
0	0
0	0
0	0
0	0
0	0
0	0
0	0
0	0
0	0
0	0
0	0
0	0
0	0
0	0
0.04381159	0.00935578
0	0
0	0
0	0
0	0
0	0
0	0
0	0
0	0
0	0
0	0
0	0
0	0

0	0
0	0
0	0
0	0
0	0
0	0
0.0006554323	2.49E-06
0	0
0	0
0	0
0	0
0	0
0	0
0	0
0	0
0	0
0	0
0	0
0	0
0	0
0	0
0	0
0	0
0	0
0	0
0	0
0	0
0	0
0	0
0	0
0	0
0	0
0	0

0	0
0	0
0	0
0	0
0	0
0	0
0	0
0	0
0	0
0	0
0	0
0	0
0	0
0	0
0	0
0	0
0	0
0	0
0	0
0	0
0	0
0	0
0	0
0	0
0	0
0	0
0	0
0	0
0	0
3.13E-20	1.79E-39
3.35E-17	1.49E-32
2.18E-22	2.13E-38
0.02064234	0.003976698

0.691058	0.156209
0	0
0	0
0	0
0.008307326	0.001998395
0	0
1.21E-27	9.32E-49
1.13E-05	1.40E-06
3.32E-23	2.16E-43
3.91E-19	2.04E-35
0	0
8.33E-21	1.81E-32
9.49E-16	2.13E-28
0	0
5.02E-23	9.35E-38
2.76E-17	4.13E-33
1.14E-06	2.23E-09
1.18E-17	6.32E-34
0	0
6.53E-18	1.70E-25
1.47E-18	1.33E-31
4.16E-17	4.18E-32
1.01E-16	3.33E-27
1.81E-16	1.40E-31
1.13E-17	1.63E-27
1.35E-06	8.68E-10
2.57E-18	3.41E-35
3.85E-21	4.86E-40
2.10E-25	1.14E-45
6.69E-05	4.57E-06
2.03E-15	1.29E-25
1.25E-17	1.96E-33
6.07E-19	2.58E-31

2.13E-17	2.93E-31
1.36E-18	1.90E-35
0.009579351	0.001283698
4.11E-16	1.29E-29
1.50E-14	9.29E-28
5.07E-19	9.32E-33
3.92E-18	3.97E-32
1.75E-18	5.04E-33
4.67E-26	9.85E-42
0	0
1.52E-19	5.58E-33
0	0
1.075181	0.001197652
30.18076	16.71474
21.65118	12.85841
36.03756	19.3326
10.598	5.435603
20.35354	10.74843
27.32371	21.30387
35.28668	27.28104
8.0347	5.784885
8.746447	6.067077
8.054512	5.516817
32.80699	25.74641
1.13E-14	3.00E-27
3.26E-11	1.37E-18
9.77E-15	5.04E-26
3.19E-15	5.79E-29
2.49E-13	1.40E-23
1.13E-13	2.99E-26
8.27E-15	3.23E-26
0.338444	0.2273744
4.10E-15	7.24E-29

1.55E-15	1.66E-29
1.11E-16	3.69E-29
2.66E-11	6.40E-19
1.35E-15	2.06E-29
3.42E-15	1.53E-27
1.34E-14	1.22E-24
1.17E-14	2.22E-28
3.06E-15	3.63E-28
2.37E-15	1.06E-28
2.49E-15	2.56E-28
1.52E-11	6.39E-19
1.94E-15	4.93E-29
1.32E-15	1.78E-29
1.20E-13	1.31E-22
3.23E-14	3.28E-26
1.79E-09	1.19E-17
4.17E-15	1.58E-28
1.68E-15	1.05E-28
5.54E-15	1.01E-28
1.51E-11	1.67E-19
2.13E-15	3.99E-28
1.07E-12	1.90E-21
1.78E-15	2.12E-29
4.07E-15	1.85E-28
1.04E-15	2.37E-29
0.04817373	0.03232619
2.38E-13	7.13E-23
6.35E-14	3.31E-24
2.69E-14	1.41E-27
4.62E-11	5.46E-18
3.186099	1.540759
3.34329	1.83841
1.03353	0.5010506

3.071066	1.519595
1.623223	0.7624236
3.003078	2.222989
2.266626	1.738749
1.436621	0.9362871
2.010351	1.395239
0.8539927	0.6075407
2.532856	1.981269
0.8474716	0.3264929
0.147673	0.075824
0.6288506	0.2379616
0.5150928	0.3534681
0.2465364	0.1568914
0.3992433	0.1541759
0.2815234	0.1897277
0.271781	0.1875705
0.2496655	0.1723702
0.5779899	0.2118696
0.07563627	0.05394786
1.40E-16	2.51E-31
0.4818954	0.3302936
0.2678238	0.187169
0.3124139	0.1142415
0.1731312	0.1183338
0.05623876	0.03833495
0.2280675	0.06453182
0.4540384	0.2185459
4.89E-16	9.64E-30
0.3261497	0.2254491
0.5294599	0.2528912
0.2523099	0.1040061
1.79E-06	3.45E-08
0.3997033	0.2766225

0.2138383	0.08828552
0.5717313	0.411967
0.4291894	0.1659008
0.1101396	0.07858884
0.1850465	0.1328683
0.1066693	0.07749489
0.1224038	0.05817576
0.1449708	0.09979799
0.09163243	0.05960033
1.34E-08	3.14E-13
0.07362589	0.04707796
0.119321	0.02913791
0.1753844	0.1190015
0.02219195	0.01764576
0.3130615	0.1308901
0.5339533	0.2867757
0.2459427	0.1374396
6.849665	3.599205
3.936597	2.12836
0.2550412	0.201849
0.3321434	0.2551047
6.840261	4.803956
6.547954	4.59441
6.637915	4.675038
0.2181625	0.171794
5.955989	4.039508
4.976842	3.080853
7.173643	4.475512
6.906089	4.217004
5.578146	3.352156
7.672379	4.84439
6.88E-13	2.39E-23
7.767016	4.871451

1.32E-13	1.39E-24
8.14E-15	4.78E-27
1.05E-13	6.59E-25
6.732224	4.232994
6.82241	4.201955
1.49E-14	7.06E-27
6.08E-14	2.24E-25
1.24E-13	3.34E-24
4.395931	2.775347
8.713248	5.375534
0.03917542	0.02134181
5.140258	3.251437
9.33E-15	2.14E-27
8.237133	5.128727
0.03484296	0.02291507
1.25E-13	1.63E-25
7.450648	4.840059
5.962069	3.783657
8.56174	5.626074
0.1331272	0.03349218
6.49643	3.947484
3.01E-14	6.75E-26
2.77E-14	1.46E-25
6.83688	4.401136
6.744623	4.107827
7.320832	4.273425
3.705654	2.136094
8.537891	5.075499
5.027198	2.993134
9.847574	5.988745
3.52E-13	1.55E-23
1.235115	0.7184949
0.7872366	0.4956089

1.032746	0.5788561
0.3422698	0.1977898
0.09876414	0.05741432
0.2951762	0.2354504
0.3295639	0.2569369
0.2273112	0.1642406
0.09757736	0.07041908
0.2323525	0.167207
0.1977747	0.156111
0.1243369	0.09174926
0	0
2.64E-27	3.46E-54
6.27E-25	5.57E-38
0	0
0	0
0	0
7.83E-24	2.94E-47
3.55E-21	8.83E-38
0	0
5.94E-20	2.52E-35
3.51E-22	2.02E-36
1.06E-18	1.52E-29
0	0
0	0
3.11E-22	6.34E-43
0	0
0	0
2.98E-14	2.48E-28
1.14E-29	3.32E-61
4.97E-19	3.85E-26
3.72E-08	2.60E-08
1.14E-20	3.93E-38
4.27E-21	2.85E-37

1.59E-23	2.07E-40
3.40E-51	2.74E-104
3.52E-20	2.11E-38
4.03E-23	1.04E-37
1.40E-43	6.44E-89
3.25E-37	1.76E-76
5.25E-47	1.37E-95
0	0
0	0
0	0
0	0
0	0
1.52E-12	5.20E-25
8.20E-24	4.87E-35
6.45E-20	8.35E-39
8.993329	4.72748
7.130019	3.881496
7.867748	3.881634
4.943122	2.402252
8.378265	4.348785
9.713163	7.375392
9.257265	6.93672
2.787677	1.997397
3.3375	2.325199
2.253008	1.529161
10.8016	8.20695
0.5905543	0.2121019
1.69E-09	1.01E-17
0.1071132	0.06591276
0.04932022	0.02252143
0.1542817	0.08661411
1.07E-08	3.19E-16
0.5552904	0.2313266

0.1022585	0.02034461
1.81E-10	1.66E-19
0.1653609	0.04937525
0.06573148	0.04022821
0.2176771	0.06932808
0.1073594	0.05878375
2.81E-09	3.06E-17
1.24E-10	3.44E-20
6.31E-09	1.84E-16
2.60E-12	2.65E-23
1.15E-09	3.92E-18
2.08E-12	2.05E-23
0.04780698	0.02196873
0.2952008	0.1028138
1.20E-10	5.98E-20
0.2888884	0.106012
0.5232891	0.213899
0.0429036	0.01962736
0.103175	0.06461893
0.140827	0.07051403
0.4601429	0.2412719
0.3102565	0.0960761
0.03766379	0.02399227
0.09627595	0.05613528
9.14E-13	2.99E-24
0.07821558	0.04924308
0.2380431	0.06611939
3.68E-12	5.55E-23
1.70E-12	9.25E-24
1.15E-09	7.13E-18
0.09928752	0.02188726
0.7481005	0.2506136
0	0

0	0
0	0
0	0
0	0
0	0
0	0
0	0
0	0
0	0
0.09500131	0.0749881
0	0
0	0
0	0
0	0
0	0
0	0
0	0
0	0
0	0
0	0
0	0
0	0
0	0
0	0
0	0
0	0
0	0
0	0
0	0
0	0
0	0
0	0
0	0

0	0
0	0
0	0
0	0
0	0
0	0
0	0
0	0
0	0
0	0
0	0
0	0
0	0
0	0
0	0
0	0
0	0
0	0
0	0
0	0
0	0
0	0
0	0
0	0
0	0
0	0
0	0
2.82E-20	3.55E-40
0	0
0	0
0	0
0	0
0	0

0	0
0	0
0	0
0	0
0	0
0	0
0	0
0	0
0	0
0	0
0	0
0	0
0	0
0	0
0	0
0	0
0	0
0	0
0	0
0	0
0	0
0	0
0	0
0	0
0	0
0	0
0	0
0	0
0	0
0	0
0	0
0	0
0	0

27.68174	16.1031
38.36585	24.15342
798.3912	20.77573
24.08864	13.92186
20.73745	12.05525
22.6368	18.05648
14.91014	11.62435
9.887364	7.143981
11.69283	8.438413
11.98611	8.625517
16.39936	12.94463
15.12175	11.28888
7.274361	4.935451
0	0
0	0
21.95681	13.46484
0	0
0	0
0	0
0	0
17.53419	11.94149
11.29158	7.967485
11.07578	7.2328
0	0
0	0
0	0
0	0
9.61158	6.676306
8.768442	6.101932
0	0
0	0
0	0
19.20835	10.91178

21.27386	8.802103
0	0
0	0
0	0
0	0
2.76E-22	9.26E-36
0	0
0	0
11.4287	7.078548
11.59059	8.36673
0	0
0	0
0.5588834	0.3750828
10.27386	6.812713
0	0
0	0
0	0
1.788112	1.040186
4.67085	2.940558
0.03273471	0.01840531
0.2494773	0.1441836
0.2431672	0.1413598
1.754557	1.399541
1.235919	0.9635557
0.04440506	0.03208428
0.05931307	0.04280473
0	0
1.144247	0.9031967
0	0
0	0
0	0
0.1034497	0.07110706
0.07084282	0.04853425

0	0
0	0
0.08949739	0.06322779
0	0
0.04851165	0.03544472
0.04972901	0.03546941
0	0
0.0489866	0.03362829
0	0
0.06604351	0.04305617
0.07066862	0.04830144
0	0
0.1176629	0.08188123
0	0
0.04548245	0.03368111
0	0
0	0
0	0
0.1328223	0.09280864
0	0
0	0
0	0
0	0
0.07023049	0.05037362
0	0
0	0
0	0
0	0
0	0
0	0
0.05930163	0.03945192
0	0
0.05277146	0.03581574

0	0
5.772974	3.358272
14.18997	8.93335
0.3132219	0.1761111
0.08823061	0.05099227
2.461899	1.43117
17.20211	13.72144
6.188707	4.824884
0.0490202	0.03541888
0.01640374	0.01183816
0.03261088	0.02346765
15.385	12.14396
0.06558493	0.04902298
4.65E-23	3.57E-45
0	0
0	0
1.82E-15	4.50E-30
4.99E-17	6.78E-31
0	0
6.43E-20	1.51E-35
8.17E-22	4.30E-42
9.46E-31	7.24E-63
1.18E-16	8.09E-30
1.07E-16	2.07E-32
8.84E-17	4.94E-32
1.72E-15	1.64E-29
4.88E-17	7.66E-29
0	0
0	0
0.2582177	0.1796929
1.30E-18	2.22E-32
8.31E-15	3.66E-28
5.22E-33	4.93E-66

2.65E-17	2.25E-32
1.81E-24	1.85E-43
4.92E-16	8.40E-27
6.72E-18	1.52E-35
4.87E-05	1.55E-06
6.65E-10	8.24E-13
0	0
1.66E-22	6.06E-41
0	0
0.06747015	0.04903658
0.05319118	0.03839172
0	0
4.17E-17	1.74E-31
0.1673037	0.112277
3.16E-16	3.96E-31
9.54E-16	1.14E-26
1.12E-27	6.16E-54
6.42E-19	2.64E-35
0	0
0	0
0	0
0	0
0	0
0	0
0	0
0	0
0	0
0	0
0	0
0	0
0	0
0	0
0	0

0	0
0	0
0	0
0	0
0	0
0	0
0	0
0	0
0	0
0	0
0	0
0	0
0	0
0	0
0	0
0	0
0	0
0	0
0	0
0	0
0	0
0	0
0	0
0	0
0	0
0	0
0	0
0	0
0	0
0	0
0	0
0	0
0	0

0	0
0	0
0	0
0	0
0	0
0	0
0.6777737	0.3940085
0	0
0	0
0	0
0	0
0	0
0	0
0	0
0	0
0	0
0	0
0	0
0.07039784	0.04868232
0	0
0	0
0	0
0	0
0	0
0	0
0	0
0	0
0	0
0	0
0	0
0	0
0	0
0	0

0	0
0	0
0	0
0	0
0	0
0	0
0	0
0	0
0	0
0	0
0	0
0	0
0	0
0	0
0	0
0	0
0	0
0	0
0	0
0.1011155	0.0588212
4.42E-13	1.05E-23
3.67E-17	1.82E-32
0.3697159	0.2130278
2.788433	1.152811
0.1413371	0.09966214
0.02788435	0.02173939
0.2343409	0.1655894
0.1112495	0.08026078
0.3752266	0.2413802
0.09976651	0.07874946
0.6673384	0.396816
2.36E-12	3.52E-24
4.83E-17	8.26E-30

0	0
0.759581	0.4992333
7.73E-13	8.09E-25
0.3307676	0.222902
1.02E-12	2.54E-24
0.2647849	0.1828107
1.91E-18	8.22E-36
1.014718	0.6540212
0.8134261	0.5627221
0.417219	0.2516303
0.3348823	0.2342214
0.3138117	0.2045426
1.71E-14	1.44E-28
2.126218	0.6227856
1.638971	1.014181
1.01E-10	3.43E-12
0	0
1.61E-14	1.74E-25
0.08546523	0.05968888
5.76E-15	2.70E-29
5.66E-11	4.67E-19
0.3599847	0.25045
2.17E-13	8.77E-23
1.49E-13	1.41E-26
0	0
4.62E-05	7.55E-07
0.5682599	0.2569161
1.138256	0.7383142
1.919358	0.7597713
1.143469	0.737225
0.3038398	0.2094138
1.48E-05	9.76E-06
1.167628	0.7718423

3.21E-14	4.91E-23
1.104832	0.4450315
1.08E-15	2.89E-29
1.183628	0.3075042
0.3962841	0.2411181
7.735568	3.317826
9.378077	3.624736
24.24779	10.81926
0.4817944	0.3840019
1.785803	1.076946
3.479879	2.226754
4.565981	2.713037
1.782906	1.17532
0.355679	0.2407553
2.82E-06	8.51E-10
2.30E-07	1.82E-09
3.39E-06	8.22E-09
5.98E-06	1.59E-09
0.08103396	0.05551618
0	0
0	0
0.05015689	0.03543441
2.20E-06	1.62E-12
5.80E-05	3.79E-09
2.58E-14	4.29E-27
0.002427897	1.75E-06
2.82E-17	3.15E-29
4.73E-08	2.92E-09
2.31E-16	1.74E-31
4.57E-11	1.29E-16
1.15E-05	5.87E-11
8.08E-13	0
1.02E-18	6.04E-33

0.0453143	0.03091459
0.0002459841	2.58E-08
1.73E-14	2.01E-27
2.30E-06	3.29E-12
1.86E-09	8.16E-18
2.37E-15	2.14E-29
0.1004558	0.05384896
1.67E-12	2.75E-22
0.02322576	0.005723184
0.2358055	0.1691342
1.70E-06	5.57E-13
0	0
1.97E-14	1.47E-28
1.55E-10	3.44E-18
0.0001875776	0.000127754
1.66E-07	1.84E-14
8.38E-16	2.12E-25
1.14E-06	3.78E-12
0.0005894705	2.39E-07
0.03013231	0.02196412
45.32291	23.15813
45.90324	24.99188
69.47026	35.29096
48.87722	24.82915
39.14018	19.34993
44.55392	33.41726
47.51948	34.65901
49.04996	32.99404
52.28881	35.3858
46.98986	31.71381
53.37728	39.29238
31.35265	18.61904
4.80E-06	9.78E-11

2.20E-05	3.05E-10
0.0001100519	5.79E-09
28.07632	18.48661
26.70493	16.90196
29.93852	16.22216
5.83E-05	7.11E-10
23.29915	16.08601
2.76E-09	3.04E-18
1.91E-09	8.68E-19
26.12727	18.12511
3.46E-08	1.40E-15
0.0001355109	8.95E-09
24.08865	15.54863
0.0009040885	8.18E-07
1.03E-14	5.62E-27
4.74E-07	1.36E-13
25.2853	17.57219
1.28E-13	6.78E-27
9.87E-06	4.21E-11
0.001036675	2.71E-07
22.0197	15.48144
21.22795	14.79017
27.45308	18.90977
33.93286	18.9259
1.31E-05	2.22E-10
1.037813	0.4438687
26.68628	16.48535
29.29805	16.84602
27.83934	16.67333
27.80965	20.05165
24.73922	16.96271
36.685	20.39548
28.43612	18.13739

25.37825	16.88329
30.55267	17.94632
0.7915384	0.1741021
7.89E-07	2.33E-13
0.06926006	0.03376322
0.1287414	0.06762409
0.09031577	0.04442394
0.03158392	0.0130136
0.118044	0.05977672
0.1363389	0.1057533
0.1098726	0.08024885
0.04320513	0.03090718
0.04124395	0.02812558
0.05455493	0.03604573
0.1281174	0.09902747
0.02748935	0.01098348
0.02806002	0.01385935
0.04094574	0.02105434
0.03016714	0.01519107
0.04810422	0.02195391
0.02015291	0.009501638
0.03000698	0.0176248
0.03778819	0.01519504
0.0361191	0.01765193
0.04701611	0.02270443
0.0272878	0.01470257
0.03899648	0.0158541
0.02737791	0.01576764
0.07339681	0.04096416
0.05244733	0.03116218
0.03269048	0.01849824
0.04242452	0.02113176
0.04361312	0.01774943

0.03932221	0.01847564
0.01879532	0.01012761
0.01603358	0.009688071
0.05483366	0.03181425
0.03504636	0.01794765
0.03184601	0.0138164
0.03083549	0.01578956
0.04160677	0.02006062
0.02725672	0.0141803
0.04270249	0.02087762
0.03987476	0.02022901
0.0563897	0.02754347
0.01888115	0.007769712
0.04742861	0.02643939
0.02479848	0.01394796
0.06212014	0.03390144
0.03429451	0.01363355
0.02904022	0.01298205
0.03323228	0.0161788
0.02961282	0.0126596
0.02915586	0.01725009
0.2644223	0.149616
0.3900828	0.1942568
2.10374	0.8634551
4.351074	1.792337
4.439094	2.11068
0.09359774	0.07198814
0.05995078	0.04665805
0.7594378	0.5042795
0.7953924	0.5500293
1.018272	0.5437706
0.08967026	0.07029061
2.26E-06	1.82E-12

2.66E-12	1.72E-23
4.04E-13	3.26E-24
1.37E-13	9.02E-27
8.82E-11	1.37E-20
1.10E-12	1.33E-23
7.46E-14	3.19E-26
2.31E-13	2.12E-25
1.31E-13	1.50E-25
9.39E-11	1.58E-20
0.04563135	0.0324856
4.98E-12	9.43E-22
4.65E-09	1.59E-17
5.45E-13	8.06E-25
2.86E-12	1.30E-23
2.66E-11	7.19E-22
1.60E-13	3.61E-25
5.99E-13	3.33E-25
1.38E-13	7.56E-26
1.70E-13	5.39E-26
5.64E-11	1.86E-20
2.23E-13	4.69E-24
6.10E-12	5.91E-23
4.30E-13	7.52E-24
2.72E-11	8.62E-21
6.97E-13	2.36E-23
5.85E-13	3.21E-25
2.58E-13	4.58E-26
1.06E-12	2.61E-24
3.24E-13	1.80E-25
1.93E-11	1.44E-21
4.58E-11	4.84E-21
1.28E-13	6.01E-24
2.32E-12	1.36E-22

9.06E-13	2.60E-24
2.32E-14	1.05E-24
9.89E-09	8.73E-17
1.93E-13	1.08E-24
1.78E-10	3.23E-19
7.231372	3.803299
10.34593	5.934351
14.34628	7.556329
42.4878	23.68045
15.04017	8.060049
7.657232	6.019819
7.687237	5.800643
42.06323	29.90578
43.17509	30.73926
37.37564	26.56648
8.347122	6.331308
3.456943	2.348523
2.535902	1.413451
3.051256	1.939428
1.999056	1.133922
3.532435	1.994968
3.508646	1.90294
2.238797	1.101399
3.224776	1.818729
2.339592	1.332425
2.132275	1.389266
2.409859	1.351179
2.056575	1.256791
1.821135	1.05175
2.192806	1.295351
4.320253	2.493655
4.87012	2.774003
3.820607	2.20484

3.263684	2.098685
2.1354	1.193908
1.580078	1.005591
2.589295	1.708902
1.777657	0.9602478
1.834877	1.14872
3.648835	2.391924
2.227191	1.319599
2.584452	1.568645
3.039619	1.773746
2.663158	1.710984
3.014609	1.969887
1.977818	1.332495
2.391853	1.51923
2.214234	1.406529
2.373572	1.409411
3.469404	1.94332
2.347327	1.418818
2.182503	1.189403
3.223726	1.868207
1.971071	1.191532
3.855637	2.221453
0.05871608	0.03415649
0.169854	0.1018899
0.5587382	0.3136099
7.881869	3.280223
2.267637	1.314031
0.413671	0.3293047
0.2342568	0.175183
4.2761	3.087568
3.755362	2.707958
5.493415	3.953205
0.2407932	0.1900671

1.049992	0.6044955
3.20E-14	1.74E-22
4.11E-13	3.03E-20
0.6588724	0.2978363
1.395187	0.5717397
0	0
1.733208	0.6670066
1.562652	0.5179814
3.872974	1.431462
0.5138151	0.3564842
0.5924101	0.4200064
0.7756904	0.5381216
0.6129982	0.416559
3.59E-16	7.76E-29
2.73E-15	1.26E-28
0.4222724	0.2828711
3.80E-17	5.01E-30
3.59E-12	4.17E-21
1.146748	0.5238522
4.83E-15	8.10E-27
4.05E-16	3.14E-31
5.64E-10	7.92E-19
0.3877382	0.2625459
0.5730302	0.4002793
0.8243623	0.5735289
1.302368	0.8767958
0.6020567	0.4061514
3.753643	1.338852
8.58E-16	4.55E-28
1.01E-13	1.86E-24
9.86E-16	5.62E-30
0.8326935	0.3127262
1.78E-15	4.57E-28

1.29482	0.8493207
3.56E-12	1.72E-18
0.8436385	0.5609727
5.72E-14	4.32E-27
4.44E-15	3.24E-20
1.52E-11	1.34E-16
1.585287	0.9045568
1.780925	1.074971
2.343634	1.180968
2.504721	1.346598
2.537368	1.399044
1.40607	1.096416
1.132242	0.8827265
1.200274	0.8672415
1.468079	1.059475
0.9019415	0.6490608
1.301899	1.027638
4.24E-10	2.52E-19
5.31E-12	4.68E-22
4.37E-14	5.14E-27
3.38E-15	4.24E-29
7.97E-14	1.18E-22
1.75E-14	3.85E-27
8.74E-16	6.21E-29
1.63E-13	8.31E-21
2.38E-17	3.44E-32
8.01E-14	4.17E-21
9.82E-15	5.89E-27
1.82E-16	2.65E-27
1.54E-15	1.19E-29
6.56E-13	9.98E-18
9.78E-15	2.93E-28
3.21E-13	5.05E-25

4.37E-16	2.55E-27
7.10E-16	7.70E-26
2.74E-15	9.42E-30
2.87E-17	1.01E-31
1.26E-15	7.65E-27
7.38E-17	7.30E-31
9.47E-17	6.10E-29
9.30E-15	2.68E-27
1.03E-15	1.57E-29
1.11E-21	3.07E-33
3.52E-16	4.03E-28
1.13E-14	7.67E-27
4.65E-14	2.63E-27
0	0
6.15E-16	5.46E-26
8.64E-16	1.78E-30
3.05E-10	8.01E-17
4.17E-16	2.71E-27
2.26E-14	8.14E-26
2.58E-16	1.52E-19
5.51E-15	1.05E-28
3.09E-15	6.98E-16
0.2252606	0.1791142
64.26824	33.82803
29.96677	16.06085
44.86408	22.31345
57.57289	28.83762
46.24242	23.60114
31.54161	23.21067
41.31142	29.91409
25.95341	17.0785
36.31243	24.35308
17.84348	11.53276

30.37138	22.16502
14.19159	7.211742
6.506615	2.761361
5.933457	2.736999
2.156567	0.5985229
7.026419	3.077684
8.262511	3.994444
8.076683	3.129874
14.66094	7.327782
4.001434	1.726211
2.280095	0.7687996
10.51823	5.322815
0.0005917213	2.13E-07
14.45449	6.58872
3.059153	1.309803
14.33465	5.802182
7.283885	2.487539
3.968333	1.406888
9.468807	4.583748
14.81224	7.097047
1.04E-10	5.27E-21
25.09106	13.89139
7.530743	2.994755
1.923584	0.6373807
1.33E-09	1.94E-18
8.265449	3.553647
5.436081	2.445519
4.491319	1.803333
4.127508	1.822673
4.328597	1.867767
2.973946	1.110173
18.06977	9.664253
1.664443	0.547737

5.237451	1.482692
3.198706	1.177058
1.09E-09	2.11E-17
1.389997	0.5943224
4.49E-12	8.23E-23
3.582189	1.259499
5.80E-08	2.22E-15
386.7807	218.5729
429.2742	269.9179
548.147	307.2742
72.35408	37.09624
175.3301	101.9143
188.1834	150.086
168.2049	131.1371
46.82536	33.83302
48.75788	35.18731
49.43773	35.22814
195.29	152.696
45.77565	24.11185
4.30E-19	6.37E-34
2.07E-15	4.54E-25
2.14E-16	6.24E-33
1.23E-15	2.12E-30
3.84E-16	4.06E-30
1.48E-16	2.97E-31
8.62E-17	3.27E-25
5.38E-16	3.03E-31
8.07E-16	4.59E-27
2.91E-16	9.96E-32
1.65E-16	4.41E-32
2.32E-16	1.47E-29
3.17E-16	2.09E-31
1.04E-15	3.02E-31

1.52E-16	1.61E-30
55.29575	36.28422
53.68449	30.85349
1.63E-14	8.70E-22
4.39E-16	2.11E-31
1.42E-16	3.75E-32
1.52E-17	1.27E-32
1.99E-16	2.79E-31
1.16E-14	8.86E-30
3.66E-16	4.86E-31
4.44E-14	6.31E-22
56.70151	29.98285
7.259715	4.927932
6.76E-16	8.86E-30
1.94E-13	1.27E-26
28.75783	20.43903
60.8904	35.96329
2.38E-15	1.17E-30
8.16E-16	2.96E-26
80.04541	45.76144
7.04E-14	2.17E-26
7.45E-17	1.26E-31
5.46E-17	3.23E-31
6.78E-16	3.22E-31
21.03917	11.01274
23.21866	14.26777
34.86677	18.44366
45.65371	24.84472
39.47777	21.1055
13.76075	10.50233
16.30223	12.53228
39.73524	28.46954
41.48019	29.73621

40.58781	28.83256
15.77995	12.45489
35.7866	23.00602
3.47592	1.44017
5.57409	2.977696
3.137836	1.606103
1.925805	0.9656274
4.168555	2.502473
9.57E-12	3.70E-21
0.003386336	0.000183239
4.18E-11	3.59E-20
2.157138	0.7761451
1.921368	1.178728
1.506095	0.8591683
3.84E-12	2.13E-22
2.455135	1.26685
1.697392	0.9452635
3.241186	1.680153
19.47803	12.12262
24.39617	16.26025
2.07E-14	3.19E-26
1.55E-14	1.09E-26
2.443092	1.141637
1.60E-14	1.10E-26
2.65E-13	1.45E-24
7.99E-13	5.41E-24
3.404008	1.314083
3.560646	1.943638
30.887	19.91213
2.00016	0.5043437
4.388102	2.42748
5.444086	2.678339
28.10716	17.78528

29.25026	18.46843
0.1554906	0.1023948
2.528462	1.469604
33.68669	20.34844
1.18E-10	9.59E-20
8.18E-14	8.45E-27
1.66E-14	1.21E-27
9.66E-14	2.55E-27
0	0
0.1772325	0.1115777
0	0
0	0
0	0
0.4315193	0.3442058
0.1116509	0.0870461
0	0
0	0
0	0
0.3884271	0.3066001
0	0
0	0
0	0
0	0
0	0
0	0
0	0
0	0
0	0
0	0
0	0
0	0
0	0
0	0

0	0
0	0
0	0
0	0
0	0
0	0
0	0
0	0
0	0
0	0
0	0
0	0
0	0
0	0
0	0
0	0
0	0
0	0
0	0
0	0
0	0
0	0
0	0
0	0
0	0
0.05665441	0.03295717
0.6148227	0.3870648
0.02704896	0.01520846
0.03467474	0.02004003
0.2408148	0.1399923
0.2316603	0.1847862
0.4253284	0.3315975
0.01233659	0.008913637

0.024664	0.01779938
0.02510267	0.01806454
0.364806	0.2879551
0	0
0	0
0	0
0	0
0	0
0	0
0	0
0	0
0	0
0	0
0	0
0	0
0	0
0	0
0	0
0	0
0	0
0	0
0	0
0	0
0	0
0	0
0	0
0	0
0	0
0	0
0	0
0	0
0	0
0	0

0	0
0	0
0	0
0	0
0	0
0	0
0	0
0	0
0	0
3.25334	1.05528
2.016655	0.8437191
6.814081	3.40127
13.55749	6.466225
6.967084	2.973213
1.958073	0.8851771
1.437229	0.8771941
11.93713	7.159858
10.574	6.221138
8.217009	4.705614
1.198543	0.7466639
6.623982	3.454418
2.932495	0.9725692
0.3459974	0.2282641
1.090185	0.2066634
6.649818	2.134094
2.685764	0.8899448
1.39E-12	1.18E-26
0.5035725	0.3213885
3.734044	1.480257
0.3587563	0.2241877
6.56E-10	1.23E-19
6.40E-10	1.63E-18
1.31E-16	1.13E-32

1.02E-13	5.08E-27
2.67E-08	1.27E-11
4.567423	0.8494489
0.1481251	0.1028893
0.6311854	0.4381943
3.987164	1.907578
0.1034276	0.0765904
8.349328	3.656579
2.004687	1.261524
3.6327	1.247966
9.92E-12	1.10E-19
1.048477	0.7289679
1.73E-15	5.28E-30
0.3553169	0.1719042
1.109232	0.2310194
1.357165	0.9634812
0.3717858	0.2602116
2.857503	1.251822
0.3131838	0.2249747
1.21E-08	4.01E-15
0.002081121	5.22E-05
0.4347519	0.09543644
4.991278	1.605614
0.108858	0.07428791
1.21338	0.4712269
0.3896948	0.3088337
0	0
0	0
0	0
0	0
0	0
0	0
0	0

0	0
0	0
0	0
0	0
0	0
0	0
0	0
0	0
0	0
0	0
0	0
0	0
0	0
0	0
0	0
0	0
0	0
0	0
0	0
0	0
0	0
0	0
0	0
0	0
0	0
0	0
0	0
0	0
0	0
0	0
0	0
0	0
0	0

0	0
0	0
0	0
0	0
0	0
0	0
0	0
0	0
0	0
0	0
0.0574894	0.03344291
0.2276234	0.1433015
0.05143338	0.02891875
0	0
0.3135374	0.182268
0.02205253	0.01759043
0.04469839	0.03484808
0	0
0	0
0	0
0.1165545	0.09200087
0.2803831	0.2095995
0	0
0	0
0	0
0.3211548	0.2200224
0	0
0.09148476	0.06165451
0.3624672	0.2560745
0	0
0.3102323	0.2266691
0	0
0.4462721	0.3095934

0	0
0.3252679	0.2274969
0	0
0	0
0.328158	0.227942
0.276141	0.1921657
0.3921986	0.2725639
0.3632324	0.2689844
0.378917	0.2714276
0.5138642	0.3588826
0	0
0.4227376	0.2953849
0.3017728	0.2099507
0.2404244	0.1669664
0.3382186	0.244374
0.6494722	0.4409894
0.3337219	0.2393658
0	0
0.4035713	0.2933113
0.3903311	0.2817627
0	0
0.4299084	0.2963219
0.5133902	0.344551
0.196044	0.1304233
0	0
0	0
0	0
10.15079	5.112963
23.909	13.71906
14.83396	7.142572
8.635136	3.924324
39.46507	21.43276
13.31647	10.46274

8.102953	6.145215
6.943392	5.01521
7.085996	4.917725
5.944291	4.097299
11.87395	8.747135
2.63807	1.000642
0.06298262	0.04273171
0.05897072	0.04022399
3.92E-16	6.41E-31
4.66E-10	1.01E-15
2.36E-14	4.85E-17
0.1339321	0.09026112
3.56E-15	6.73E-29
2.62E-14	4.28E-27
0.1129405	0.08251085
0.05441094	0.03880881
0.1633027	0.1132715
2.37E-10	1.61E-16
1.16E-07	8.11E-08
1.05E-14	4.48E-28
1.52E-11	1.37E-20
3.730868	1.838081
2.789262	1.130401
2.04E-10	1.41E-20
2.20E-13	2.69E-27
5.20E-10	4.76E-19
1.66E-15	1.36E-29
1.12E-10	3.72E-20
2.10E-11	3.18E-20
3.93E-13	1.85E-24
1.83E-09	6.41E-17
3.491868	1.367472
3.240619	1.112376

3.95E-07	1.06E-07
1.25E-12	1.46E-23
3.671956	1.725745
4.029626	1.596732
0.000175646	2.36E-07
0.07268063	0.0497102
4.126144	1.967055
0.1991505	0.1324899
0.2476294	0.05091638
1.62E-10	1.33E-20
0.008465517	9.52E-05
1.53E-17	2.38E-30
0.01588492	0.01000043
2.21E-19	3.51E-34
3.43E-17	2.22E-32
0.1437367	0.08345288
0.00508663	0.004057403
0.03083763	0.02404185
6.99E-24	1.07E-42
6.19E-24	1.05E-42
1.38E-21	2.38E-39
0.01527204	0.01203531
8.78E-16	6.62E-28
5.96E-13	4.74E-24
5.58E-17	3.60E-31
3.84E-14	1.73E-21
7.73E-09	1.47E-16
7.03E-17	6.80E-32
9.71E-13	2.16E-21
1.32E-16	7.11E-30
5.27E-12	7.21E-22
5.66E-17	1.60E-30
7.51E-15	2.60E-24

4.05E-14	3.13E-28
6.77E-15	7.53E-25
2.74E-13	1.69E-24
1.10E-12	4.12E-24
3.62E-21	1.80E-29
9.10E-16	2.41E-29
4.25E-17	2.43E-29
1.23E-16	1.84E-29
7.89E-13	4.89E-22
2.99E-13	2.43E-21
1.70E-13	2.04E-24
0.01648223	0.01158821
7.28E-13	6.36E-18
4.06E-10	4.27E-16
1.71E-16	4.21E-26
2.76E-12	1.04E-23
3.52E-12	1.81E-23
2.51E-14	3.17E-25
2.97E-13	5.88E-23
6.39E-16	1.88E-30
1.26E-18	3.26E-32
1.29E-13	1.27E-23
6.61E-13	3.66E-24
5.59E-10	1.30E-18
2.01E-15	2.40E-23
4.01E-11	7.36E-18
2.09E-11	6.01E-19
1.37E-09	5.11E-14
5.01E-20	3.41E-36
2.61E-18	7.36E-35
0	0
0	0
0	0

0	0
0	0
0	0
0	0
0	0
0	0
1.01E-15	5.25E-29
8.67E-16	1.32E-28
7.80E-16	3.32E-26
7.67E-16	4.10E-30
2.26E-16	1.43E-30
6.68E-15	1.39E-28
1.20E-15	5.99E-29
2.26E-16	4.49E-30
5.53E-16	1.24E-29
1.95E-16	7.11E-30
2.72E-16	5.02E-30
1.24E-15	3.48E-27
4.16E-16	2.42E-27
2.57E-16	1.79E-29
3.44E-15	7.42E-29
1.78E-15	9.96E-29
1.38E-15	6.73E-29
1.39E-15	7.05E-30
7.40E-16	1.26E-25
1.15E-16	5.87E-30
1.58E-16	6.64E-30
2.23E-16	1.98E-31
1.04E-15	2.17E-29
7.77E-16	4.67E-29
9.39E-17	3.71E-30
5.22E-16	1.51E-27
3.64E-16	1.57E-28

4.87E-16	3.43E-27
1.92E-13	1.31E-24
3.48E-17	3.31E-30
9.98E-15	8.55E-29
3.38E-17	1.96E-30
4.70E-16	7.78E-30
1.66E-15	2.13E-29
1.35E-17	8.12E-30
6.45E-16	5.00E-30
5.67E-16	1.67E-25
6.87E-17	1.39E-26
2.32E-15	4.03E-29
6.258105	3.334673
7.806588	4.500638
9.012843	4.707738
4.466095	2.281621
4.637175	2.417117
9.25381	7.165649
8.341641	6.272295
4.971051	3.386162
4.826512	3.327964
5.255902	3.645921
9.640982	7.331252
1.31E-12	1.73E-23
0.1152357	0.07635004
0.1335467	0.05642038
0.02574274	0.01147947
0.04069644	0.02606929
1.40E-12	2.15E-23
2.42E-13	9.11E-25
0.07164209	0.04071208
1.07E-12	2.66E-23
0.01361037	0.009845113

3.19E-14	1.47E-26
2.11E-13	1.10E-24
7.94E-11	6.13E-20
0.1573197	0.0756576
0.02722496	0.01126754
1.86E-10	6.77E-19
3.21E-11	2.21E-20
0.01664113	0.01145292
0.116408	0.06014174
0.05388138	0.01961442
0.01706685	0.009343815
7.82E-12	1.03E-21
0.01975775	0.01040815
1.20E-11	2.33E-21
0.03225572	0.01857387
0.03788026	0.0105454
1.77E-10	1.53E-18
0.01657208	0.01071839
6.95E-13	5.51E-24
6.16E-11	1.02E-19
6.77E-11	2.56E-19
0.0140468	0.01005741
0.0386613	0.02326285
8.04E-12	1.31E-21
0.1662488	0.1098617
0.03324973	0.02186851
0.01460031	0.009527942
0.01559392	0.01058262
0.04572204	0.03586681
2.148472	1.249576
1.381071	0.7197297
0.1294141	0.07275907
0.1409379	0.08145409

2.148691	1.211947
0.7289024	0.4211757
1.255076	0.897481
0.1460524	0.1055281
0.2076184	0.1498329
0.1842413	0.1242489
0.6456899	0.4989061
0.07653948	0.05721684
0.03788483	0.0223733
0.0960444	0.06551199
0.1091654	0.06379731
0	0
0.06116346	0.01453603
0.1065449	0.07180406
0.0868222	0.06133782
2.75E-06	1.02E-07
0.00154024	5.32E-05
0.0294409	0.02099883
0.000210021	9.19E-08
0.2280614	0.0983596
0.09385894	0.06564625
0	0
0.080479	0.05429002
0	0
0.03304687	0.02299722
0.0297388	0.02066739
0.03024361	0.02090419
0.02929452	0.02098439
0.02793723	0.01951135
0.03346491	0.02127276
7.51E-05	7.87E-09
0.1520663	0.09790724
0	0

0.1004561	0.07258277
0.03212267	0.02181119
7.75E-10	5.55E-10
0.07125921	0.02587467
0.396181	0.09690318
0.05919852	0.04270719
0.2309539	0.04933931
7.59E-07	1.13E-10
4.36E-15	2.07E-29
1.60E-08	1.06E-08
2.11E-18	7.67E-35
8.70E-05	2.89E-08
1.53E-16	2.03E-32
7.540741	3.741724
13.12175	7.115788
16.30194	8.333837
9.31484	4.649065
7.431686	3.579471
7.11645	5.351154
6.437462	4.692461
11.51096	7.876588
11.65443	8.343512
9.499297	6.559475
6.641348	5.018328
1.609434	0.9285499
9.05E-15	8.82E-26
9.89E-15	2.82E-26
2.17E-10	4.15E-19
7.60E-12	2.06E-20
4.65E-13	1.15E-25
5.97E-14	1.74E-25
5.46E-14	4.30E-26
7.64E-14	2.87E-26

1.20E-13	8.41E-27
1.670594	1.080596
7.70E-15	8.59E-27
3.98E-12	2.70E-22
3.10E-12	3.54E-23
8.18E-10	2.18E-16
2.93E-14	1.66E-25
0.3812801	0.1028074
3.362202	1.455999
2.54E-13	1.02E-25
6.59E-15	5.17E-26
1.96E-11	2.60E-21
1.38E-09	8.88E-19
2.60E-13	6.52E-26
3.36E-12	1.08E-23
9.83E-15	1.09E-26
9.80E-13	7.39E-23
1.17E-09	6.03E-18
3.15E-11	6.69E-21
1.95E-10	6.75E-20
1.62E-10	4.33E-17
3.638271	1.827822
2.445618	0.9769389
1.21E-10	7.72E-18
1.66E-13	1.28E-25
4.99E-13	9.01E-25
1.90E-13	1.22E-24
5.36E-14	1.96E-25
6.53E-14	6.46E-26
2.12E-10	9.68E-20
0	0
75.13786	0.616841
0	0

3.31E-20	3.47E-35
0	0
0.2716232	0.2166596
0.8459328	0.6595121
0	0
0	0
0	0
1.904694	1.290869
3753.37	2.279119
2.02E-15	1.89E-30
1.01E-14	3.55E-29
3.49E-08	9.51E-16
0.0001106737	3.38E-07
0.0002637977	5.31E-07
1.455781	0.07049645
8.09E-09	3.59E-11
19.58356	4.354689
5.37E-16	1.01E-29
31.47975	0.4624422
4.88E-15	7.82E-30
2.20E-09	4.35E-19
5.96E-10	1.16E-17
0.7158014	0.01931005
9.92E-15	5.67E-30
8.90E-16	2.99E-29
6.84E-09	1.90E-13
7.72E-16	3.77E-28
9.53E-08	5.90E-19
3.82E-16	8.76E-32
2.84E-16	3.09E-29
1.03E-13	2.21E-27
7.57E-14	1.29E-27
7.24E-14	5.93E-28

2.51E-15	4.11E-30
6.57E-16	9.11E-31
3.55E-15	3.40E-29
3.59E-14	1.73E-28
5.14E-11	1.42E-18
5.19E-12	5.76E-25
0.0008719618	1.23E-05
6.83E-08	6.20E-15
4.85E-15	5.35E-30
7.65E-16	2.02E-31
1.70E-12	5.19E-24
6.32E-14	6.86E-25
5.61E-16	8.16E-29
1.57E-14	4.03E-30
71.74071	26.53199
73.29789	25.3275
45.96333	17.5293
34.72773	8.225654
56.89384	17.84293
15.70178	7.173593
15.51026	11.52368
3.872573	2.767087
5.935644	3.579211
1.202104	0.8625939
17.94376	11.19157
7.902616	5.803283
11.70495	7.941041
9.281338	4.940589
10.9527	7.354308
3.339913	2.288158
7.603751	3.36055
2.01E-05	4.88E-12
6.293994	2.432188

2.50E-12	4.15E-25
0.0002602289	1.38E-09
4.59E-10	2.82E-21
4.661239	3.160686
3.129249	1.654117
8.77E-07	7.68E-15
0.005168491	5.86E-07
3.824111	2.340394
6.494056	2.783382
5.31006	3.680577
0.01575484	3.09E-06
10.69115	7.669662
3.60E-09	7.32E-18
9.319826	6.108128
1.46E-11	5.78E-24
0.01935532	7.30E-06
4.007255	2.322402
17.49103	2.749414
9.197773	6.481321
14.16709	2.542898
9.965622	3.885089
8.23E-13	7.23E-27
1.40E-07	1.75E-14
2.294486	1.64226
5.174988	2.786855
20.77707	5.859398
1.483261	0.8238182
7.41262	0.4103161
4.590165	2.450811
17.31357	6.01816
0.0008883648	7.01E-08
14.95113	8.449892
20.04618	6.766548

4.558881	2.28097
12.72459	7.343597
25.86062	8.487955
8.560978	6.775024
6.283828	4.899042
2.108976	1.523812
4.266244	3.078839
2.172976	1.560521
8.133452	5.706018
3.151156	2.352649
3.59E-15	1.88E-28
7.04E-15	5.27E-29
2.71E-16	2.92E-32
4.93E-15	6.19E-30
3.22E-15	2.71E-29
2.47E-15	2.49E-30
2.92E-14	4.39E-27
1.17E-16	6.93E-31
6.27E-18	5.51E-32
1.76E-14	6.77E-29
1.20E-14	6.63E-27
7.81E-15	4.24E-28
5.70E-15	3.13E-29
4.34E-14	5.36E-28
6.53E-15	1.12E-29
7.479869	4.804248
13.86583	9.013568
5.12E-14	1.31E-28
8.55E-14	7.81E-26
2.21E-15	3.89E-29
5.32E-16	9.02E-32
1.43E-15	4.60E-31
1.02E-14	1.31E-24

1.31E-14	2.93E-29
8.02E-16	3.50E-31
13.8992	5.928272
4.40E-14	1.82E-24
8.90E-15	2.11E-28
8.35E-15	8.26E-28
17.19367	6.814042
15.23513	6.102944
4.98E-15	0
2.61E-10	1.79E-18
16.47204	10.90799
7.48E-15	7.14E-30
1.40E-15	1.28E-30
7.51E-10	1.33E-17
7.60E-13	1.74E-23
19.55279	9.827272
24.42045	13.39921
22.42356	12.53261
21.75772	12.57289
23.20416	13.27286
3.53451	2.819339
3.521484	2.745444
4.810671	3.475885
5.950598	4.294395
2.716894	1.955148
5.016275	3.761023
4.674296	3.270285
3.270495	2.21893
0.3956469	0.2688071
4.292391	2.393309
3.959653	2.46961
0	0
0.4510819	0.3039985

1.921892	1.300091
5.209958	1.766182
2.337584	1.655845
1.59E-13	6.21E-25
5.06E-09	2.75E-18
5.770144	2.765168
9.781986	4.516417
0.8942193	0.5829741
1.07E-12	2.83E-24
2.57E-12	1.97E-23
0.4212437	0.2931422
1.14E-12	1.63E-25
10.15713	4.386054
8.431719	3.663943
6.776219	4.638938
0.8276883	0.5805462
2.54E-07	1.91E-15
7.414313	2.806245
4.781777	2.094017
5.995786	1.560667
1.82229	1.237329
4.631335	3.227268
0.5103292	0.3517618
0.5536002	0.4023507
5.693151	3.69538
2.18E-13	2.33E-25
4.45E-14	1.31E-23
5.780712	3.818906
2.252556	1.49857
1.91E-13	2.28E-24
5.876938	3.988651
1.26E-12	7.45E-25
0	0

0	0
6.65E-21	6.20E-36
0	0
0.4381303	0.2546972
0	0
0	0
0	0
0	0
0	0
0	0
0.1113687	0.08325285
0	0
0.3308655	0.2049441
9.69E-17	2.29E-29
1.32E-11	1.31E-21
2.09E-15	8.13E-26
0.6759381	0.4493528
0.0801313	0.05661085
4.88E-09	3.37E-09
0.3618612	0.2512037
1.156055	0.1427627
0.2699434	0.1496561
1.140965	0.7832488
2.89E-21	1.37E-40
0.1358101	0.07719347
0.5796916	0.1160049
2.48E-10	2.01E-13
1.80E-16	4.45E-32
2.186746	0.6069503
0	0
0	0
0.5558474	0.3293505
0.860888	0.4363634

8.07E-10	5.64E-10
8.88E-12	1.20E-18
0.1833841	0.1272677
0	0
1.195702	0.8118773
3.79E-30	6.97E-61
0	0
0	0
0.08615637	0.061987
0.1069364	0.07318254
0.1004796	0.06706324
1.65E-28	1.86E-56
0.1102883	0.07337211
2.73E-16	2.24E-30
1.03E-25	7.95E-49
1.82E-20	1.60E-38
16.036	8.071169
25.72425	14.56789
12.58701	6.141084
17.27389	8.626166
10.12568	4.90841
4.297039	3.134744
7.036252	4.818599
8.720533	5.673359
7.634462	4.950614
10.05306	6.371423
4.39985	2.920582
2.573771	1.152835
0.606018	0.1888153
1.287477	0.5832386
1.799535	0.7154611
2.314172	0.8144325
1.365183	0.4293882

1.090873	0.402396
2.43E-08	8.03E-14
4.87E-13	2.42E-25
3.11E-08	4.79E-15
1.112936	0.4918734
1.587732	0.8198817
1.94E-13	5.44E-26
2.020633	0.7512666
7.14E-11	1.09E-20
0.6656054	0.4261252
9.01E-12	9.82E-23
2.02E-14	1.76E-28
1.16E-09	2.55E-17
0.7056847	0.3746933
1.13E-09	3.37E-18
2.050448	0.9141638
0.7969709	0.4386697
4.28E-10	2.60E-19
2.44E-11	6.43E-20
0.6557942	0.3256747
1.03E-12	3.56E-25
2.456464	1.041828
1.082209	0.5163745
1.62E-11	8.65E-22
1.728411	0.4792077
1.174718	0.4707385
1.22E-08	3.25E-16
1.494186	0.667372
1.262106	0.4597099
0.7144856	0.216382
1.00E-14	5.65E-27
0.9164472	0.298852
3.94E-16	8.03E-31

27.77054	14.04781
19.81028	10.29678
44.94524	22.88688
16.55421	7.692716
32.27593	16.12922
31.51056	23.64561
32.36582	23.81208
17.4831	11.45664
17.60968	11.67595
16.43357	10.86364
39.30241	29.26474
20.22515	11.46004
11.54274	5.784375
15.76584	8.587605
11.51408	5.471742
19.57256	11.05227
20.08086	10.92473
13.42266	7.059452
13.57928	7.394378
9.232549	4.827702
8.396013	4.601652
12.82358	7.619673
6.05E-08	9.37E-15
11.83932	6.310415
9.994436	5.207467
14.44248	7.269028
9.674673	4.411443
10.17269	5.113616
12.73527	7.370587
22.54744	12.90744
8.58E-11	2.32E-20
17.3788	10.10792
14.14204	8.054221

6.848841	3.340192
9.33E-11	3.10E-20
21.11889	11.72739
12.60105	6.850993
12.21381	6.776726
23.6289	12.99834
12.31824	6.185273
11.59035	6.547173
18.79107	10.52124
7.858597	3.712894
10.92364	4.949789
12.75282	6.793044
1.05E-10	4.37E-21
13.15462	6.61141
2.19E-11	3.16E-21
8.370761	4.049065
1.73E-08	1.12E-15
19.80566	9.842019
24.81297	13.67466
7.794856	3.609858
13.31897	6.343982
16.01291	7.634772
15.61229	11.51101
14.49449	10.43682
14.70983	9.766302
13.65218	8.959991
15.34555	10.16214
16.26402	11.82822
0.4140853	0.1999274
1.45E-09	2.60E-14
3.66E-10	2.10E-19
2.36E-11	4.41E-19
1.61E-09	4.52E-17

7.59E-08	1.57E-14
2.62E-08	3.44E-16
2.72E-09	5.58E-17
2.31E-09	2.01E-17
5.15E-10	1.42E-18
9.61E-10	5.67E-18
1.01E-11	1.12E-21
1.12E-11	7.75E-22
1.07E-13	4.43E-19
3.72E-09	3.14E-17
4.43E-12	7.57E-23
5.77E-10	1.81E-18
3.00E-10	6.78E-19
0.1761704	0.0847182
4.65E-13	4.25E-24
1.17E-09	1.82E-17
1.84E-10	6.73E-19
2.30E-10	4.31E-19
4.01E-12	6.83E-23
2.59E-09	3.66E-17
2.44E-09	2.65E-17
2.28E-09	5.73E-17
4.62E-12	1.49E-22
3.14E-09	1.25E-16
1.73E-10	1.67E-19
9.66E-12	1.26E-19
5.25E-10	2.06E-19
8.44E-09	2.73E-09
4.56E-12	2.96E-22
2.52E-10	1.97E-18
3.39E-08	2.07E-08
4.70E-10	9.67E-13
1.55E-13	1.77E-20

6.25E-11	1.18E-19
0.0003457964	0.000201151
0.003497744	0.0013112
0.0006497741	0.000364878
0.001442236	0.000528715
0.0004915105	0.000285092
9.61E-34	2.28E-64
8.96E-07	2.20E-08
6.82E-28	1.41E-41
0	0
0.0003076264	9.55E-05
0	0
0.086812	0.04310746
0.08121648	0.03659968
0.1109353	0.05281631
0.0379262	0.01607115
0.3912376	0.2197807
0.1038094	0.05023759
0.2002075	0.1020611
0.02295725	0.01055122
0.1045154	0.05039871
0.1057725	0.05227244
0.09044418	0.04205135
0.08774607	0.04185247
0.09124059	0.04269918
0.03908597	0.01472365
0.2248167	0.1154634
0.2679352	0.1501542
0.03954566	0.0168798
0.0538741	0.02408544
0.04701233	0.01903849
0.04888214	0.02125843
0.04911561	0.02344252

0.05156698	0.02422621
0.05584294	0.02313508
0.1023655	0.04543194
0.09045937	0.04243185
0.04343439	0.01566357
0.07632575	0.03360424
0.0748772	0.02576741
0.03775583	0.01369338
0.02586881	0.01098602
0.0336861	0.01266355
0.08756714	0.04288702
0.1263047	0.06088379
0.06171243	0.02540535
0.1121641	0.05190802
0.09510654	0.04288387
0.1112046	0.05331022
0.09013669	0.04242804
0.1398822	0.07544755
6.024166	3.504396
22.24461	14.00358
11.54222	6.489689
0.6323582	0.3654359
4.522091	2.628756
6.067059	4.839453
4.380098	3.414843
0.4577862	0.3307672
0.5933954	0.4282383
0.7369776	0.5303484
5.540932	4.373666
1.083474	0.8096872
0.4248314	0.2882334
3.518851	2.399816
2.127175	1.462095

3.078935	1.374034
6.29E-17	2.59E-33
4.241553	2.858519
0.584462	0.4053757
0.2063945	0.1424589
1.819222	1.329168
1.205785	0.8575648
2.524548	0.7817433
4.44174	2.947575
3.173158	2.219285
1.58013	1.030108
0.7570513	0.5168988
0.4879724	0.3389262
3.27335	2.277679
0.3351736	0.2329335
1.144476	0.8475136
1.849906	1.322901
1.21E-23	2.80E-42
1.08E-14	1.92E-26
4.08E-16	1.28E-31
0.1757151	0.1220252
0.202296	0.14046
2.09E-17	8.36E-32
5.125698	3.478951
2.285086	1.638932
1.725877	1.239219
0.7648076	0.5558529
2.199688	1.586987
1.73E-15	1.18E-27
1.463348	0.9893748
2.064204	1.385271
2.97178	1.976888
1.838263	1.253078

5.314529	1.38692
2.08E-16	1.10E-31
0.135028	0.02618296
2.97E-19	1.05E-34
2.10E-19	3.21E-38
0	0
0	0
0	0
0	0
0	0
0	0
0	0
0.1073428	0.02589964
0	0
0	0
0	0
0	0
0	0
0	0
0	0
0	0
0	0
0	0
0	0
0	0
0	0
0	0
0	0
0	0
0	0
0	0
0	0
0	0

0	0
0	0
4.67E-19	6.23E-36
1.03E-18	1.65E-31
1.96E-17	2.02E-37
4.79E-32	1.98E-64
0	0
0	0
0	0
0	0
0	0
0	0
2.47E-19	9.55E-42
0	0
0	0
2.36E-19	1.29E-29
0	0
6.18E-28	9.90E-42
0	0
30.32207	16.54739
51.16537	30.13129
63.02945	33.85597
4.411694	2.133305
16.45644	8.778278
10.44387	7.758238
23.12929	17.24475
1.827953	1.315051
2.569655	1.646923
1.685342	1.188105
18.42816	13.70855
1.875269	0.5943752
0.4334782	0.2925697
0.1094479	0.0732107

5.28E-15	4.92E-28
1.178349	0.6654842
0.1432546	0.09886125
0.2422935	0.1214566
5.28E-05	8.80E-10
0.229286	0.1576941
1.488295	0.7919609
2.540887	1.091601
1.228576	0.4276896
9.66E-11	1.03E-18
0.001294245	1.08E-06
1.46E-11	4.64E-19
4.58E-15	6.98E-29
1.981849	0.4095121
0.03122258	0.000783839
0.05216785	0.03482645
9.02E-11	1.31E-18
0.06863918	0.03590727
0.3592888	0.1309271
3.09E-15	2.81E-27
4.17E-13	3.89E-25
0.8416164	0.2770066
0.3225589	0.1677119
0.3582772	0.1785868
1.484758	0.5079117
1.976389	0.9454063
1.55908	0.3860175
0.499064	0.07953507
0.287168	0.1058166
4.44E-14	5.28E-24
0.3887085	0.1508374
5.10E-12	1.20E-22
7.76E-15	3.39E-28

0.05112759	0.03490033
1.6365	0.1586464
0.05116551	0.04048043
0.08754792	0.05092863
0.3085537	0.1919397
0	0
0	0
0.06093064	0.03542066
0.1139601	0.09090126
0.2095386	0.1633619
0	0
0	0
0	0
0.2369309	0.1868175
1.86E-30	4.11E-62
0	0
0	0
0	0
0	0
0	0
0	0
4.18E-28	5.85E-58
0	0
0	0
0	0
0	0
0	0
0	0
0	0
0	0
0	0
0	0
0	0

7.43E-20	1.23E-20
2.54E-25	5.32E-50
0	0
0	0
0	0
1.70E-27	3.44E-56
4.11E-23	1.29E-45
0	0
4.06E-20	9.52E-40
0	0
0	0
0	0
3.45E-30	1.18E-61
0	0
4.96E-05	8.21E-07
0	0
0	0
1.96E-16	7.03E-32
0	0
2.36E-20	3.66E-38
0.02224464	0.01294022
0.05971181	0.0375908
1.47E-19	1.19E-34
0	0
1.25E-11	1.25E-15
0.03022559	0.02410975
0.01015246	0.007915132
0	0
0	0
0	0
0	0
0.8838816	0.2204898
0.03117362	0.02115043

1.28E-24	2.21E-46
3.80E-12	3.72E-23
0.0389912	0.02647261
0	0
3.72E-23	1.55E-44
9.44E-22	2.27E-25
0.03258127	0.02249451
0	0
8.20E-28	7.19E-57
0	0
9.12E-26	1.06E-48
0	0
0	0
0	0
3.54E-07	4.00E-08
0	0
0.1494518	0.1038636
9.50E-29	1.21E-57
1.49E-21	1.46E-41
0.0007495903	2.10E-05
0.02971799	0.02089391
1.39E-23	4.21E-39
1.45E-14	3.04E-26
4.18E-20	2.58E-34
0	0
9.03E-29	3.37E-54
0	0
0	0
1.06E-29	6.68E-61
0	0
3.58E-19	3.62E-34
0.04550266	0.02157229
0	0

3.84E-29	7.90E-60
6.78E-29	2.86E-55
2.81E-20	1.20E-28
6.24E-23	1.30E-39
16.58934	8.940462
43.0231	25.6461
32.78124	17.47394
7.939705	4.141606
23.7455	12.67891
16.86131	13.03383
16.47745	12.56747
10.10264	7.246543
11.40491	8.00698
11.43342	8.134939
20.40959	15.60887
12.21564	7.9568
8.36E-12	8.19E-22
3.07E-06	2.31E-11
0.7017808	0.1789419
15.12765	9.271674
16.59045	9.997635
24.04702	14.45433
1.05E-09	7.42E-10
15.68585	9.825517
4.71E-13	2.51E-24
3.57E-14	7.87E-27
18.58214	11.33931
0.04867507	0.006874251
0.03204345	0.004449701
14.52705	8.438293
4.99E-13	6.30E-25
4.36E-10	5.70E-20
3.92E-12	4.65E-23

11.84425	6.955097
1.34E-11	4.85E-21
5.22E-11	2.31E-21
3.83E-14	1.07E-26
11.09725	6.789791
15.57507	9.376939
17.42074	10.69212
16.79649	10.29664
2.37E-09	2.87E-17
0.5815699	0.1490055
18.78627	11.78177
11.04872	6.657562
13.89332	8.67847
12.54615	8.003352
14.85257	9.091167
16.80359	10.42096
13.70377	8.001247
16.63868	9.561533
15.08086	9.170297
0.05193636	0.02820826
0.09468208	0.07443225
0	0
0	0
0	0
0	0
0	0
0	0
0	0
0	0
0	0
0	0
0	0
0	0

0	0
0	0
0	0
0	0
0	0
0	0
0	0
0	0
0	0
0	0
0	0
0	0
0	0
0	0
0	0
0	0
0	0
0	0
0	0
0	0
0	0
0	0
0	0
2.57E-31	1.48E-62
0	0
0	0
0	0
0	0
0	0
0	0
0	0
0	0
0	0

0	0
0	0
0	0
0	0
0	0
5.219475	2.722985
5.95336	3.19216
2.51085	1.124289
3.561129	1.92724
8.438082	4.452366
4.451753	3.336726
5.524604	4.013677
4.097071	2.739038
4.332547	2.898997
3.078332	2.071412
4.476861	3.503198
0.4203231	0.2582442
0.3598401	0.227889
1.105593	0.4182219
0.6067761	0.4132011
1.19891	0.555058
1.055322	0.5885842
0.6293072	0.2928192
0.4065507	0.267073
0.6492923	0.2340962
0.4324869	0.2389532
0.4364493	0.3063143
0.1076784	0.06764327
0.6733309	0.2523903
0.3930688	0.2606973
0.4469021	0.2850429
0.3953138	0.2644425
0.08794489	0.06084863

0.08390931	0.0540114
0.732808	0.4665803
0.4229989	0.2850793
0.9168641	0.2948885
0.9090468	0.4868393
0.2940292	0.2022659
0.9315249	0.5716185
0.3243383	0.201866
0.6297688	0.2133985
0.2090229	0.1379745
0.7553543	0.341583
0.5215305	0.3436159
0.2636037	0.1689856
0.9277594	0.526739
0.2747355	0.1951015
0.3113082	0.193166
0.4120583	0.2758656
0.334829	0.2109572
1.312718	0.7413677
0.7341504	0.4926313
0.5988702	0.3323507
0.5280233	0.357883

Supplementary Table 4: Gene expression values for 3 genes present in Tapidor and predicted to be absent in Darmor using RNA-Seq data from Darmor, T

Gene	Library	Unique Counts	FPKM	FPKM Confidence Low
BnaA05g23840.1T	Darmor_AUP_AOSW_2_D09BTA	0	0	0
	Darmor_AUP_BOSW_3_D09BTA	0	0	0
	Darmor_AUP_DOSW_2_D09BTA	0	0	0
	Darmor_AUP_FOSW_4_D09BTA	0	0	0
	Darmor_AUP_HOSW_5_D09BTA	0	0	0
	Darmor_AUP_IAOSW_1_1_C0U	0	0	0
	Darmor_AUP_KAOSW_2_1_C0U	0	0	0
	Darmor_AUP_LAOSW_3_1_C0U	0	0	0
	Darmor_AUP_MAOSW_4_1_C0	0	0	0
	Darmor_AUP_NAOSW_5_1_C0U	0	0	0
	Darmor_AUP_PAOSW_6_1_C0U	0	0	0
	Ningyou_ERR037335	0	0	0
	TN04_ERR037297	0	0	0
	TN05_ERR037298	0	0	0
	TN08_ERR037301	0	0	0
	TN103_ERR037317	0	0	0
	TN108_ERR037318	0	0	0
	TN109_ERR037319	0	0	0
	TN10_ERR037294	0	0	0
	TN121_ERR037320	0	0	0
	TN124_ERR037321	0	0	0
	TN126_ERR037322	0	0	0
	TN128_ERR037323	0	0	0
	TN129_ERR037324	0	0	0
	TN12_ERR037302	0	0	0
	TN138_ERR037325	0	0	0
	TN149_ERR037326	0	0	0
	TN161_ERR037327	0	0	0
	TN163_ERR037328	0	0	0
	TN170_ERR037329	0	0	0
	TN171_ERR037332	0	0	0

	TN172_ERR037333	0	0	0
	TN176_ERR037330	0	0	0
	TN177_ERR037331	0	0	0
	TN24_ERR037299	0	0	0
	TN28_ERR037295	0	0	0
	TN30_ERR037305	0	0	0
	TN31_ERR037303	0	0	0
	TN38_ERR037304	0	0	0
	TN44_ERR037306	0	0	0
	TN48_ERR037307	0	0	0
	TN51_ERR037308	0	0	0
	TN57_ERR037309	0	0	0
	TN72_ERR037312	0	0	0
	TN76_ERR037311	0	0	0
	TN77_ERR037310	0	0	0
	TN85_ERR037315	0	0	0
	TN88_ERR037313	0	0	0
	TN90_ERR037316	0	0	0
	Tapidor_ERR037334	0	0	0
BnaC02g32930.1T	Darmor_AUP_AOSW_2_D09BTA	0	0	0
	Darmor_AUP_BOSW_3_D09BTA	0	0	0
	Darmor_AUP_DOSW_2_D09BTA	0	0	0
	Darmor_AUP_FOSW_4_D09BTA	0	0	0
	Darmor_AUP_HOSW_5_D09BTA	0	0	0
	Darmor_AUP_IAOSW_1_1_C0U	0	0	0
	Darmor_AUP_KAOSW_2_1_C0U	0	0	0
	Darmor_AUP_LAOSW_3_1_C0U	0	0	0
	Darmor_AUP_MAOSW_4_1_C0	0	0	0
	Darmor_AUP_NAOSW_5_1_C0U	0	0	0
	Darmor_AUP_PAOSW_6_1_C0U	0	0	0
	Ningyou_ERR037335	0	0	0
	TN04_ERR037297	0	0	0
	TN05_ERR037298	0	0	0

	TN08_ERR037301	0	0	0
	TN103_ERR037317	0	0	0
	TN108_ERR037318	0	0	0
	TN109_ERR037319	0	0	0
	TN10_ERR037294	0	0	0
	TN121_ERR037320	0	0	0
	TN124_ERR037321	0	0	0
	TN126_ERR037322	0	0	0
	TN128_ERR037323	0	0	0
	TN129_ERR037324	0	0	0
	TN12_ERR037302	0	0	0
	TN138_ERR037325	0	0	0
	TN149_ERR037326	0	0	0
	TN161_ERR037327	0	0	0
	TN163_ERR037328	0	0	0
	TN170_ERR037329	0	0	0
	TN171_ERR037332	0	0	0
	TN172_ERR037333	0	0	0
	TN176_ERR037330	0	0	0
	TN177_ERR037331	0	0	0
	TN24_ERR037299	0	0	0
	TN28_ERR037295	0	0	0
	TN30_ERR037305	0	0	0
	TN31_ERR037303	0	0	0
	TN38_ERR037304	0	0	0
	TN44_ERR037306	0	0	0
	TN48_ERR037307	0	0	0
	TN51_ERR037308	0	0	0
	TN57_ERR037309	0	0	0
	TN72_ERR037312	0	0	0
	TN76_ERR037311	0	0	0
	TN77_ERR037310	0	0	0
	TN85_ERR037315	0	0	0

	TN88_ERR037313	0	0	0
	TN90_ERR037316	0	0	0
	Tapidor_ERR037334	0	0	0
BnaA02g09400.1T	Darmor_AUP_AOSW_2_D09BTA	0	0	0
	Darmor_AUP_BOSW_3_D09BTA	0	0	0
	Darmor_AUP_DOSW_2_D09BTA	0	7.33E-29	0
	Darmor_AUP_FOSW_4_D09BTA	0	0	0
	Darmor_AUP_HOSW_5_D09BTA	0	0	0
	Darmor_AUP_IAOSW_1_1_C0U	0	0	0
	Darmor_AUP_KAOSW_2_1_C0U	0	0	0
	Darmor_AUP_LAOSW_3_1_C0U	0	0	0
	Darmor_AUP_MAOSW_4_1_C0	0	0	0
	Darmor_AUP_NAOSW_5_1_C0U	0	0	0
	Darmor_AUP_PAOSW_6_1_C0U	0	0	0
	Ningyou_ERR037335	0	2.45E-44	0
	TN04_ERR037297	0	0	0
	TN05_ERR037298	0	0	0
	TN08_ERR037301	0	0	0
	TN103_ERR037317	0	0	0
	TN108_ERR037318	0	0	0
	TN109_ERR037319	0	0	0
	TN10_ERR037294	0	1.44E-47	0
	TN121_ERR037320	0	0	0
	TN124_ERR037321	0	0.2951459	0.2951459
	TN126_ERR037322	0	4.52E-31	0
	TN128_ERR037323	3	0.8904273	0.8904273
	TN129_ERR037324	0	0	0
	TN12_ERR037302	0	0	0
	TN138_ERR037325	0	0	0
	TN149_ERR037326	0	0	0
	TN161_ERR037327	0	0	0
	TN163_ERR037328	0	0	0
	TN170_ERR037329	0	1.97E-49	0

	TN171_ERR037332	0	0	0
	TN172_ERR037333	0	3.24E-48	0
	TN176_ERR037330	0	0	0
	TN177_ERR037331	0	2.40E-38	0
	TN24_ERR037299	0	0	0
	TN28_ERR037295	0	5.56E-40	0
	TN30_ERR037305	0	0	0
	TN31_ERR037303	0	8.09E-37	0
	TN38_ERR037304	0	0	0
	TN44_ERR037306	0	0	0
	TN48_ERR037307	0	0	0
	TN51_ERR037308	0	0	0
	TN57_ERR037309	0	0	0
	TN72_ERR037312	0	0	0
	TN76_ERR037311	0	0	0
	TN77_ERR037310	0	0	0
	TN85_ERR037315	0	0	0
	TN88_ERR037313	0	0	0
	TN90_ERR037316	0	1.14E-49	0
	Tapidor_ERR037334	0	1.90E-19	0

Tapidor, Ningyou-7 and the Tapidor x Ningyou-7 population

FPKM Confidence High	TPM
0	0
0	0
0	0
0	0
0	0
0	0
0	0
0	0
0	0
0	0
0	0
0	0
0	0
0	0
0	0
0	0
0	0
0	0
0	0
0	0
0	0
0	0
0	0
0	0
0	0
0	0
0	0
0	0
0	0
0	0
0	0

0	0
0	0
0	0
0	0
0	0
0	0
0	0
0	0
0	0
0	0
0	0
0	0
0	0
0	0
0	0
0	0
0	0
0	0
0	0
0	0
0	0
0	0
0	0
0	0
0	0
0	0
0	0
0	0
0	0
0	0
0	0
0	0
0	0

0	0
0	0
0	0
0	0
0	0
0	0
0	0
0	0
0	0
0	0
0	0
0	0
0	0
0	0
0	0
0	0
0	0
0	0
0	0
0	0
0	0
0	0
0	0
0	0
0	0
0	0
0	0
0	0
0	0
0	0
0	0
0	0
0	0

0	0
0	0
0	0
0	0
0	0
1.49E-14	3.81E-29
0	0
0	0
0	0
0	0
0	0
0	0
0	0
0	0
1.71E-22	1.59E-44
0	0
0	0
0	0
0	0
0	0
0	0
5.38E-24	8.95E-48
0	0
0.2951459	0.1870473
3.52E-18	2.82E-31
0.8904273	0.5387529
0	0
0	0
0	0
0	0
0	0
0	0
1.83E-25	1.21E-49

0	0
1.17E-24	2.02E-48
0	0
4.78E-20	1.48E-38
0	0
4.50E-20	3.43E-40
0	0
1.17E-18	5.08E-37
0	0
0	0
0	0
0	0
0	0
0	0
0	0
0	0
0	0
0	0
9.32E-28	6.88E-50
2.22E-12	1.31E-19

Supplementary Table 5: TopGO enriched terms for genes absent in Tapidor but present in Darmor

GO ID	Term	Annotated	Significant	Expected	p-value
GO:0000375	RNA splicing, via transesterification re...	401	4	0.25	5.50E-05
GO:0045694	regulation of embryo sac egg cell	11	1	0.01	0.0068
GO:0010451	floral meristem growth	14	1	0.01	0.0086
GO:0010501	RNA secondary structure unwinding	229	2	0.14	0.0088
GO:0010450	inflorescence meristem growth	15	1	0.01	0.0092
GO:0000023	maltose metabolic process	17	1	0.01	0.0104
GO:0010165	response to X-ray	20	1	0.01	0.0122
GO:0033388	putrescine biosynthetic process from arg...	21	1	0.01	0.0128
GO:0050982	detection of mechanical stimulus	28	1	0.02	0.0171
GO:0042732	D-xylose metabolic process	30	1	0.02	0.0183
GO:0080148	negative regulation of response to water...	32	1	0.02	0.0195
GO:0006821	chloride transport	40	1	0.02	0.0243
GO:0009969	xyloglucan biosynthetic process	42	1	0.03	0.0255
GO:0036065	fucosylation	46	1	0.03	0.0279
GO:0007062	sister chromatid cohesion	55	1	0.03	0.0333
GO:0015693	magnesium ion transport	55	1	0.03	0.0333
GO:0010082	regulation of root meristem growth	57	1	0.04	0.0345
GO:0000387	spliceosomal snRNP assembly	59	1	0.04	0.0357
GO:1903830	magnesium ion transmembrane transport	61	1	0.04	0.0369
GO:0006108	malate metabolic process	66	1	0.04	0.0398
GO:0000122	negative regulation of transcription fro...	68	1	0.04	0.041
GO:0009311	oligosaccharide metabolic process	294	2	0.18	0.0411
GO:0009098	leucine biosynthetic process	74	1	0.05	0.0446
GO:0006825	copper ion transport	74	1	0.05	0.0446
GO:0015766	disaccharide transport	75	1	0.05	0.0452
GO:0006098	pentose-phosphate shunt	77	1	0.05	0.0463
GO:0005983	starch catabolic process	78	1	0.05	0.0469
GO:0016575	histone deacetylation	91	1	0.06	0.0545
GO:0006397	mRNA processing	1010	4	0.62	0.0554
GO:0051260	protein homooligomerization	97	1	0.06	0.058

Supplementary Table 6: Number and total sizes of collapsed regions in Darmor and Tapidor

Name	Collapsed Darmor total size	Collapsed Tapidor total size	Collapsed Darmor regions	Collapsed Tapidor regions	Size per region in Darmor	Size per region in Tapidor
chrA01	987	1,471	351,921	697,674	357	474
chrA02	1,257	2,387	564,209	1,394,761	449	584
chrA03	1,122	1,907	609,608	1,221,015	543	640
chrA04	770	701	348,334	261,085	452	372
chrA05	725	739	275,928	309,924	381	419
chrA06	1,426	964	586,903	313,083	412	325
chrA07	776	726	304,470	245,872	392	339
chrA08	506	448	195,086	167,022	386	373
chrA09	2,033	1,293	824,647	447,826	406	346
chrA10	475	584	183,768	170,122	387	291
Total A	10,077	11,220	4,244,874	5,228,384		
chrC01	1,630	1,233	634,857	404,581	389	328
chrC02	2,159	2,160	801,588	933,152	371	432
chrC03	2,023	1,765	808,234	496,421	400	281
chrC04	1,951	1,669	744,602	509,246	382	305
chrC05	1,339	1,478	502,906	410,433	376	278
chrC06	1,201	1,111	453,884	350,364	378	315
chrC07	1,281	1,286	478,504	388,908	374	302
chrC08	1,240	1,461	451,909	509,657	364	349
chrC09	1,874	1,767	731,949	499,296	391	283
Total C	14,698	13,930	5,608,433	4,502,058		
Unplaced	5,425	1,662	2,642,537	791,647	487	476
Total	30,200	26,812	12,495,844	10,522,089		

Supplementary Table 7: Number and total sizes of repetitive regions in Darmor and Tapidor

Chromosome	Repetitive Darmor regions	Repetitive Tapidor regions	Repetitive Darmor total size	Repetitive Tapidor total size	Size per region in Darmor	Size per region in Tapidor
chrA01	1,725	165	1,322,478	87,648	767	531
chrA02	1,751	95	1,147,730	37,037	655	390
chrA03	1,936	111	1,382,485	41,601	714	375
chrA04	1,318	94	946,791	48,599	718	517
chrA05	1,463	69	944,426	36,826	646	534
chrA06	1,523	83	1,069,690	29,361	702	354
chrA07	1,542	98	1,121,157	33,009	727	337
chrA08	1,189	70	867,241	38,164	729	545
chrA09	2,533	113	1,697,547	40,655	670	360
chrA10	1,014	58	631,537	40,074	623	691
Total A genome	15,994	956	11,131,082	432,974		
chrC01	2,622	196	1,825,264	99,575	696	508
chrC02	3,026	85	2,208,383	27,450	730	323
chrC03	3,482	147	2,632,999	52,180	756	355
chrC04	3,073	115	2,406,990	42,593	783	370
chrC05	2,541	109	1,870,850	32,351	736	297
chrC06	2,255	89	1,632,474	34,029	724	382
chrC07	2,410	89	1,931,818	36,562	802	411
chrC08	2,121	98	1,658,128	34,555	782	353
chrC09	3,006	120	2,520,506	52,615	838	438
Total C genome	24,536	1,048	18,687,412	411,910		
Unplaced	3,245	187	1,989,049	127,721	613	683
Total	43,775	2,191	31,807,543	972,605		

Supplementary Table 8: Number of genes in predicted repetitive and collapsed regions in Darmor and Tapidor

Name	Darmor genes in repetitive regions	Tapidor genes in repetitive regions	Darmor genes in collapsed regions	Tapidor genes in collapsed regions
chrA01	380	26	114	163
chrA02	312	11	154	410
chrA03	470	17	212	363
chrA04	164	8	60	49
chrA05	257	9	75	59
chrA06	285	13	181	119
chrA07	164	9	45	51
chrA08	123	11	45	33
chrA09	377	11	189	140
chrA10	104	9	41	61
Total A genome	2636	124	1116	1448
chrC01	362	28	136	107
chrC02	381	6	177	245
chrC03	588	18	222	165
chrC04	291	13	111	105
chrC05	382	8	117	133
chrC06	152	7	65	73
chrC07	154	7	49	71
chrC08	132	8	46	70
chrC09	393	8	141	128
Total C genome	2835	103	1064	1097
Unplaced	232	19	275	106
Total	5703	246	2455	2651

Supplementary Table 9: Shared domains between Darmor genes located in repetitive regions and Tapidor genes located in collapsed regions (only ten r

Darmor		Tapidor	
Repetitive domains	Number of domains	Collapsed domains	Number of domains
WD domain, G-beta repeat	182	WD domain, G-beta repeat	59
Protein kinase domain	168	Protein kinase domain	57
PPR repeat	136	PPR repeat	55
PPR repeat family	110	RNA recognition motif. (a.k.a. RRM, RBD, or RNP domain)	44
RNA recognition motif. (a.k.a. RRM, RBD, or RNP domain)	109	Protein tyrosine kinase	38
Protein tyrosine kinase	96	PPR repeat family	35
ABC transporter	61	Myb-like DNA-binding domain	32
Myb-like DNA-binding domain	55	ABC transporter	32
Mitochondrial carrier protein	49	Mitochondrial carrier protein	24
Leucine rich repeat	47	Helicase conserved C-terminal domain	23

nost common domains)

Supplementary Table 10: Ten most common Swiss-Prot hits for repetitive genes in Darmor and collapsed genes in Tapidor with an e-value cutoff of 1

Darmor repetitive genes	Full name	Hit count	Tapidor collapsed genes	Full name	Hit count
sp|Q9LKB9|MYO6_ARATH	Myosin-6	12	sp|Q9FZ87|GH317_ARATH	Indole-3-acetic acid-amido synthetase GH3.17	7
sp|Q9LXT9|CALS3_ARATH	Callose synthase 3 (EC 2.4.1.34) (1,3-beta-glucan synthase)	12	sp|P0DKG4|MCC07_ARATH	MATH domain and coiled-coil domain-containing protein	7
sp|O22558|STY8_ARATH	Callose synthase 3 (EC 2.4.1.34) (1,3-beta-glucan synthase)	12	sp|Q9LYU4|GH312_ARATH	4-substituted benzoates-glutamate ligase	7
sp|F4I460|MYO8_ARATH	Myosin-8	11	sp|P0C0M3|GH311_ORYSJ	Probable indole-3-acetic acid-amido synthetase	7
sp|P46573|APK1B_ARATH	Protein kinase APK1B, chloroplastic	11	sp|Q9LV48|PERK1_ARATH	Proline-rich receptor-like protein kinase	6
sp|Q3B724|CALS5_ARATH	Callose synthase 5	11	sp|O22190|GH33_ARATH	Indole-3-acetic acid-amido synthetase GH3.3	6
sp|Q8RWL6|STY17_ARATH	Serine/threonine-protein kinase STY17	11	sp|P0DKG5|MCC08_ARATH	MATH domain and coiled-coil domain-containing protein	6
sp|Q9AUE0|CALS1_ARATH	Callose synthase 1	11	sp|F4I460|MYO8_ARATH	Myosin-8	5
sp|F4JTP5|STY46_ARATH	Serine/threonine-protein kinase STY46	10	sp|Q9LKB9|MYO6_ARATH	Myosin-6	5
sp|Q9S9V9|FBL23_ARATH	Putative F-box/LRR-repeat protein	10	sp|Q9M9M4|CSLD3_ARATH	Cellulose synthase-like protein D3	5

~

~

.e-5
